# Small regions as key sources of traditional knowledge: a quantitative ethnobotanical survey in the central Balkans

**DOI:** 10.1186/s13002-022-00566-0

**Published:** 2022-12-05

**Authors:** Pedja Janaćković, Milan Gavrilović, Milica Miletić, Maja Radulović, Stefan Kolašinac, Zora Dajić Stevanović

**Affiliations:** 1 Faculty of Biology, Department of Morphology and Systematics of Plants, University of Belgrade, Studentski Trg 16, 11 000 Belgrade, Serbia; 2 Faculty of Agriculture, Department of Agricultural Botany, University of Belgrade, Nemanjina 6, 11 000 Belgrade, Serbia

**Keywords:** Serbia, Medicinal plants, Food plants, Ritual uses, Veterinary uses, Cosmetic and craft uses

## Abstract

**Background:**

Starting from the idea that unexplored areas may yield new and different ethnobotanical information, we performed a survey of traditional uses of plants in two neighboring districts situated in east Serbia (Bor and Aleksinac), both lacking in previous ethnobotanical reports, but characterized by an interesting history and culture, together with some specific features. In this study, we hypothesized that such small and specific areas could be of high ethnobotanical importance.

**Methods:**

Semi-structured interviews were used with 155 informants. Relative cultural importance (RCI) indices, such as the frequency of citation (FC), relative frequency of citation (RFC), relative importance index (RI), informant consensus factor (ICF-FIC), use value (UV), fidelity level (FL) and Jaccard index (JI), were calculated, and principal coordinate analysis (PCoA) was performed.

**Results:**

In this study, 2333 use-reports and 114 plants were recorded. Of the 101 medical herbs, 33 are included in the European Pharmacopoeia Edition 8.0. The most frequently used mode of preparation was as an infusion (50.0%), while leaf (44.7%) was the most used plant part. The highest FC and RFC values were recorded for *Hypericum perforatum* L. (13.1 and 0.2, respectively), while the highest RI was documented for *Urtica dioica* L. (1.0). ICF and FL indices showed important differences among selected groups of informants. The PCoA showed three homogeneous plant groups. Plants were mostly used for the treatment of digestive (49.1%), circulatory (41.2%) and respiratory system disorders (35.1%). Thirty-seven (32.5%) herbs were used for human nutrition, 14 (12.3%) in veterinary medicine, 17 (14.9%) in rituals and ethnoculture, while 24 (21.0%) for miscellaneous purposes. The highest degree of similarity was determined with studies conducted in close proximity. Four species are new to Balkan ethnobotany. New uses for some well-known plants are highlighted.

**Conclusion:**

The study indicated that small and specific areas in the Balkans may be an important reservoir of ethnobotanical knowledge.

**Supplementary Information:**

The online version contains supplementary material available at 10.1186/s13002-022-00566-0.

## Background

Medicinal plants have an important significant role in the everyday life of rural people, particularly in developing countries. It is estimated that nearly 70 000 plant species are used for medicinal purposes today [1]. Wild plants are an important source of starting material for the synthesis of conventional drugs. About 80% of 122 plant-derived drugs are linked with their original traditional uses [2]. It was noted that 11% of the 252 essential drugs (listed by the World Health Organization—WHO) are exclusively of angiosperm origin [3]. Moreover, it is well established that most natural-based remedies exhibit fewer side effects compared to synthetic drugs. Collecting knowledge about plant species and their various uses is of great importance for the preservation of cultural heritage and the conservation of plant diversity [4].

The Balkan Peninsula is a biogeographic region with an exceptional floristic richness [5]. It is known that the *ca.* 8000 vascular plant species recorded for the Balkans include 2600 to 2700 endemics [6]. Serbia is located in the north-central region of the Balkan Peninsula and, according to recent data, has a flora of 4246 taxa [7] of which 1000 to 1500 species are used as foodstuffs, spices, food preservatives, medicinal plants, natural dyes or additives [8]. About 700 medicinal plant species are listed in Serbia [9]. People in Serbia have been relying on plants for various purposes since ancient times, as documented in old medieval Serbian therapy handbooks, known as the Hodosh Codex and the Chilandar Medical Codex [10, 11]. In many Balkan countries, people still practice herbal traditional medicine, where the purpose and way of use depend on cultural, historical and ethnic influences. However, depopulation, aging, migration, economic devastation and abandonment of villages and underdeveloped regions in Serbia and the entire Balkan region have resulted in a dramatic loss of ethnobotanical knowledge, in addition to a loss of plant genetic resource diversity [8]. In recent years, ethnobotanical investigations in Serbia were intensified [12–20]. Among the most recent studies, the differences in the traditional use of plants between rural and urban populations of different nationalities in the central Balkan region were reported [21], highlighting the value of traditional knowledge and of old practices which are still performed. Due to multiethnicity and complex historical background, Serbia represents a reservoir of cultural, linguistic, religious and other diversities. We consider that there are still many unexplored areas or regions with hitherto unrecorded ethnobotanical information, useful species, and new medicinal uses for known herbs.

Starting from the idea that unexplored areas may yield new and different ethnobotanical information, we performed a survey of traditional uses of plants in two neighboring districts situated in east Serbia (Bor and Aleksinac), both lacking in previous ethnobotanical reports, but characterized by an interesting history and culture, together with some specific features (rural, abandoned, economically devastated and with high migration rate). In this study, we hypothesized that such small and specific areas could be of high ethnobotanical importance and we set the following goals: (1) to collect data on the traditional uses of wild plants for medicine, human and domestic animal nutrition, veterinary medicine, folk and religious rituals, ethnoculture and other purposes; (2) to use relevant ethnobotanical indices and appropriate statistical methods to evaluate the obtained data; (3) to compare ethnobotanical knowledge between people of the two districts, between usage of plants by men and women, as well between inhabitants of cities and villages; (4) to compare our results with other ethnobotanical studies of neighboring regions conducted in Serbia and Balkans; (5) to report on new species records and new use-records not previously reported for Serbia and the Balkans; and (6) to suggest possible ways in which valuable traditional botanical knowledge can be preserved as an important part of general cultural heritage.

## Methods

### Research area

The research was carried out in Aleksinac and Bor districts in Eastern Serbia. Aleksinac is located in the Aleksinac basin (Serbian: *Aleksinačka kotlina*), in the lower stream of the South Morava river [22]. The area is known for former coal mining and high resources of oil shale estimated at about two billion tons in total reserves [23]. However, the mine was closed after an accident occurred in 1989 [24], after which no mining was performed anymore. Aleksinac is very near to Sokobanja, known among the biggest medicinal plants collecting centers in Serbia. Bor district is located in Timok Krajina in the eastern Serbia [25]. Initially, Bor was a village, but over time, in the mid-twentieth century, it developed into an urban area. This area is known for its copper and gold deposits [26]. The opening of the mine in 1903 caused the development of Bor as an industrial center in east Serbia in the mid of the last century [25]. During a time, mining started to bring more losses than benefits and the area was gradually depopulated and marginalized. Quite recently, mines were bought by a Chinese strategic partner (Zijin Mining). Although both areas suffered certain environmental and biodiversity damages, it is supposed that local inhabitants still rely on traditional herbal remedies and folk medicine. According to data acquired by the census in 2011, both districts exhibit a strong depopulation trend. The number of inhabitants in the Aleksinac district is around 16.700 and they are quite exclusively of Serbian nationality (91.7%). In Bor district, there are nearly 34.200 residents of Serbian ethnic majority (72.9%). The so-called Vlachs (“Vlah” in Serbian) of Romanian origin represent the second biggest ethnic group (13.8%), whereas Roma, Macedonian and Romanian minorities are fairly less represented [27, 28]. It is thought that Vlachs still tightly adhere to their cultural customs, speaking both Vlach (Daco-Romanian varieties) and Serbian language. The Vlach minority was recognized as the ethnic group from the earliest censuses in Serbia—since 1959, and it is strictly linked to the eastern Serbia in difference to Romanian minority settled mostly in the north of the country [29]. Religion and rituals resemble Serbian traditional customs. They also celebrate family saints (“slava”) which are in accordance with Serbian Orthodox Church tradition [30]. The number of interviewed respondents corresponded with the total number of district inhabitants.

### Ethnobotanical survey

Two towns and nine surrounding villages of both regions were included in the study. In the Aleksinac district, the city Aleksinac and the surrounding villages Jakovlje, Kamenica, Loznac and Ljupten were surveyed. In the Bor district, the city Bor and the surrounding villages Brestovac, Bučje, Krivelj, Oštrelj and Slatina were investigated. The research was conducted during May–July 2019. Groups of local inhabitants in both municipalities (155 in total; 55 informants from Aleksinac and 100 informants from Bor region) were interviewed using semi-structured questionnaires. The proportion of respondents from the two districts clearly corresponded with the total number of their inhabitants. The youngest and the oldest respondents were at age of 23 and 86, respectively. In total, 113 were women and 42 were men. Respondents were chosen with no special selection criteria. Middle-aged and older participants (which made up the majority of respondents) had a high level of experience in the use and application of wild plants and therefore were more willing to take part in our research. The local recipes for the preparation of herbal remedies were additionally recorded.

There is a difference between these two areas in the ethnicity of informants reflecting the general ethnic structure of surveyed regions. Population heterogeneity was more pronounced in Bor city and surrounding villages; 77 informants were Serbs, 19 were Vlachs, 2 were Macedonians, 2 were Bulgarians and 1 informant was Montenegrin. On the other hand, there is ethical uniformity in Aleksinac city and the surrounding area: out of the total of 55 interviewed inhabitants, 54 were Serbs and 1 was Montenegrin. Thanks to interviews, the data about the local names of plants, methods of collecting and primary processing of plant material, as well as plant parts in use and way of preparation of the herbal remedies, were recorded. The information on the traditional use of herbal drugs in folk and veterinary medicine, human and animal nutrition, traditional customs and folk beliefs, and uses of plants for other purposes was recorded. The plants were authenticated by Prof. Pedja Janaćković (the corresponding author of the current study), following the professional literature [31–35]. Local names were harmonized upon Simonović [36]. Each plant mentioned by the respondent was compared with a fresh specimen or with illustrations and photographs from referent literature sources to avoid errors related to the existence of different local names and misleading plant descriptions. The nomenclature of the species was compiled from contemporary checklists, monographs and databases, such as EURO + MED (Plantbase, http://ww2.bgbm.org/EuroPlusMed). Voucher specimens were deposited in the Herbarium of the University of Belgrade—Faculty of Biology, Institute of Botany and Botanical Garden “Jevremovac” (BEOU) (Table 1). Standard herbarium acronym follows Thiers B., 2019 + : Index herbariorum (http://sweetgum.nybg.org/science/ih/) [37].

**Table 1:** Recorded plant species in Aleksinac and Bor districts of eastern Serbia, their scientific names, affiliation to family, voucher numbers and vernacular names

Scientific name	Family	Voucher No. A: Aleksinac; B: Bor	Serbian folk name			Vlach folk name
			Aleksinac	Bor	Aleksinac/Bor	Bor
*Achillea clypeolata* Sm.	Asteraceae	B: (BEOU 17513)		Žuti ravan, žuta hajdučka trava		
*Achillea millefolium* L.	Asteraceae	B: (BEOU 17514) A: (BEOU 17597)	Jalova mesečina	Beli ravan, stolisnik, sporiš, romanika, bela hajdučka trava	Hajdučka trava, hajdučica	
*Aesculus hippocastanum* L.	Sapindaceae	B: (BEOU 17516)		Divlji kesten		
*Agrimonia eupatoria* L.	Rosaceae	B: (BEOU 17515) A: (BEOU 17598)	Vratika, čičke	Ranjenik, kostolom	Petrovac	
*Alcea biennis* Winterl	Malvaceae	A: (BEOU 17599) B: (BEOU 17518)	Slez			
*Alchemilla vulgaris* L.	Rosaceae	B: (BEOU 17517)		Virak		
*Allium ursinum* L.	Amaryllidaceae	A: (BEOU 17600)	Sremuš, skrembuš, cremuš			
*Althaea officinalis* L.	Malvaceae	A: (BEOU 17601)			Beli slez	
*Anthyllis vulneraria* L.	Fabaceae	B: (BEOU 17519)		Detelina kamenjarka		
*Arctium lappa* L.	Asteraceae	B: (BEOU 17520)		Čičak, čkalj		
*Arctium minus* (Hill) Bernh.	Asteraceae	A: (BEOU 17602)	Repuš			
*Arum maculatum* L.	Araceae	B: (BEOU 17521)		Kozlac		
*Asarum europaeum* L.	Aristolochiaceae	B: (BEOU 17522) A: (BEOU 17603)			Kopitnjak	Popilnik
*Asparagus officinalis* L.	Asparagaceae	B: (BEOU 17523)		Asparagus		
*Asplenium viride* Huds.	Aspleniaceae	A: (BEOU 17604)	Strašnik			
*Betula pendula* Roth	Betulaceae	B: (BEOU 17524) A: (BEOU 17605)			Breza	
*Calendula officinalis* L.	Asteraceae	B: (BEOU 17525) A: (BEOU 17606)			Neven	Ogršćanje
*Centaurium erythraea* Rafn	Gentianaceae	B: (BEOU 17526) A: (BEOU 17607)			Kičica	
*Chelidonium majus* L.	Papaveraceae	B: (BEOU 17527) A: (BEOU 17608)	Lišajevica, lišavica	Rosopas, lišajevka, rusa		
*Cichorium intybus* L.	Asteraceae	B: (BEOU 17528) A: (BEOU 17612)	Golotrba		Cikorija, vodopija, gologuza	
*Clematis vitalba* L.	Ranunculaceae	A: (BEOU 17609)	Loza, pautina			
*Cornus mas* L.	Cornaceae	A: (BEOU 17610) B: (BEOU 17529)		Drenjine	Dren	Koarnje
*Corylus colurna* L.	Betulaceae	B: (BEOU 17530)		Leska, lešnik		
*Cotinus coggygria* Scop.	Anacardiaceae	B: (BEOU 17531)		Ruj, rujevina		Skumpina
*Crataegus monogyna* Jacq.	Rosaceae	A: (BEOU 17611)	Crveni glog, glog			
*Cydonia oblonga* Mill.	Rosaceae	A: (BEOU 17613)	Dunja			
*Cynodon dactylon* (L.) Pers.	Poaceae	A: (BEOU 17614)				
*Datura stramonium* L.	Solanaceae	A: (BEOU 17615)	Tatula			
*Dipsacus laciniatus* L.	Caprifoliaceae	B: (BEOU 17532)	Češljuga			
*Epilobium parviflorum* Schreb.	Onagraceae	B: (BEOU 17533)		Mala mlečika		
*Equisetum arvense* L.	Equisetaceae	B: (BEOU 17534) A: (BEOU 17618)	Ženski rastrg, štukavac	Preslica, poljski rastavić, konjski rep	Rastavić	
*Equisetum telmateia* Ehrh.	Equisetaceae	A: (BEOU 17619)	Rastreg			
*Eupatorium cannabinum* L.	Asteraceae	B: (BEOU 17535)		Resnik, konopljuša, ustuk		
*Euphrasia officinalis* L.	Scrophulariaceae	B: (BEOU 17536)		Vidac, vidova trava		
*Filipendula hexapetala* Gilib.	Rosaceae	B: (BEOU 17,537)		Suručica		
*Fragaria vesca* L.	Rosaceae	B: (BEOU 17538) A: (BEOU 17621)			Divlja jagoda	
*Galium aparine* L.	Rubiaceae	A: (BEOU 17622)	Privaćuša			
*Galium odoratum* (L.) Scop.	Rubiaceae	B: (BEOU 17539)		Lazarkinja		
*Galium verum* L.	Rubiaceae	B: (BEOU 17540)		Ivanjsko cveće, ivančica		Smzijana
*Geranium macrorrhizum* L.	Geraniaceae	B: (BEOU 17541)		Zdravac		
*Geranium robertianum* L.	Geraniaceae	B: (BEOU 17542)		Smrdljivi zdravac, živa trava, crveni zdravac, devojačka trava, divlji zdravac		
*Hedera helix* L.	Araliaceae	B: (BEOU 17543) A: (BEOU 17623)			Bršljan	
*Helianthus tuberosus* L.	Asteraceae	A: (BEOU 17624)	Svinjski krompir			
*Helleborus odorus* Waldst. & Kit. ex Willd.	Ranunculaceae	B: (BEOU 17544)			Kukurek	
*Hieracium pilosella* L.	Asteraceae	B: (BEOU 17545)		Zečja loboda, lišajivica		
*Humulus lupulus* L.	Cannabaceae	B: (BEOU 17546)		Hmelj,divlji hmelj		
*Hypericum perforatum* L.	Hypericaceae	B: (BEOU 17547) A: (BEOU 17626)			Kantarion	
*Juglans regia* L.	Juglandaceae	A: (BEOU 17627)	Orah			
*Kickxia elatine* (L.) Dumort.	Plantaginaceae	A: (BEOU 17628)	Posečotina			
*Laserpitium latifolium* L.	Apiaceae	B: (BEOU 17548)		Raskovnik		
*Linaria vulgaris* Mill.	Plantaginaceae	B: (BEOU 17549)		Lanilist, žuta zevalica, bogorodičin lan, lančić		
*Loranthus europaeus* Jacq.	Loranthaceae	A: (BEOU 17629)	Imela			
*Lotus corniculatus* L.	Fabaceae	B: (BEOU 17550)		Zvezdan		
*Lysimachia nummularia* L.	Primulaceae	B: (BEOU 17551)		Metilj trava		
*Lythrum salicaria* L.	Lythraceae	B: (BEOU 17552) A: (BEOU 17630)			Potočnjak	
*Malus sylvestris* (L.) Mill.	Rosaceae	B: (BEOU 17553) A: (BEOU 17631)			Divlja jabuka	Korikove
*Malva sylvestris* L.	Malvaceae	A: (BEOU 17632)	Crni slez			
*Melilotus albus* Medik.	Fabaceae	B: (BEOU 17554)		Beli kokotac		
*Melilotus officinalis* (L.) Pall.	Fabaceae	B: (BEOU 17555)		Žuti kokotac		
*Melissa officinalis* L.	Lamiaceae	B: (BEOU 17556) A: (BEOU 17633)	Matočina		Matičnjak	
*Mentha longifolia* (L.) L.	Lamiaceae	B: (BEOU 17557) A: (BEOU 17634)		Divlja nana	Konjski bosiljak	
*Ononis spinosa* L.	Fabaceae	A: (BEOU 17635)	Grmotrn, zečji trn			
*Origanum vulgare* L.	Lamiaceae	B: (BEOU 17558) A: (BEOU 17636)		Divlji origano, vranilovka	Vranilova trava	
*Paliurus spina-christi* Mill.	Rhamnaceae	A: (BEOU 17637)	Čalije			
*Petasites albus* (L.) Gaertn.	Asteraceae	A: (BEOU 17638)				
*Petasites hybridus* (L.) “G. Gaertn., B. Mey. & Scherb”.	Asteraceae	B: (BEOU 17559)		Repuh, veliki podbel		Ropanj
*Peucedanum longifolium* Waldst. & Kit.	Apiaceae	B: (BEOU 17560)				Devesel
*Physalis alkekengi* L.	Solanaceae	A: (BEOU 17639)	Peruanska jabuka, petlidžančići			
*Pinus nigra* J. F. Arnold	Pinaceae	B: (BEOU 17561) A: (BEOU 17640)			Crni bor	
*Plantago lanceolata* L.	Plantaginaceae	B: (BEOU 17562) A: (BEOU 17641)	Dugačak žilovnik		Muška bokvica	
*Plantago major* L.	Plantaginaceae	B: (BEOU 17563) A: (BEOU 17642)	Žilovnik	Širokolisna bokvica, tegavac	Ženska bokvica, bokvica, žilovlak	
*Polygonum aviculare* L.	Polygonaceae	B: (BEOU 17565) A: (BEOU 17644)	Troska, troskavac	Svinjska trava	Troskot	
*Potentilla reptans* L.	Rosaceae	A: (BEOU 17643)				
*Prunella vulgaris* L.	Lamiaceae	A: (BEOU 17645)	Izdatljivka			
*Prunus spinosa* L.	Rosaceae	B: (BEOU 17566) A: (BEOU 17646)	Crni trn		Trnjina	
*Pulmonaria officinalis* L.	Boraginaceae	B: (BEOU 17564) A: (BEOU 17647)	Plućnik	Plućnjak		
*Pyrus pyraster* (L.) Burgsd.	Rosaceae	B: (BEOU 17567)		Divlja kruška		
*Quercus cerris* L.	Fagaceae	B: (BEOU 17568)		Hrast		
*Robinia pseudoacacia* L.	Fabaceae	B: (BEOU 17569)		Bagrem		Floran
*Rosa canina* L.	Rosaceae	B: (BEOU 17570) A: (BEOU 17648)	Šipkinje	Divlja ruža, šipurak	Šipak	Skobikur
*Rubus plicatus* Weihe & Nees	Rosaceae	B: (BEOU 17571)		Kupina, divlja kupina		Mura
*Rubus ulmifolius* Schott	Rosaceae	A: (BEOU 17649)	Zla kupina, divlja kupina			
*Rubus caesius* L.	Rosaceae	A: (BEOU 17650)	Divlja kupina			
*Rumex acetosa* L.	Polygonaceae	B: (BEOU 17572)		Kiseljak		
*Rumex crispus* L.	Polygonaceae	A: (BEOU 17651)	Štavelj, divlje zelje			
*Rumex patientia* L.	Polygonaceae	B: (BEOU 17573)	Štavelj, divlje zelje	Zelje, livadsko zelje		Dragaviju
*Salix alba* L.	Salicaceae	B: (BEOU 17574)		Vrba, bela vrba		
*Salix purpurea* L.	Salicaceae	A: (BEOU 17652)	Vrba, crvena vrba			
*Sambucus ebulus* L.	Adoxaceae	A: (BEOU 17653)	Burjan			
*Sambucus nigra* L.	Adoxaceae	B: (BEOU 17575) A: (BEOU 17654)	Bazovka		Zova	
*Satureja subspicata* Bartl. ex Vis.	Lamiaceae	B: (BEOU 17576)		Rtanjski čaj		
*Sempervivum tectorum* L.	Crassulaceae	B: (BEOU 17577)		Čuvarkuća		
*Sorbus aucuparia* L.	Rosaceae	B: (BEOU 17578)		Oskoruša		
*Stachys officinalis* (L.) Trevis	Lamiaceae	B: (BEOU 17579)		Ranilist		
*Symphytum officinale* L.	Boraginaceae	B: (BEOU 17580) A: (BEOU 17655)		Crni koren	Crni gavez, gavez	
*Tanacetum vulgare* L.	Asteraceae	B: (BEOU 17581)		Vratić, vrtika, povratić		
*Taraxacum* sect. *Ruderalia*	Asteraceae	B: (BEOU 17582) A: (BEOU 17660)			Maslačak	
*Teucrium chamaedrys* L.	Lamiaceae	B: (BEOU 17583) A: (BEOU 17656)			Podubica	
*Teucrium montanum* L.	Lamiaceae	B: (BEOU 17584)		Trava iva		
*Thymus serpyllum* L.	Lamiaceae	B: (BEOU 17585)		Majčina dušica, majkina dušica		
*Tilia platyphyllos* Scop.	Malvaceae	B: (BEOU 17586) A: (BEOU 17658)			Lipa	Ćij
*Tussilago farfara* L.	Asteraceae	B: (BEOU 17587) A: (BEOU 17659)	Podbel	Mali podbel		
*Trifolium pratense* L.	Fabaceae	B: (BEOU 17588)		Crvena detelina		
*Trifolium repens* L.	Fabaceae	B: (BEOU 17589)		Bela detelina		
*Urtica dioica* L.	Urticaceae	B: (BEOU 17590) A: (BEOU 17661)			Kopriva	Urdzk
*Vaccinium vitis-idaea* L.	Ericaceae	B: (BEOU 17591)		Brusnica		
*Valeriana officinalis* L.	Caprifoliaceae	B: (BEOU 17592)		Valerijana, odoljen, macina trava		
*Verbascum thapsus* L.	Scrophulariaceae	A: (BEOU 17662)				
*Verbena officinalis* L.	Verbenaceae	B: (BEOU 17593) A: (BEOU 17663)		Verbena, vrbena		
*Veronica officinalis* L.	Plantaginaceae	B: (BEOU 17594)		Razgon, veronika, čestoslavica		
*Vicia cracca* L.	Fabaceae	B: (BEOU 17595)		Grahorica		
*Viola odorata* L.	Violaceae	A: (BEOU 17664)	Divlja ljubičica			
*Xanthium spinosum* L.	Asteraceae	A: (BEOU 17665)	Bela boca			
*Xeranthemum cylindraceum* Sm.	Asteraceae	A: (BEOU 17596)	Divlja metla, metla			

No explicit rules or regulations pertain to the practice of ethnobotanical research in Serbia. The purpose, methodology and nature of the research were explained before starting the interviews and oral informed consent was obtained from all informants. Each participant in the study agreed to participate voluntarily. Participants were allowed to discontinue the interviews at any time. Upon completion of the study, all data are deposited in the phonothèque of the Department of Morphology and Systematics of Plants, University of Belgrade—Faculty of Biology. Thus, the ethnobotanical research and related activities, including collecting of plants, compiling databases, images, audio recordings, gathering information on the uses of traditional knowledge or other elements of biocultural heritage found in the study area, were undertaken in compliance with the International Society of Ethnobiology (ISE) code of ethics [38]. No harmful consequences (biological or cultural) for the local people and local communities arose from this research and its related activities. During the research, all principles of the code of ethics were adhered to including intellectual property rights and support for the development of local people’s cultures. All recommended standards for conducting and reporting ethnobotanical studies were considered in accordance with Weckerle and colleagues (2018) [39].

### Data analysis

Frequency of citation (FC) and relative frequency of citation (RFC).

The FC was calculated as follows:

FC = (Number of times a particular species was mentioned)/(total number of times that all species were mentioned) × 100.

The RFC index [40] was evaluated by dividing the number of informants who mentioned the use of the species (FC) by the total number of informants participating in the survey (N). The RFC index ranges from “0” when nobody refers to a plant as useful to “1” when all informants refer to a plant as useful. RFC = FC/N.

### Relative importance index (RI)

According to Tardío and Pardo-De-Santayana (2008) [40], this index was calculated with the following equation:

$${\text{RIs }} = \, \left\{ {{\text{RFCs}}\left( {{\text{max}}} \right) \, + {\text{ RNUs}}\left( {{\text{max}}} \right)} \right\}/{2}$$

where RFCs(max) is the relative frequency of citation over the maximum, i.e., it is obtained by dividing FCs by the maximum value in all species of the survey {RFCs(max) = FCs/max(FC)}, and RNUs(max) is the relative number of use-categories over the maximum, obtained by dividing the number of uses of the species by the maximum value in all species of the survey {RNUs(max) = NUs/max(NU)}. The RI index theoretically varies from 0, when nobody mentioned any use of the plant, to 1, when the plant was most frequently mentioned as useful in the maximum number of use-categories.

### Informant consensus factor (ICF-FIC)

To test the homogeneity of knowledge, the informant consensus factor was used [41], as follows:

$${\text{ICF}} = \frac{{{\text{nur}} - {\text{nt}}}}{{{\text{nur}} - 1}}$$

where nur refers to the number of use-reports for a particular use category and *nt* refers to the number of taxa used for a particular use category by all informants. ICF values are low (near 0) if plants are chosen randomly or if there is no exchange of information about their use among informants, and approach one (1) when there is a well-defined selection criterion in the community and/or if the information is exchanged between informants [42].

### Use value (UV)

Using the results obtained in the general interview, the use value (UV) of the plant species was calculated following [43–45] methods with some modification, using the following formula:

$${\text{UV }} = \Sigma {\text{U}}/{\text{n}},$$

where UV = use value of a species, U = number of quotations per species, and n = number of informants.

The use values are aggregated per plant part usage (counted as one in a certain category (a medicinal use, human nutrition, domestic animal nutrition, veterinary medicine, beliefs and contemplation and other purposes) regardless of different effects or uses. In other words, we did not aggregate statements for specific plant species per category, due to the nature of raw data from our study. We modified the earlier methodology in this way: if the same person cited the same plant but a different plant part or type of preparation in a certain category we mentioned that as a separate statement, because in this way a better insight into the importance of the use value of plants is gained.

The use value for each species can be calculated as the ratio of the number of citations to the total number of respondents.

where “U” refers to the number of uses mentioned by the informants for a given species and “n” refers to the total number of informants interviewed.

If a plant secures a high UV score that indicates there are many use-reports for that plant, while a low score indicates fewer use-reports cited by the informants.

### Fidelity level (FL)

The percentage of informants claiming the use of a plant species for the same major purpose was estimated using the Fidelity level index as determined by the following formula:

$${\text{FL}} = \frac{{{\text{lp}}}}{{{\text{lu}}}}\;{\text{x 1}}00$$

where *lp* denotes the number of informants who indicate the use of a species for the same major ailment and *lu* refers to the total number of informants who mentioned the same plant for any other use [46]. High FLs are obtained for plants which are used in the same way according to the majority of informants. Only species with the lp greater than or equal to 5 and FL greater than or equal to 0.2 were considered.

### Principal coordinate analysis (PCoA)

The principal coordinate analysis (PCoA) was used to test the relationships between objects (plants) and their uses, i.e. health-related disorder or medical.

To conduct PCoA, the dataset was systematized using the presence–absence matrix (1 and 0) with objects (plant taxa) in rows and categorical variable (health disorder/condition) in columns. As a result, the matrix 99 × 15 (number of species x number of illness) was obtained. This matrix is used to compute the similarity matrix based on Sokal and Sneath association coefficient (2) [47]:

$${\text{d}}_{{{\text{ij}}}} = \frac{2a + 2d}{{2a + b + c + 2d}},$$

where **d**_**ij**_ is the similarity between species **i** and **j**, **a** is the number of variables where **x**_**i**_ = presence and **x**_**j**_ = presence, **b** is the number of variables where **x**_**i**_ = absence and **x**_**j**_ = presence, **c** is the number of variables where **x**_**i**_ = presence and **x**_**j**_ = absence and **d** is the number of variables where **x**_**i**_ = absence and **x**_**j**_ = absence (Table 2).

**Table 2:** Frequency of four possible combinations for two binary variables

	*x* = presence	*y* = absence	Sum
*x* = presence	A	B	a + b
*y* = absence	C	D	c + d
Sum	a + c	b + d	a + b + c + d

The similarity is **1** if two species share all 15 descriptors and similarity is **0** if two species do not share any descriptor. Based on the similarity matrix, PCoA is conducted.

All the above-mentioned analyses were performed using XLSTAT 2014 software (Addinsoft, NY, USA).

### Jaccard index (JI)

This index is used to compare the present study data with the data of other ethnobotanical studies conducted in neighboring and other regions in Serbia. The formula used to evaluate the JI index [48] is as follows:

$${\text{JI}} = {\text{cx1}}00/{\text{a}} + {\text{b}} - {\text{c}},$$

where “a” is the recorded number of species of the study area “A,” “b” is the documented number of species of the area “B” and “c” is the common number of species in both area “A” and “B.” In the case of local communities, “a” is the number of species reported by a local community “A,” “b” is the number of species cited by the local community “B” and c is the number of species reported by both “A” and “B.”

## Results and discussion

### Demography of informants

A total of 155 informants were interviewed. Out of these, 42 (27.1%) were male and 113 (72.9%) were female. The informants were categorized into five different age groups, as documented in Table 3.

**Table 3:** Demographic characteristics of informants

Factor	Categories	Aleksinac		Bor		Total no. of persons	Percentage (%)
		City	Villages	City	Villages		
Sex	Male	1	20	7	14	42	27.1
	Female	9	25	43	36	113	72.9
Age	≤ 30	0	2	1	0	3	1.9
	31–40	1	1	1	1	4	2.6
	41–50	0	5	6	6	17	10.9
	51–60	2	7	8	18	35	22.6
	** > **60	7	30	34	25	96	61.9
Nationality	Serbs	9	45	45	32	131	84.5
	Vlach	–	–	2	17	19	12.3
	Bulgarians	–	–	1	–	1	0.6
	Montenegrins	1	–	1	–	2	1.3
	Macedonians	–	–	1	1	2	1.3

### Mode of preparation

The most frequently used mode of preparation was as an infusion (50.0%) followed by processed (12.9%), fresh (direct utilization) (10.1%), tincture (4.7%), balm (4.4%) and so on (Tables 4, 5 and 6 and Additional file 1: Table 1), which was also reported by ethnobotanical studies performed in the closest neighborhood regions [13, 15, 18]. The most used plant part (Additional file 1: Table 2) was the leaf (44.7%).

**Table 4:** Medical uses of plant species of the Aleksinac and Bor districts of eastern Serbia

Scientific name	Part of the plant			Type of preparation			Medicinal purposes		
	Aleksinac	Bor	Aleksinac/Bor	Aleksinac	Bor	Aleksinac/Bor	Aleksinac	Bor	Aleksinac/Bor
*Achillea clypeolata*		Flowers			Infusion			Maintaining general health condition, heart disorders, pulmonal diseases, stomach ailments, respiratory tract problems	
*Achillea millefolium*^♦^		Whole plant^#^			Balm			Skin diseases	
					Ointment			Skin diseases	
			Aerial parts		Balm			Excessive bleeding, wounds and burns healing	
					Tincture			Varicose veins, circulation improvement	
						Infusion	Against inflammation and stomach disorders, calming effect	Headache, rheumatism, liver, pancreas and gallbladder ailments, respiratory system disorders, antibiotic	
						Tincture	Wound healing, massaging sore spots, reducing swelling after insect's sting	Rheumatism, liver, pancreas, gallbladder ailments, respiratory system disorders, antibiotic	
	Flowering apical parts			Infusion			Against *enuresis nocturna*, bladder and gynecological disorders		
		Flowers and leaves^#^			Infusion			Immune system strengthening, detoxification, against myoma and stomach diseases, improves appetite	
		Leaves^#^			Balm			Skin care, hemorrhage, wound healing	
					Juice from leaves			Wound healing	
					Tincture			Stomach disorders	
					Powder			Hemorrhage, healing bleeding wounds	
					Tincture			Varicose veins and circulation improvement	
			Flowers^#^		Tincture			Varicose veins and circulation improvement	
						Infusion	Wound rinse, bladder ailments, ovary diseases, antipyretic, reduction in excessive urination, anti-inflammatory activity, general health condition improvement	Throat and wound rinse, stomach ulcer, skin disorders, immune system strengthening, nausea, headache, migraine, asthma, reducing blood sugar levels, blood detoxification and strengthening, detoxification, nervous system diseases, blood pressure regulation, inhibition of blood clot formation, vaginal flushing, various rashes, liver, intestine and gallbladder disorders, healing bleeding wounds, cough, *medical panacea,* rash, purulent wounds	Gastric and respiratory disorders, bronchitis, menstrual cycle regulation
				Tincture			Wound healing		
*Aesculus hippocastanum*		Flowers			Infusion			Against neuralgia, rheumatism, improving circulation	
					Oil extract			Rheumatism	
		Fruits			Tincture			Varicose veins, improving circulation	
					Tincture			Varicose veins, rheumatism	
		Fruits with capsule			Balm			Veins disorders	
*Agrimonia eupatoria*^♦^		Aerial parts			Infusion			Lowering blood pressure, against headache, throat inflammation and oral cavity wounds treatment by rinsing, urinary system, liver, gallbladder and stomach disorders, kidney stones	
			Flowers and leaves^#^			Infusion	Urinary tract infections	Detoxification	Kidney disorders
			Leaves^#^			Infusion		Liver, spleen and heart ailments, against headache and sore throat, kidney and bladder disorders	Urinary system ailments
*Alcea biennis* *	Leaves			Infusion			Intestinal diseases, inhalation against sinusitis		
*Alchemilla vulgaris*^♦^		Leaves^#^			Infusion			Gynecological ailments, against vaginal discharge	
		Flowers and leaves^#^			Infusion			Gynecological ailments	
*Allium ursinum*	Leaves			Tincture			Blood pressure regulation and reduction in elevated blood pressure		
*Althaea officinalis*^♦^	Aerial parts^#^			Infusion			Bronchitis, dry cough		
			Roots			Maceration	Bronchitis, dry cough	Expectoration, throat inflammation, cough, respiratory tract disorders	
				Infusion			Against airways obstruction, against cough, expectoration, pulmonary diseases		
*Anthyllis vulneraria*		Aerial parts			Infusion			Blood cleansing	
		Leaves			Fresh, revetment			Wound healing	
*Arctium lappa*		Leaves			Fresh, revetment			Painful spots, rheumatism, joint inflammation	
					Infusion			Against cough, diarrhea	
		Roots			Infusion			Elimination of heavy metals from organism, cancer cell inhibition, detoxification, reducing blood sugar levels	
					Tincture			Rheumatism	
*Arctium minus*	Leaves			Fresh, revetment			Heated on a stove or coated with vegetable oil for painful joints		
*Arum maculatum*		Rhizome			Fresh, mixed with honey			Intestine ailments, digestion improvement, against hemorrhoids	
*Asarum europaeum*		Leaves			Fresh, revetment			Purulent wounds	
					Infusion			Kidney ailments	
*Asparagus officinalis*		Roots			Infusion			Urinary tract ailments, kidney stones	
*Asplenium viride* *	Leaves			Infusion			To cure fright		
*Betula pendula*^♦^			Flowers^#^			Infusion	Male flowers are used for urinary tract and prostate ailments	Male flowers are used for detoxification, eliminating salt excess from blood vessels	
		Buds#			Infusion			Kidney diseases, eliminating kidney sand and limescale	
			Leaves			Infusion	Urinary tract infections	Kidney diseases, eliminating kidney sand, limescale and stones, urinary ducts inflammation, kidney cleansing	Urinary tract diseases
*Calendula officinalis*^♦^		Aerial parts^#^			Infusion			Intestinal mucosa and intestine inflammation, calming effects, liver and gallbladder inflammations, mucosa and intestine inflammation	
					Tincture			Intestinal mucosa and intestine inflammation	
			Flowers			Infusion	Against cold, detoxification, blood strengthening, blood vessels flexibility maintenance, bladder disorders	Blood cleansing, against ovary and breast cysts, cancer, kidneys diseases, circulation improvement	
				Tincture			Tumor prevention		
					Balm			Wounds and burns healing, skin problems, eczema, hand care, knee pain, injures	
				Ointment			Hemorrhoids, wounds and burns healing, inflammations		
					Tincture			Varicose veins, circulation improvement	
	Leaves^#^			Fresh, revetment			Purulent wounds, stings		
*Centaurium erythraea*^♦^			Aerial parts			Infusion	Calming effect, positive effect on stomach	Liver, pancreas and stomach diseases, blood vessels function regulation, pulmonary ailments, reducing blood sugar levels, appetite improvement, gallbladder ailments, blood cleansing, diabetes, heartburn, irregular menstrual cycle	
					Tincture			Diabetes, liver ailments	
		Flowers and leaves^#^			Infusion			Gastric function regulation	
		Flowers^#^			Infusion			Stomach diseases, diabetes, reducing blood sugar levels, blood vessels cleansing, fever	
*Chelidonium majus*^♦^	Aerial parts			Maceration			Wounds rinse		
	Apical parts			Maceration			Benign tumor treatment		
		Flowers and leaves^#^			Tincture			Cells regeneration	
			Leaves^#^	Fresh			Ovaries and uterus cysts		
					Fresh, revetment			Eyesight improvement	
					Tincture			Against cancer	
					Bath			Painful legs, circulation improvement	
						Infusion	Strengthening immune system after tumors	Against cancer and metastasis, myoma, jaundice, gastric, liver, gallbladder treatment, ulcers, stomach disorders, psoriasis, gynecological problems, intestine function improvement, skin redness	
			Latex^#^			Fresh	Corn removal	Keratosis, face, cleansing, aging, hyperpigmentation, cataract	Against warts, skin ailments
*Cichorium intybus*	Whole plant			Decoction			Against diarrhea and dysentery		
		Aerial parts			Infusion			Against diarrhea, reducing blood sugar levels, jaundice, cirrhosis, weight regulation, liver cleansing, stomach, liver, gallbladder ailments	
		Peduncle			Infusion			Against diarrhea	
		Flowers			Infusion			Against diarrhea, stomach diseases	
		Flowers and roots			Infusion			Against diarrhea	
			Roots		Infusion			For better digestion, weight loss, against diarrhea	
				Decoction			Kidneys ailments, kidney sand, against diarrhea		
*Cornus mas*			Fruits		Infusion			Heart disorders, digestive system disorders, blood strengthening, blood pressure regulation, complete blood count improvement, cold, diarrhea	
						Tincture	Positive effect on whole organism	Nausea, gastric ailments, diarrhea	
					Juice proveriti			Regulation of free toxic radicals in organism	
*Corylus colurna*		Leaves			Fresh			Against swelling after snakebite	
*Cotinus coggygria*		Twigs			Infusion			Burns, wounds on the feet and hands, increased concentration of sugar in the blood, gynecological problems, cells regeneration, prevention of metastases, hemorrhoids, kidney ailments, high blood pressure	
					Tincture			Against cancer	
		Bark			Infusion			Reducing gastric acid, mouthwash, cleansing the body of toxins, treatment of cancer, wound rinsing, against cancer, eczema rinse and intestine ailments	
		Leaves			Infusion			Reducing gastric acid, mouthwash, cleansing the body of toxins, treatment of cancer	
*Crataegus monogyna*^♦^	Fruits			Decoction			Blood pressure lowering		
	Flowers			Infusion			Heart disorders, cardiovascular diseases treatment		
	Leaves			Infusion			Heart disorders		
	Bark^#^			Decoction			Heart disorders		
*Cydonia oblonga*	Leaves			Decoction			Against diarrhea		
*Cynodon dactylon*	Whole plant			Infusion			Against hemorrhoids		
	Aerial parts			Infusion			Varicose veins treatment		
	Roots			Infusion			Expectoration, kidney sand, urinary infections, intestine cleansing		
*Datura stramonium*^♦^	Leaves			Fresh			Wounds		
*Epilobium parviflorum*		Aerial parts			Infusion			Problems with the bladder, enlarged prostate, reducing pain in the bladder, as a diuretic, kidney diseases	
		Flowers			Infusion			Bladder inflammation, enlarged prostate	
*Equisetum arvense*^♦^			Non-fertile aerial parts			Infusion		Urinary ducts ailments and inflammation, joints diseases, maintaining mineral balance, osteoporosis, urinary tract problems, diarrhea, detoxification, pulmonary and kidney cleansing, regulating uric acid level in blood, bladder sand and disorders, stopping nose bleeding, sclerosis, cancer prevention	Kidney diseases
					Tincture			Massaging tired legs and arms	
			Fertile aerial parts^#^		Infusion		Urinary tract inflammation, urinary tract cleansing, kidney ailments, kidney sand elimination, bladder ailments, reduction in frequent urination, genital inflammation in women		
*Equisetum telmateia*	Apical parts			Decoction			Decoction prepared together with corn silk and young ears of corn is used against kidney and urinary tract ailments		
*Eupatorium cannabinum*		Whole plant			Infusion			Metabolism regulation, hormone stabilization	
					Tincture			Osteoporosis	
		Aerial parts			Infusion			Cholesterol level regulation	
		Flowers			Infusion			Kidney problems, metabolism regulation	
*Euphrasia officinalis*		Whole plant			Infusion			Eye treatment	
		Aerial parts			Infusion			Treatment of eye diseases, cataracts	
*Filipendula hexapetala*		Flowers			Infusion			Blood vessels strengthening, stimulation, preventing cardiac arrest, stomach disorders treatment, heart disorders hypertension, treatment of kidney inflammation, as a diuretic	
		Leaves			Infusion			Liver and kidney disorders	
		Roots			Infusion			Respiratory tract disorders	
*Fragaria vesca*	Flowers and leaves			Infusion			Immune system strengthening		
			Leaves		Fresh			Stomach ailments	
						Infusion	Heart disorders, general health condition improvement	Stomach problems, liver cleansing, against diarrhea, against cough, menstrual problems	
*Galium odoratum*		Flowers			Infusion			Respiratory tract inflammation, diuretic, blood cleansing, against migraine	
*Galium verum*		Aerial parts			Infusion			Against cancer, kidney problems, thyroid disorders, stomach diseases, headache, liver inflammation, respiratory tract disorders, hormonal stabilizer, regulation of female and thyroid hormones	
					Balm			Skin diseases	
		Flowers			Infusion			Bronchitis, pulmonary diseases	
					Balm			Skin cancer	
*Geranium robertianum*		Aerial parts			Infusion			Mouth and throat inflammation	
					Tincture			Varicose veins, circulation improvement	
		Leaves			Infusion			Hormone regulation	
					Tincture			Hormone regulation	
*Hedera helix*^♦^		Twigs^#^			Infusion			Hemorrhoids	
		Fruits^#^			Tincture			Rheumatism	
			Leaves			Infusion	Bronchitis, sore throat, against cough	Respiratory ailments, bladder inflammation, kidney and bladder stones and sand, hemorrhoids	
					Tincture			Respiratory ailments, rheumatism	
					Oil extract			Eczema	
*Helianthus tuberosus*	Tuber			Fresh, revetment			Mumps		
*Hieracium pilosella*		Whole plant			Infusion			Bladder inflammation, treatment of urinary disorders	
*Humulus lupulus*^♦^		Fruits^#^			Infusion			Mental diseases, calming effect, insomnia	
*Hypericum perforatum*^♦^			Aerial parts			Infusion	Improve general health condition, stomach ailments	Calming effects, respiratory system disorders, depression	Stomach problems
					Tincture			Depression, stomach ailments	
					Balm			Wound healing, burns, scratches, cuts	
				Oil extract			Wound healing		
	Flowering apical parts			Infusion			Stomach disorders, gastric ailments, gallbladder stones, antibacterial activity, gastric and intestine ulcer		
				Oil extract			Wounds, cuts, burns, gastric ulcer		
			Flowers^#^			Infusion	Gastritis, gastric disorders, cough, general health condition improvement, hemorrhoids, private areas rinse	Heartburn, gallbladder ailments, mouthwash, strengthening immunity, calming effect, pulmonary diseases, depression, gallbladder stones, detoxification, gynecological ailments, kidney sand, *medical panacea*, digestive system disorders, general health condition improvement, throat rinsing against bed wetting, infections, bladder inflammation and diseases, cold	Gastric ulcer, stomach pain, urinary tract infections, throat inflammation
				Tincture			Wounds, calming effect in stressful situations		
				Oil extract			Wounds, burns, skin diseases, problematic moles, improve general health condition, bruises, cuts, against vaginal discharge, inflammation, gynecological ailments, surgery incision healing, scars, hemorrhoids, muscle spasm		
					Balm			Burns, decubitus wounds, cuts, face eczema, scratches, injuries, bee and wasp stings, wounds healing	
		Flowers and leaves^#^			Oil extract			Wounds	
					Balm			Burns, wounds	
*Juglans regia*	Fruits			Tincture			Unriped fruits are used for regulation of thyroid gland, source of iodine, against gastric inflammation		
	Leaves			Infusion			Function regulation of thyroid gland		
				Bath			Treatment of barren, pain release, bone diseases		
*Kickxia elatine* *	Whole plant			Infusion			Against *enuresis nocturna* in children		
	Aerial parts			Fresh, revetment			Skin cuts and wounds treatment		
*Linaria vulgaris*		Aerial parts			Tincture			Varicose veins, circulation improvement	
		Flowers			Infusion			Detoxification, liver and spleen ailments, elimination of excess water from organism	
					Tincture			Varicose veins, circulation improvement	
*Loranthus europaeus*	Leaves			Infusion			Blood pressure regulation, circulation improvement		
*Lythrum salicaria*^♦^	Flowering aerial parts			Infusion			Against diarrhea in children		
		Apical parts			Fresh, revetment			Against itch, skin redness	
*Malus sylvestris*			Fruits			Vinegar	Reducing cholesterol levels in blood	Varicose veins, fever, pain in legs	
*Malva sylvestris*^♦^	Flowers and leaves			Infusion			Against cough		
*Melilotus albus*		Aerial parts			Tincture			Varicose veins, circulation improvement	
*Melilotus officinalis*^♦^		Aerial parts			Infusion			As diuretic, against migraine, respiratory tract disorders, regulation digestion, liver and gallbladder disorders	
					Tincture			Varicose veins, circulation improvement	
*Melissa officinalis*^♦^			Aerial parts^#^			Infusion	Against cold, calming effect	Stomach disorders, painful and irregular menstruation, sleep improvement, positive effect on nervous system	
		Apical parts^#^			Infusion			Calming effect	
		Flowers^#^			Infusion			Heart function regulation	
		Flowers and leaves^#^			Infusion			Immune system strengthening	
		Juice from leaves^#^						Dried and irritated skin	
			Leaves			Infusion		Against insomnia, relaxing effect, nausea, sleep improvement, mental illness, nervousness, tiredness, heart ailments, pulmonary ailments	Calming effect, stomach disorders
*Mentha longifolia*		Aerial parts			Infusion			Stomach ailments	
					Tincture			Stomach ailments	
	Flowering apical parts			Infusion			Stomach disorders		
		Leaves			Infusion			Against insomnia, nausea, stomach ailments, calming effect, pharynx inflammation, sinusitis	
*Ononis spinosa*^♦^	Roots			Infusion			Heart disorders		
				Decoction			Eliminating kidney sand and gallbladder stones		
*Origanum vulgare*^♦^			Aerial parts			Infusion	Calming effect, improve appetite	Against cough, cold, relaxing effects, detoxification, against bacteria *Escherichia coli* in urine, urinary ducts infections, triglycerides levels regulation, antifungal effect against *Candida*, regulation fat, chest pain	Urinary tract inflammation
	Flowering apical parts						Calming effect		
		Flowers			Infusion			Against cold, cough, throat inflammation, stomach problems, calming effect	
		Flowers and leaves			Infusion			Relaxing effect, detoxification, disease prevention, gastric problems	
		Leaves			Infusion			Lowering gastric acid levels	
*Paliurus spina-christi*	Fruits			Infusion			Against diarrhea		
Petasites albus*	Whole plant			Decoction			Urinary tract inflammation		
*Petasites hybridus*		Leaves			Fresh, revetment			Painful spots, back and knee pain, joint dislocation, sprains	
		Roots			Tincture			Varicose veins, circulation improvement	
*Physalis alkekengi*	Fruits			Fresh			Ear pain		
				Maceration			Ear pain		
*Pinus nigra*		Shoots			Syrup			Made from young shoots is used for pulmonal diseases	
	Pollen			Mixed with honey			Pneumonia, bronchitis, particularly in children, expectoration		
		Leaves			Infusion			Respiratory tract disorders, bronchitis	
					Bath			Detoxification, painful legs, circulation improvement	
*Plantago lanceolata*^♦^	Whole plant^#^			Infusion			Throat and tonsils inflammation		
			Leaves	Fresh			Cuts, wounds, pus removal		
						Infusion	Gastric ulcer, general health condition improvement	Gastritis, pulmonal diseases, urinary tract disorders, prostate problems	
						Mixed with honey		Pulmonal diseases	Expectoration
*Plantago major*		Juice from leaves			Fresh, revetment			Injuries, reducing swelling after bee sting, wounds	
			Leaves		Fresh			Heartburn, gastritis	
						Fresh, revetment	Ingrown hairs, skin ulcers	Burns, callus on heel, injuries, swollen feet, painful spots, clavus, sting mosquitoes, rheumatism, hemorrhage, animal bites, cuts, skin inflammation	Purulent wounds, wound healing, inflammation after sting
						Infusion	Stomach pain, against frequent urination, general health condition improvement	Nausea, stomach ailments, cough, pulmonary diseases, kidney and bladder ailments, sore throat, against bacteria *Escherichia coli*, expectoration, gastric mucosa regeneration, digestion improvement, asthma, bronchitis	Gastric ulcer
					Fresh mixed with honey			Stomach problems, pulmonary diseases, expectoration, vocal cords ailments	
					Balm			Decubitus wounds	
*Polygonum aviculare*^♦^		Whole plant^#^			Infusion			Heart disorders, kidney disorders, kidney and gallbladder stones	
			Aerial parts			Infusion	Bladder disorders, treatment of polycystic ovaries, improving brain circulation	As diuretic, rheumatism, kidney cysts, pulmonary disorders, stomach pain	
*Potentilla reptans*	Leaves			Infusion			Against diarrhea		
*Prunella vulgaris*^♦^	Aerial parts^#^			Infusion			Stomach diseases		
	Flowering apical parts^#^			Infusion			Eyewash		
*Prunus spinosa*			Fruits			Fresh			Stomach problems
					Infusion			Asthma, anemia, against diarrhea, stomach diseases, complete blood count improvement	
					Wine			Anemia	
					Tincture			Strengthening immunity	
*Pulmonaria officinalis*		Aerial parts			Infusion			Pulmonary diseases, pneumonia, bronchitis	
		Flowers			Infusion			Pulmonary diseases	
		Flowers and leaves			Infusion			Pulmonary diseases	
			Leaves			Infusion		Pulmonary diseases, against cold, flu	Bronchitis
*Pyrus pyraster*		Leaves			Infusion			Against bacteria *Escherichia coli* in urine, urinary ducts infections	
*Quercus cerris*		Bark			Infusion			Strengthening immunity, respiratory tract problems, urinary ducts, stomach ailments	
*Robinia pseudoacacia*		Flowers			Infusion			Expectoration, cold, pulmonary diseases	
					Balm			Rheumatism	
*Rosa canina*^♦^			Fruits			Infusion		*Medical panacea*, against cold, immune system strengthening, kidney problems (elimination of sand and stone), calming effect, diarrhea, against virus, vitamin deficiency, fever, relaxing effects	Throat ailments, flu, cough
				Maceration			Source of vitamin C		
				Decoction			Immune system strengthening, cold, blood pressure lowering, fever		
					Tincture			Immune system strengthening, oral cavity rinse and disinfection	
*Rubus plicatus*		Fruits			Wine			Anemia, immune system strengthening	
		Leaves			Fresh, revetment			Wound cleansing and healing, rheumatic pain	
					Fresh			Stomach disorders	
					Infusion			Against cough, blood cleansing, immune system strengthening, respiratory tract disorders, stomach ailments, urinary tract problems, appendicitis	
*Rubus ulmifolius*	Flowers and leaves			Infusion			Immune system strengthening		
	Leaves			Fresh, revetment			Purulent wounds		
				Infusion			Heart and blood strengthening, cold, cough, throat and tonsils ailments		
*Rubus caesius*	Leaves			Infusion			Menstrual cycle regulation		
*Rumex acetosa*		Leaves			Fresh			Stomach problems	
*Rumex crispus*		Seeds			Infusion			Against diarrhea in children and adults	
*Rumex patientia*		Seeds			Infusion			Against diarrhea in children and adults	
*Salix alba*		Bark			Infusion			Lowering elevated body temperature, liver, spleen and gallbladder ailments, diuretic, against headache, bladder diseases	
*Salix purpurea*^♦^	Bark			Infusion			It is drunk after accidents, for bruises and wound healing		
				Decoction			Fever		
*Sambucus ebulus*	Roots			Decoction			As revetment, for gallbladder stone		
*Sambucus nigra*^♦^			Twigs^#^			Balm	Burns treatment	Wounds, scratches, wrinkles, acne, facial care, spider bite, allergies, hemorrhoids	
			Fruits^#^^s^		Fresh			Detoxification, complete blood count improvement	
					Wine			Pulmonary diseases, immune system strengthening	
			Flowers			Infusion		Asthma, flu, calming effect, throat ailments and inflammation, detoxification, respiratory tract problems, sweating improvement, expectoration, sneezing, pneumonia, stomach problems, strengthening immunity, blood pressure problems, relaxing effect	Against cough, cold, pulmonary problems, bronchitis, fever
					Syrup			Against cough, immune system strengthening	
		Flowers and leaves^#^			Infusion			Bronchitis, stomach diseases	
*Satureja subspicata*		Flowers and leaves			Infusion			Stomach problems, bronchitis	
		Aerial parts			Infusion			Against cold, gastric ailments, strengthening immunity, pulmonary diseases, against cough, stomach discomfort	
*Sempervivum tectorum* ▲		Leaves			Fresh			Against headache▲, lowering blood sugar, heartburn, stomach problems, lowering triglyceride levels cancer prevention	
					Fresh, revetment			Wounds, injuries, burns,	
					Fresh, mixed with honey			Liver problems, detoxification, against cysts (breast cysts) and tumors	
		Juice from leaves			Fresh			Ear pain, injuries, skin wounds, fat in the ears	
*Stachys officinalis*		Aerial parts			Infusion			Respiratory tract problems, detoxification, asthma, bronchitis, flatulence	
		Aerial parts and flowers			Infusion			Stomach diseases,	
		Flowers			Balm			Decubitus wounds	
		Flowers and leaves			Infusion			Regeneration of all cells in body, against cysts, stomach diseases, cleansing intestine	
		Leaves			Fresh, revetment			Wounds healing	
					Infusion			Stomach ailments, immune system strengthening, pulmonary diseases, against cancer, tumors, cysts on kidneys	
*Symphytum officinale*			Leaves		Fresh, revetment			Wounds, inflammation, gout, sprains, fracture, joint ailments	
					Balm			Sprains, joint ailments	
				Ointment			Treatment of closed wounds		Varicose veins
			Roots		Infusion			Rheumatism, heel callus removal, general health condition improvement, bones pain	
						Tincture	Joints inflammation, against leg pain, treatment of closed wounds, varicose veins	Rheumatism, osteoporosis, heel callus removal, all types of hematomas, meniscus injury, capillary problems, treatment of digestive system (intestine, stomach problems)	
					Ointment			Gout, against pain, varicose veins	
					Balm			Rheumatism, ligament injury, cartilage restoration, arthrosis, bone pain, muscle braking, joint dislocation, sprains	
					Fresh, revetment			Painful spots	
					Tincture			Varicose veins, regulation of circulation	
*Tanacetum vulgare*		Flowering apical parts of plant			Infusion			Improving appetite, stomach strengthening, kidney stones and sand elimination, diuretic	
		Leaves			Fresh, revetment			Placed on eyes and forehead for reducing headache	
*Taraxacum* sect*. Ruderalia* ^♦^▲		Flowers^#^			Infusion			Detoxification, liver, gastric, pulmonary ailments, face washing▲, jaundice▲	
					Tincture			Liver cleansing	
		Peduncle^#^			Fresh			Diabetes	
		Leaves^#^			Infusion			Menstrual cycle regulation, diuretic	
					Tincture			Wound rinse, endometrial polyps, positive effect on vocal cords▲	
					Fresh			Detoxification, heartburn, gastric cancer	
		Roots			Infusion			Blood strengthening, against breast cancer, stomach and liver diseases, throat ailments, problems with urinary ducts, appetite improvement, blood cleansing, menstrual cycle regulation, diuretic, detoxification, gynecological ailments, inflammations, immune system strengthening, against malignancies	
					Tincture			Liver cleansing and ailments, against malignancy	
					Powder			Liver ailments	
*Teucrium chamaedrys*▲			Aerial parts		Infusion			Stomach, pancreas, liver and spleen disorders, respiratory diseases, improving digestion, cataract treatment▲, eye ailments▲, against nausea, reducing blood sugar levels, weight loss, stomach acid reduction, gastric ulcer,	Gastric disorders
		Flowers			Infusion			Heartburn, gastric disorders, against cough, improving appetite	
		Flowers and leaves			Infusion			Gastric, liver and gallbladder disorders, improving digestion, blood pressure regulation, reducing blood sugar levels, against asthma	
*Teucrium montanum*		Whole plant			Infusion			Medical panacea	
		Aerial parts			Infusion			Detoxification, immune system strengthening, headache, stomach ailments, improving appetite, digestive and respiratory system disorders	
					Tincture			Improving appetite	
		Flowers and leaves			Infusion			For better appetite, against cold	
					Tincture			For better appetite, against cold	
*Thymus serpyllum*^♦^		Whole plant^#^			Infusion			Calming effect, digestion regulation, stomach ailments, *medical panacea*	
		Aerial parts			Infusion			Calming effect, headache, pneumonia, bronchitis, relaxing effect, pulmonary problems, cough, stomach ailments, respiratory tract disorders, vertigo, migraine, physical weakness, vaginal secret elimination, disinfection, asthma	
					Tincture			Massaging head against headache	
					Syrup			Immune system strengthening, cough	
		Flowers^#^			Infusion			Calming effect, blood vessels cleansing, digestion regulation, gastric and pulmonal disorders, throat inflammation, cough, bronchitis, detoxification	
					Oil extract			Relaxing effect	
		Flowers and leaves^#^			Infusion			Calming effect, against cold and cough	
*Tilia platyphyllos*^♦^			Flowers			Infusion	Improve general health condition, relaxing effect	Throat inflammation, sneezing, respiratory diseases, against bacteria and viruses, immune system strengthening, medical panacea, pulmonary ailments, sedative, circulation improvement,	Against cold, cough, flu, fever, sweating improvement for fever reduction, calming effect, insomnia
		Leaves^#^			Infusion			Improving sweating	
*Tussilago farfara*		Flowers			Infusion			Against cough	
		Flowers and leaves			Infusion			Pulmonary problems	
			Leaves		Fresh, revetment			Nail treatment, painful spots	
						Infusion	Bronchitis	Respiratory problems, asthma, cough, pulmonal ailments, digestion regulation,	
					Syrup			Against cough, immune system strengthening	
*Trifolium pratense*		Aerial parts			Infusion			Hormonal stabilizer, thyroid gland hormones regulation, female hormones regulation	
		Flowers			Infusion			Against cancer, gynecological problems, hormonal stabilizer, regulation of female and thyroid hormones	
*Urtica dioica*^♦^	Whole plant^#^			Infusion			Blood cleansing, nervousness reducing		
			Aerial parts			Infusion	Iron deficiency	Anemia, complete blood count improvement	Immune system strengthening
			Apical parts			Infusion	Anemia, physical weakness, iron source		Immune system strengthening, iron deficiency
		Apical parts and leaves			Infusion			Anemia, strengthening immunity	
			Seeds^#^			Mixed with honey	Anemia	Strengthening immunity, hemoglobin increasing, *medical panacea*	
			Leaves	Fresh, revetment			Pain relief		
					Powder			Wounds	
						Infusion	Iron deficiency	Strengthening immunity, circulation improvement, mood improvement, calming effects, pain in chest, blood strengthening, stomach problems, skin diseases, detoxification, complete blood count improvement, diuretic, urinary ducts ailments, kidney diseases	Anemia
			Roots^#^			Infusion		Prostate ailments, strengthening immunity, blood cleansing, female reproductive system cancer	Anemia
					Bath			Circulation improvement	
				Tincture			Anemia		
*Vaccinium vitis-idaea*		Fruits			Mixed with honey			Immune system strengthening	
		Leaves			Infusion			Urinary tract and kidney ailments, against bacteria and *Escherichia coli*, regulation of uric acid in blood, kidney and urinary ducts inflammation, urinary ducts cleansing	
*Valeriana officinalis*^♦^		Roots			Cold maceration			Calming effects, depression, tachycardia, headache, treatment of neuroses	
					Tincture			Against insomnia, calming effects	
*Verbascum thapsus*^♦^	Whole plant^#^			Decoction			Against skin warts		
*Verbena officinalis♦*		Aerial parts			Infusion			Headache, as antibiotic, hormonal imbalance, kidney cleansing, bronchitis, liver treatment, bladder limescale elimination	
*Veronica officinalis*▲		Aerial parts			Infusion			Immune system strengthening, blood vessels cleansing▲, sleep improvement, calming effects	
*Viola odorata*	Flowers and leaves			Infusion			Heart disorders		
Xanthium spinosum*	Aerial parts			Decoction			Against diarrhea		
Xeranthemum cylindraceum*	Aerial parts						Soaked with warm water and applied as a revetment on the back against fever		

* First time mentioned usage in Serbia; ♦ Plants are included in European Pharmacopoeia 8.0; # Plant parts are different from those cited in European Pharmacopoeia 8.0; ▲ New usage of well known traditional plants

**Table 5:** Human and domestic animal nutrition and veterinary medicine use of plant species of the Aleksinac and Bor districts

Scientific name	Part of the plant			Type of preparation		Human nutrition			Veterinary purposes			Animal nutritionSS		
	Aleksinac	Bor	Aleksinac/Bor	Aleksinac	Bor	Aleksinac/Bor	Aleksinac	Bor	Aleksinac/Bor	Aleksinac	Bor	Aleksinac	Bor	Aleksinac/Bor
*Achillea millefolium*		Leaves			Fresh			As spice						
*Agrimonia eupatoria*	Whole plant			Fresh						Against udder inflammation in cows (mastitis)				
	Aerial parts			Fresh						Against udder inflammation in cows (mastitis)				
	Fruits			Fresh						Against udder inflammation in cows				
*Allium ursinum*	Leaves			Fresh			As salad							
				Dry			As spice							
*Asarum europaeum*		Leaves						Spice for cooked beans						
*Calendula officinalis*		Flowers			Fresh			As salad						
*Cichorium intybus*	Whole plant			Decoction						Against diarrhea in pigs				
	Roots			Decoction						Against diarrhea in domestic animals				
*Cornus mas*			Fruits			Fresh			As fruit					
						Processed		Juice, jam, Serbian delicacy “slatko,” marmalade, for cookies	Pekmez					
				Tincture			Liqueur							
*Corylus colurna*		Fruits			Fresh			As food						
*Crataegus monogyna*	Fruits			Fresh			Used in diet							
*Cydonia oblonga*	Fruits			Processed			Serbian delicacy "slatko”							
	Leaves			Decoction						Against diarrhea in domestic animals				
*Cynodon dactylon*	Aerial parts			Fresh								Food for domestic animals		
*Equisetum arvense*		Aerial parts			Infusion						Against diseases in pigs			
		Shoots			Infusion						Diseases in piglets			
*Fragaria vesca*		Fruits			Fresh			As fruit						
					Processed			Serbian delicacy “slatko,” juice, for ice cream, jam						
*Helleborus odorus*		Whole plant			Fresh, revetment						Swollen udder in sheep			
	Roots			Fresh						"natravuvanje"				
				Dry						"natravuvanje"				
*Juglans regia*	Fruits						Used in diet							
Lotus corniculatus*		Aerial parts											Meadow hay, livestock nutrition, used as food for domestic animals	
		Whole plant											For domestic animals as food	
*Lysimachia nummularia*		Leaves			Fresh								Livestock nutrition	
*Lythrum salicaria*	Flowering aerial parts			Infusion						Against diarrhea in pigs				
*Malus sylvestris*			Fruits		Fresh			Used in diet as fruit						
						Processed			Homemade vinegar					
					Dry			Used in diet as dried fruit						
*Melissa officinalis*			Leaves			Processed			Juice					
					Fresh			As spice for broth						
*Origanum vulgare*		Aerial parts						As spice						
*Physalis alkekengi*	Fruits			Fresh			As fruit							
				Processed			Serbian delicacy “slatko”							
*Plantago lanceolata*	Leaves			Fresh			As salad							
*Plantago major*			Leaves			Fresh			As salad					
					Fresh, revetment						Open wounds (dogs and cats)			
*Polygonum aviculare*			Leaves			Fresh							As food for canary	
												Used in animal diet		
*Prunella vulgaris*	Flowering apical parts			Infusion						Cow eyes treatment by flushing				
*Prunus spinosa*			Fruits			Fresh	Food supplement in crystallized honey		As fruit					
					Processed			Jam, juice, compote						
*Pyrus pyraster*		Fruits			Fresh			As fruit						
					Dry			As dried fruit						
*Quercus cerris*		Fruits			Dry			Flour, coffee						
*Robinia pseudoacacia*▲		Flowers			Fresh			Fresh flowers are nibbled						
					Processed			Juice, Serbian delicacy "slatko", honey, as food (breaded) ▲						
*Rosa canina*			Fruits		Fresh			As fruit						
						Processed		Juice, wine, pekmez	Jam					
		Petals			Processed			Serbian delicacy "slatko"						
Rubus plicatus*		Fruits			Fresh			As fruit						
					Processed			Serbian delicacy"slatko", juice, jam, for cookies, wine, marmalade						
*Rubus ulmifolius*	Fruits			Processed			Juice, Serbian delicacy "slatko"							
*Rumex acetosa*		Leaves			Fresh			As salad, fresh leaves are nibbled						
					Processed			Broth, salt pie, cooked dish						
*Rumex crispus*	Whole plant			Decoction						Against diarrhea in domestic animals				
*Rumex patientia*		Seeds			Infusion						Against diarrhea (pigs and cows)			
		Leaves			Processed			Salt pie, broth, fried dish						
					Fresh								Used in animal diet	
*Sambucus nigra*			Fruits	Fresh			Used in diet							
						Processed	Compot	Jam	Serbian delicacy "slatko"					
			Flowers			Processed			Juice					
*Satureja subspicata*		Aerial parts						As spice						
*Sempervivum tectorum*		Leaves			Fresh			Fresh leaves are nibbled						
*Sorbus aucuparia*		Fruits			Fresh			As food						
					Processed			Wine, marmalade, rakija						
					Dry			As dried fruit						
*Taraxacum* sect. *Ruderalia*			Flowers			Processed		Serbian delicacy "slatko", breaded as food	Dandelion "honey"					
		Peduncle			Fresh			Used in diet						
		Peduncle and leaves			Fresh			As salad						
			Leaves			Fresh			As salad					
*Thymus serpyllum*		Whole plant			Dry			As spice						
		Aerial parts			Fresh			As spice for chicken and turkey meat						
		Flowers						As spice						
*Tilia platyphyllos*		Trunk			Infusion						Against diarrhea in livestock			
		Flowers			Processed			Juice						
		Leaves			Fresh			Fresh leaves are used for traditional Serbian food sarma						
*Trifolium pratense*		Flowers			Fresh			Fresh flowers are nibbled						
*Trifolium repens*		Flowers			Fresh			Fresh flowers are nibbled						
		Whole plant											As food for domestic animals	
		Aerial parts			Fresh								Animal diet	
					Dried								Animal diet	
*Urtica dioica*			Aerial parts			Fresh						Food for turkeys		Food for pigs
					Processed			Juice, broth, salt pie						
			Apical parts	Fresh			Salad, rolled in small balls and swallowed, fresh leaves are added to homemade juices to enrich them with iron							
						Processed		Fried dish, salt pie, broth, cooked dish	Juice					
		Apical parts and leaves			Processed			Fried dish						
			Leaves			Processed		Fried dish, cooked dish, salt pie, juice, for bread	Broth					
								As spice						
*Vaccinium vitis-idaea*		Fruits			Fresh			Used in diet as fruit						
					Processed			Liqueur						
*Verbascum thapsus*	Whole plant			Decoction						Against warts on cows' udder				
*Verbena officinalis*▲	Aerial parts			Fresh						Udder inflammation in cows▲				
Vicia cracca*		Whole plant			Fresh								Used in animal diet	
		Aerial parts			Dry								Used as food for domestic animals, meadow hay	
Xanthium spinosum*	Aerial parts			Decoction						Against diarrhea (pigs)				

* First time mentioned usage in Serbia; ▲ New usage of well known traditional plants

**Table 6:** Folk and religious rituals and ethnoculture, and other purposes of plant species of the Aleksinac and Bor districts

Scientific name	Part of the plant			Type of preparation			Beliefs and contemplation		Other purposes		
	Aleksinac	Bor	Aleksinac/Bor	Aleksinac	Bor	Aleksinac/Bor	Aleksinac	Bor	Aleksinac	Bor	Aleksinac/Bor
*Achillea millefolium*		Stem						Used for *I Ching*			
		Flowers						Used for making garlands on the Fest day dedicate to St. John (celebrated on July 7)			
*Asarum europaeum*		Leaves						Protection against evil forces. Leaves are used as home and children protector			
	Roots			Maceration					Fragrant bath		
*Asparagus officinalis*		Aerial parts								Ornamental, used for making bouquets	
*Calendula officinalis*		Aerial parts			Oil extract					Tanning oil	
	Flowers			Infusion					Skin complexion improvement		
*Chelidonium majus*		Whole plant			Decoction					Egg dyeing	
*Cichorium intybus*		Aerial parts						Guardian of travelers			
			Roots			Powder					Substitute for coffee
*Clematis vitalba*	Branches								Handmade beehive (*“trmka"*)		
*Cornus mas*			Twigs				Gate decoration for St. George's Day (celebrated on May 6)	Gate decoration for Sfințișori (celebrated on March 22)			
		Flowers						Fresh flowers are used for custom for Easter			
*Corylus colurna*		Branches						The stick is used as protection against snakebite, gate decoration with handmade cross made with branches			
		Fruits						Fresh fruits are talismans			
*Dipsacus laciniatus*		Aerial parts						Home protection against evil forces			
*Galium aparine*	Whole plant						Used for custom for St. George's Day (celebrated on May 6)				
*Galium verum*		Aerial parts						Against spells, used for making garlands on the Fest day dedicate to St. John (celebrated on July 7)			
*Geranium macrorrhizum*		Whole plant								Ornamental plant	
		Flowers						Used on the Fest day dedicate to St. George (celebrated on May 6)			
		Flowers and leaves						Used for making garlands on the Fest day dedicate to St. George (celebrated on May 6)			
*Helleborus odorus*		Aerial parts						Protection against evil forces used fresh on the Fest day dedicate to St. George (celebrated on May 6)			
		Flowers						Gate decoration for St. George's Day (celebrated on May 6)			
	Leaves						Gate decoration for St. George's Day (celebrated on May 6)				
*Humulus lupulus*		Fruits								Stuffing pillows for better sleep	
*Hypericum perforatum*		Aerial parts						Used for making garlands on the Fest day dedicate to St. John (celebrated on July 7)			
			Flowers		Infusion			Tea made of flowers is drunk against evil spirits			
				Oil extract					As a cosmetic aid, face mask		
Laserpitium latifolium*		Roots						Dry roots are used for protection from evil forces and spells (home and personality protection)			
		Whole plant						Whole fresh plant is used as home protector against evil forces			
*Melilotus officinalis*		Aerial parts						Protection against evil forces			
*Melissa officinalis*			Leaves			Fresh					Fresh leaves are used for bee swarming
*Peucedanum longifolium*		Whole plant								Ornamental plant	
*Pinus nigra* J. F		Trunk								Resin source	
*Quercus cerris*		Branches						Branches with leaves is used for orthodox Christmas as ceremonial tree			
		Trunk								Firewood	
*Rosa canina*		Seeds			Balm					Cosmetic product for face care routine	
		Petals			Micellar water					Cosmetic product for face care routine	
*Salix alba*		Twigs						Young twig is placed around the waist to prevent back pain for whole year at St. George's Day, twigs with leaves are used to make garlands for St. George's Day (celebrated on May 6). Fresh twigs are used for gate decoration for Lazarus Saturday		Homemade baskets from twigs without leaves	
*Salix purpurea*	Twigs						Gate decoration for St. George's Day (celebrated on May 6)				
*Sambucus ebulus*	Fruits								Fruits were recalled for ink making		
*Sempervivum tectorum*		Whole plant						Grown in the garden as home protection			
*Tanacetum vulgare*		Aerial parts								For bouquets, ornamental plant	
		Flowers						Fresh flowers are used for making garlands on the Fest day dedicate to St. John (celebrated on July 7), decorative			
					Decoction			Protection against evil forces by sprinkling			
*Taraxacum* sect. *Ruderalia*		Flowers			Decoction					Egg dyeing	
*Teucrium chamaedrys*		Flowers and leaves			Infusion					Hair washing	
*Tilia platyphyllos*▲	Flowers			Decoction					Egg dyeing▲		
*Trifolium pratense*		Leaves								Fresh leaf is used for making patterns when eggs are dyed for Orthodox Easter	
*Urtica dioica*▲			Whole plant			Cold maceration			Vegetable plants and flowers watering	Spraying vegetables, insecticide	
						Decoction			Strengthening hair root	Egg dyeing	
			Aerial parts		Decoction					Egg dyeing, strengthening hair root	
					Infusion					Spraying peppers and tomatoes	
						Maceration			Mixed with garlic is used as a pesticide	Spraying vegetables, insecticide	
			Leaves	Fresh, revetment					Rubbing ear before piercing▲		
					Decoction					Strengthening hair root	
			Roots			Decoction				Against hair loss, hair washing, head rinse	Strengthening hair roots
Xeranthemum cylindraceum*	Aerial parts								Homemade broom		

*First time mentioned usage in Serbia; ▲ New usage of well known traditional plants

### Quantitative ethnobotanical analysis

The results of the study (Tables 4, 5 and 6) provide information on the use of 114 wild and few domesticated (but still wild growing) plant species quoted by respondents from East Serbia. Recorded plants belong to 97 genera and 47 families, of which the Asteraceae (14.0%), Rosaceae (13.2%), Lamiaceae (7.9%) and Fabaceae (7.9%) were the most represented, similarly to other ethnobotanical studies conducted in Serbia and the Balkans [12, 17, 21].

### Use-reports

Out of the total of 2333 reports on the use of plants obtained by respondents, 1653 reports were given for medical purposes, 496 for human nutrition, 26 for animal nutrition, 38 for veterinary purposes, 57 for folk and religious rituals and ethnoculture and 63 for other purposes. Out of a total of 155 informants, 113 women gave 1834 use-reports, while 42 men gave 499. There were no differences between statements provided by men and women with exception for notes on the herbal micellar water and herbal medicinal syrup which were stated specifically by women. There were no differences between men and women regarding the curative and prophylactic use of plants for certain illnesses and disorders. In regard to traditional knowledge on the use of the wild plants for nutrition, religious and other traditional customs and for other purposes, both genders gave more–less similar information. Statements related to ethnoveterinary purposes are more frequently given by men than women.

Out of 2333 use-reports, 1180 (50.6%) were provided by inhabitants from the cities, while 1153 (49.4%) were provided by village inhabitants. There were no statistically significant differences between data on the traditional use of plants between inhabitants from cities and from villages concerning plant part in use and disorders treated by herbs. This is mainly due to the fact that inhabitants of these small semi-urban areas are usually tightly stuck to surrounding rural places, still performing some farming or horticulture for their own needs. However, there were some differences related to the traditional use of plants. Much more statements on animal nutrition, veterinary purposes, folk and religious rituals and ethnoculture, as well as for some less frequent uses, were obtained from inhabitants settled in the villages. Utilization of plant species for certain handicraft uses, as well as the note on common chicory (*Cichorium intybus* L.) as a coffee substitute, was mentioned only by respondents settled in the villages.

### Frequency of citation (FC), relative frequency of citation (RFC) and relative importance index (RI)

In the present study, FC values ranged from 0.05 to 13.1 (Additional file 1: Tables 3 and 4). The highest FC values are recorded for *Hypericum perforatum* (13.1), followed by *Urtica dioica* (9.0) and *Plantago major* (5.1). RFC values ranged from 0.001 to 0.2 (Additional file 1: Tables 3 and 4). The highest RFC was recorded for *Hypericum perforatum* (0.2) and *Urtica dioica* (0.2) followed by *Plantago major* (0.1). As can be seen, the ethnomedicinal plants having high RFC values indicated their abundant use and widespread knowledge among the local communities. RI values ranged from 0.1 to 1 (Additional file 1: Tables 3 and 4). The highest RI values were calculated for *Urtica dioica* (1.0), followed by *Hypericum perforatum* (0.7) and *Rosa canina* (0.7).

All these plants are among the most frequently reported in several neighboring regions, i.e., studied sites from southeast and south Serbia and from Kosovo [12, 17–19, 21].

### Informant consensus factor

The documented uses of plants in folk medicine refer to the treatment of 15 different groups of disorders. The ICF values ranged from 0.0 to 100.0% and 36.4% to 88.5% for Aleksinac and Bor districts, respectively. The highest ICF value found for the Aleksinac district was related to endocrine system disorders followed by skin-related disorders (79.7%) and circulatory system disorders (69.4%), while the lowest ICF value was found for antiseptic activity and metabolic disorders (0.0%). On the other hand, the highest ICF value for the Bor district was determined for skin disorders (88.5%), followed by digestive system disorders (83.5%) and respiratory system disorders (81.4%), while the lowest ICF value was 36.4% for antiseptic activity (Additional file 1: Table 5). A large number of species described by respondents of Bor district are used for the prevention and healing of digestive and respiratory system disorders. Such a fact might be related to very expressed air and soil pollution, as a consequence of mining and severe dust emission [49]. For the two studied regions considered together, the ICF values ranged from 33.3% to 88.9%. The highest ICF value was determined for skin disorders, followed by respiratory system disorders (82.1%) and digestive system disorders (82.1%), while the lowest ICF value was found for reproductive system disorders (51.4%) and antiseptic activity (33.3%) (Additional file 1: Table 6).

The ICF values ranged from 0.0% to 100.0% and 0.0% to 100.0% for men and women in the Aleksinac district, respectively. The highest ICF value found for men was related to endocrine system disorders followed by skin-related disorders (71.4%) and circulatory system disorders (50.0%), while the lowest was found for metabolic disorders, musculoskeletal system disorders, sensory system disorders, tumor ailments and urinary system disorders (0.0%). On the other hand, the highest ICF value for women was determined for endocrine system disorders and tumor ailments, followed by skin-related disorders (78.4%), while the lowest was recorded for musculoskeletal and sensory system disorders (0.0%) (Additional file 1: Table 7). When it comes to Bor district, the ICF values ranged from 0.0 to 85.7% and 30.0% to 86.9% for men and women, respectively. The highest ICF value found for men was related to sensory system disorders followed by skin (77.8%) and immune system disorders (63.0%), while the lowest was recorded for metabolic disorders (0.0%). On the other hand, the highest ICF value for women was determined for skin system disorders and digestive system disorders (82.8%) followed by immune system disorders (81.5%), while the lowest was recorded for antiseptic activity (Additional file 1: Table 8). In both districts together, the ICF values ranged from 0.0 to 100.0% and 27.3 to 88.0% for men and women, respectively. The highest ICF value found for men was related to endocrine system disorders followed by skin system disorders (79.6%) and immune system disorders (69.4%), while the lowest was recorded for metabolic disorders (0.0%). On the other hand, the highest ICF value for women was determined for skin-related disorders, followed by immune system disorders (82.8%) and respiratory system disorders (80.1%), while the lowest was recorded for antiseptic activity (27.3%) (Additional file 1: Table 9).

Regarding differences recorded in the city and surrounding villages, it was shown that the ICF values ranged from 0.0 to 100.0% in Aleksinac district. The highest ICF value found for both, citizens from the town and citizens from surrounding villages, was related to endocrine system disorders followed by skin system disorders (66.7% and 80.4%, respectively), while the lowest was recorded for musculoskeletal, reproductive, respiratory, sensory, urinary system disorders and musculoskeletal system disorders, respectively (Additional file 1: Table 10). When it comes to Bor district the ICF values ranged from 29.2 to 80.0% and 0.0 to 87.5% for citizens from the town and citizens from surrounding villages, respectively. The highest ICF value found for citizens from town was related to skin system disorders followed by digestive system disorders (74.4%), while the lowest was recorded for general health (29.2%). On the other hand, the highest ICF value for citizens from surrounding villages was determined for sensory system disorders, followed by skin (82.4%) and respiratory system disorders (77.3%), while the lowest was recorded for metabolic disorders (0.0%) (Additional file 1: Table 11). In both districts, the ICF values ranged from 28.0 to 82.5% and 0.0 to 100.0% for citizens from the town and citizens from surrounding villages, respectively. The highest ICF value found for citizens from town was related to skin system disorders followed by digestive (75.1%) and respiratory system disorders (74.8%), while the lowest was recorded for general health (28.0%). On the other hand, the highest ICF value for citizens from surrounding villages was determined for endocrine system disorders followed by skin (86.8%) and immune system disorders (79.2%), while the lowest was recorded for antiseptic activity (0.0%) (Additional file 1: Table 12).

### Use value (UV)

In the present study, the UV (Additional file 1: Table 13) in Aleksinac district ranged between 0.02 and 0.8. Based on UV data, the five most commonly used ethnomedicinal plant species were *Hypericum perforatum* (0.8), *Urtica dioica* (0.6), *Plantago major* (0.3), *Sambucus nigra* (0.3) and *Achillea millefolium* (0.3). The UV (Additional file 1: Table 14) in Bor district ranged between 0.01 and 1.4. The five most commonly used ethnomedicinal plant species in Bor district were *Urtica dioica* (1.4), *Hypericum perforatum* (1.3), *Sambucus nigra* (1.1), *Rosa canina* (0.9) and *Rubus plicatus* (0.7). These species were used for diverse purposes which are indicated in Tables 4, 5 and 6.

### Fidelity level

Fidelity level (FL) value in Aleksinac district ranged from 21 to 100%. The highest FL of 100% was recorded for *Allium ursinum* and *Crataegus monogyna* (circulatory system disorders), *Althaea officinalis* and *Hedera helix* (respiratory system disorders) and *Betula pendula* (urinary system disorders) (Additional file 1: Table 15). Further results showed that FL value in Bor district ranged from 22 to 100%. The highest FL of 100% was recorded for *Alchemilla vulgaris* (reproductive system disorders)*, Melilotus albus* (circulatory system disorders) and *Rumex patientia* (digestive system disorders) (Additional file 1: Table 16). In addition, the results for the entire studied area showed that FL values range from 25 to 100%. The highest FL of 100% was recorded for *Alchemilla vulgaris* (reproductive system disorders), *Crataegus monogyna* and *Melilotus albus* (circulatory system disorders) and *Rumex patientia* (digestive system disorders) (Additional file 1: Table 17). FL values indicate that respondents from Aleksinac district mostly use plants for skin system disorders (five species), while respondents from Bor district mostly use plants typical for the treatment of digestive system disorders (13 species).

Based on use-records given by men and women, FL values, in Aleksinac district, ranged from 75 to 100% and 33.3 to 100.0%, respectively. The highest FL of 100% according to men uses was recorded for *Juglans regia* (endocrine system disorders) (Additional file 1: Table 18), while for women was recorded for *Betula pendula* (urinary system disorders), *Crataegus monogyna* (circulatory system disorders) and *Hedera helix* (respiratory system disorders) (Additional file 1: Table 19). On the other hand, FL values in Bor district ranged from 30.8 to 88.9% (based on man uses) and 20.0 to 100.0% (based on women uses). The highest FL according to men uses was recorded for *Melissa officinalis* (nervous system disorders) (Additional file 1: Table 20), while for women was recorded for *Alchemilla vulgaris* (reproductive system disorders), *Epilobium parviflorum* (urinary system disorders), *Euphrasia officinalis* (sensory system disorders), *Melilotus albus* (circulatory system disorders) and *Mentha longifolia* and *Rumex patientia* (digestive system disorders) (Additional file 1: Table 21). In addition, the results for the entire studied area showed that FL value ranged from 27.8% to 100% (man uses) and 20.0% to 100% (women uses). The highest FL based on men use-records was recorded for *Juglans regia* (endocrine system disorders), *Pulmonaria officinalis* (respiratory system disorders) and *Sambucus nigra* (immune system disorders) (Additional file 1: Table 22). The highest FL based on women use-records was recorded for *Alchemilla vulgaris* (reproductive system disorders), *Betula pendula* and *Epilobium parviflorum* (urinary system disorders), *Crataegus monogyna* and *Melilotus albus* (circulatory system disorders), *Euphrasia officinalis* (sensory system disorders) and *Mentha longifolia* and *Rumex patientia* (digestive system disorders) (Additional file 1: Table 23). FL values indicate that men from Aleksinac district mostly use plants for skin system disorders (two species), while men from Bor district mostly use plants typical for the treatment of digestive system disorders (five species). FL values indicate that women from Aleksinac district mostly use plants for skin system disorders (five species), while women from Bor district mostly use plants typical for the treatment of digestive system disorders (12 species).

In addition, based on use-records given by citizens from the town and citizens from surrounding villages FL values in Aleksinac district ranged from 55.6 to 100% and 42.9 to 100.0%, respectively. The highest FL (citizens from town) was recorded for *Paliurus spina-christi* (digestive system disorders) (Additional file 1: Table 24), while the highest FL (citizens from surrounding villages) was recorded for *Althaea officinalis* (respiratory system disorders), *Betula pendula* (urinary system disorders), *Crataegus monogyna* (circulatory system disorders), *Hedera helix* and *Pinus nigra* (respiratory system disorders) (Additional file 1: Table 25). On the other hand, FL values in Bor district ranged from 50.0% to 100.0% (citizens from town) and 18.2 to 100.0% (citizens from surrounding villages). The highest FL (citizens from town) was recorded for *Cichorium intybus* (digestive system disorders) and *Sambucus nigra* (respiratory system disorders) (Additional file 1: Table 26), while the highest FL (citizens from surrounding villages) was recorded for *Alchemilla vulgaris* (reproductive system disorders), *Epilobium parviflorum* (urinary system disorders), *Euphrasia officinalis* (sensory system disorders), *Humulus lupulus* (nervous system disorders), *Melilotus albus* (circulatory system disorders) and *Mentha longifolia* and *Rumex patientia* (digestive system disorders) (Additional file 1: Table 27). In addition, the results for the entire studied area showed that FL values ranged from 23.1% to 100% (citizens from town) and 25.0% to 100% (citizens from surrounding villages). The highest FL (citizens from town) was recorded for *Alchemilla vulgaris* (reproductive system disorders), *Euphrasia officinalis* (sensory system disorders), *Melilotus albus* (circulatory system disorders), *Paliurus spina-christi* and *Rumex patientia* (digestive system disorders) and *Valeriana officinalis* (nervous system disorders) (Additional file 1: Table 28), while the highest FL (citizens from surrounding villages) was recorded for *Althaea officinalis* (respiratory system disorders), *Betula pendula* (urinary system disorders), *Crataegus monogyna* (circulatory system disorders), *Pinus nigra* and *Pulmonaria officinalis* (respiratory system disorders) and *Rumex patientia* (digestive system disorders) (Additional file 1: Supplementary Table 29).

FL values indicate that citizens from Aleksinac town mostly use plants for skin system disorders (two species), while citizens from Bor town mostly use plants typical for the treatment of immune system disorders (three species). FL values indicate that citizens from surrounding villages in Aleksinac district mostly use plants for skin system disorders (four species) and respiratory system disorders (four species), while citizens from surrounding villages in Bor district mostly use plants typical for the treatment of digestive system disorders (14 species).

Obtained results point to the fact that although citizens from these districts rely on the official health care system, still medicinal plants have significant value in everyday life for these people.

### Multivariate analysis

**Fig. 1:**
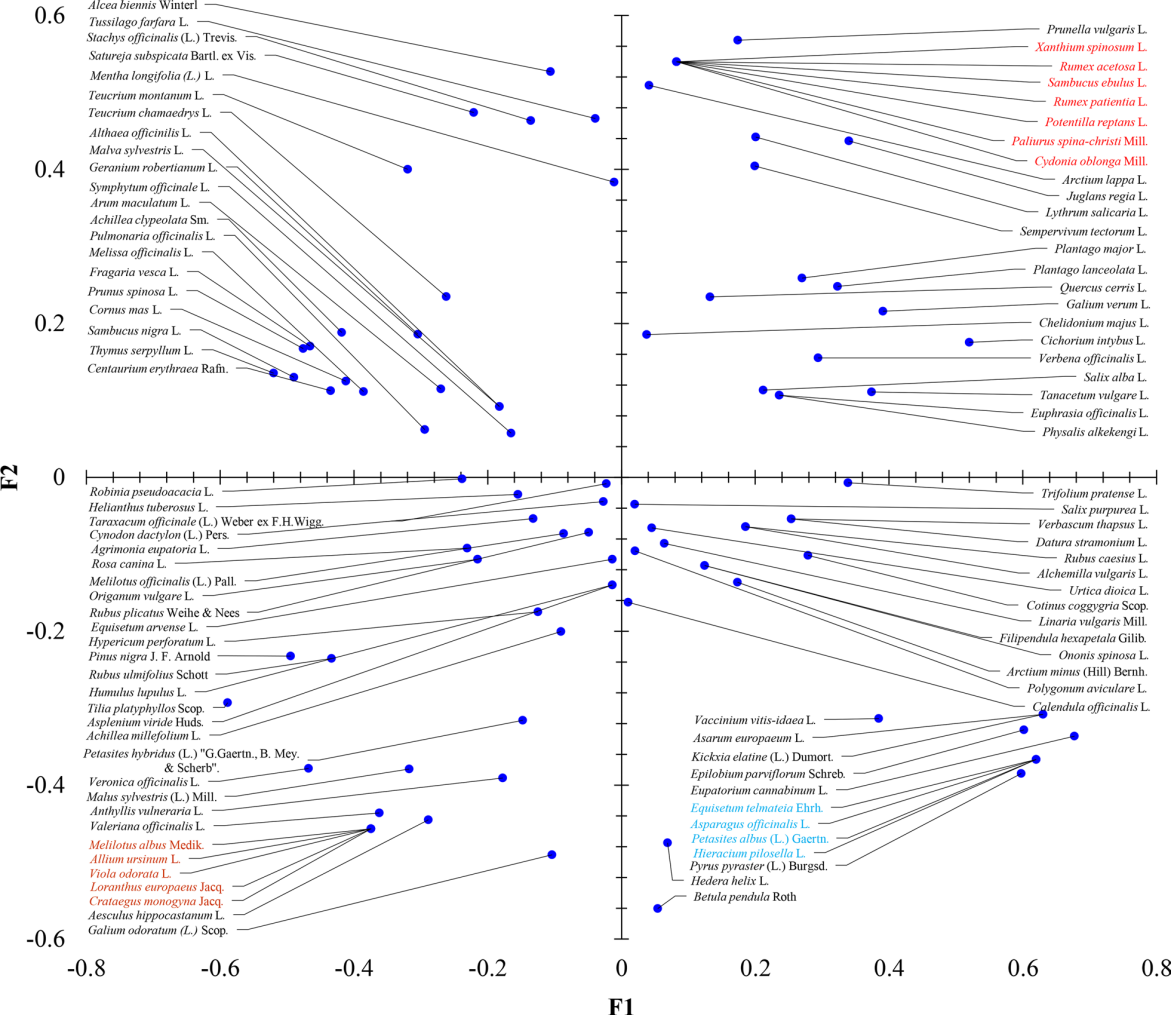
Principal coordinate analysis of plants used in traditional human medicine in surveyed area in East Serbia

A scatter plot from principal coordinate analysis (PCoA) showed the formation of three distinct homogeneous groups (Fig. 1). The first group consisted of seven species (*Rumex acetosa*, *Xanthium spinosum*, *Sambucus ebulus*, *Rumex patientia*, *Potentilla reptans*, *Paliurus spina-christi* and *Cydonia oblonga*). All these species are grouped in relation to one variable, i.e., the effects on digestive system disorders. The second group was formed from five species (*Melilotus albus*, *Loranthus europaeus*, *Allium ursinum*, *Crataegus monogyna* and *Viola odorata*) acting mainly for circulatory system disorders, according to information from respondents. The third group consisted of five species (*Petasites albus*, *Asparagus officinalis*, *Equisetum telmateia*, *Hieracium pilosella* and *Pyrus pyraster*) which were mentioned for urinary system disorders application.

### Traditional preparations and remedies

Out of 114 reported species, 101 (88.6%) are used for medical purposes (Table 4). Plants are the most commonly used to treat digestive system disorders (49.1%), circulatory system disorders (41.2%) and respiratory system disorders (35.1%). These findings on a wide use of plants for digestive and respiratory system conditions are in accordance with other ethnobotanical studies in Serbia [17, 18, 21]. Concerning plants used for medicinal purposes, 33 species are included in the European Pharmacopoeia 8.0. [50] (marked with ♦  in Table 4). Plant parts, i.e., plant drugs which differ from citations provided in European Pharmacopoeia 8.0. [50], are marked with hash (#) Table 4. Some interesting traditional remedies were recorded, which haven’t been so far indicated in previous ethnopharmacological studies in the Balkans.

*H. perforatum* is reported as an herbal remedy effective against every disorder (medical panacea) which is in agreement with some earlier investigation. Although spatially distant, in the Arribes del Duero (Spain), the cultural importance of *H. perforatum* oil is unquestionable. It is also cited, literally translated, that “What *Hypericum* doesn’t cure the doctor certainly won’t” [51]. Many previous studies on the Balkans documented its usage against diseases of different organ systems [52–55].

It is claimed that the best herbal medicines, with the most healing properties, are made if this plant is collected on July 7 (St. John’s Day). It is even stated that flowers smell different on that day. Flowers are collected, basted with sunflower or olive oil, and left in the sunlight for 40 days. On the 40th day, oil extract is brought into the home. Similar methods for preparation are noted in earlier studies both on Serbian territory [12] and in the region [21, 56–58]. Oil extract is used externally for skin ailments, hemorrhoids and gynecological problems. Živković and colleagues (2020) reported the same type of preparation against skin complaints and hemorrhoids [19], while Varga and collaborators (2019) reported the usage of infusion against gynecological diseases [59]. Internally, it is applied for digestive ailments and improving general health conditions.

Informants are also knowledgeable on the toxicity of certain medicinal plants, as well as their side effects when combined with medicinal drugs. For example, they stated that *H. perforatum* tea should not be used together with drugs used in the treatment of malignant diseases because it can “completely reverse the effect of the drug.” Also, tea should not be drunk for more than seven days in a row.

When it comes to medicinal herbs, the so-called *Ciklus svetojovanskog bilja* (lit. Cycle of St. John’s herbs) is also mentioned. It encompasses the period from June 28 (*Vidovdan*; lit. St. Vitus day) to July 7 (*Ivanjdan*; St. John’s Day) when it is most desirable to collect certain medicinal herbs (*Achillea millefolium, Melissa officinalis* and *Hypericum perforatum*).

It is not unusual for some traditional receipts in Serbia to be prepared during the 40 days. It is possible that roots of the method of preparation lie down in the Christian religion where the number 40 has a particular meaning. Christians believe souls need 40 days after death to leave the Earth, in the Bible it is mentioned that the great flood lasted 40 days and nights [60], Moses spent 40 days and nights in Mount Sinai, the same number of days Jesus fasted and was tempted in the wilderness [61]. We assume that it is easier for people to remember and pass on the knowledge related to the preparation if there is some universally known fact.

### Traditional tinctures

Homemade tinctures are usually prepared with the fruit spirit (`*rakija`* in Serbian). Among the most used, there are the tinctures made of a single herb, such as wild thyme, nettle or marigold, or those prepared of mixture herbs.

Tincture for a massage, circulation improvement and thrombosis prevention is made of hundreds of marigold flowers (*Calendula officinalis*) put in a liter of *“rakija”*. The usage of this herb as a tincture also was noted in the research conducted in southwestern Serbia [14]. The comfrey (*Symphytum officinale*) tincture is made in a similar way (one mature root is put in one liter of rakija), or the drug is mixed with five wild chestnut fruits and one rosemary branch in half a liter of spirit for healing of leg pains and varicose veins. One ethnobotanical investigation previously published, conducted in the northeastern part of Croatia, reported the traditional use of comfrey’s roots as tincture against cardiovascular disorders [62].

### Syrups and honey

Few informants mentioned preparation of homemade syrup for strengthening immunity, which was especially recommended to children*.* The syrup is made from *Pinus nigra* shoots, *Plantago lanceolata* leaves, *Sambucus nigra* flowers, *Thymus serpyllum* aerial parts and *Tussilago farfara* leaves. The preparation is made by boiling of sugar (3 kg) for 10 min and thereafter adding 300 g of each plant drug to cook for an additional 9 min exactly. In the final step, 15 g of commercial citric acid is added, and the mixture is cooked for one more minute.

Nettle (*Urtica dioica*) syrup is considered as the best medicine for strengthening the immune system in patients with anemia. Firstly, red wine is cooked with yellow sugar. When it comes to boiling, 50 young apical parts of nettle are added, and it is cooked for 2–3 min. Every morning before breakfast, a small glass of preparation should be consumed.

To the best of our knowledge, these receipts are mentioned for the first time on the Balkan Peninsula.

The honey is often mixed with herbs for different treatments. For example, there is a recipe quoting that two tablespoons of the black pine (*Pinus nigra*) pollen, locally known as “flower powder” (in Serbian, “*cvetni prah”*) are mixed with 200–300 g of meadow honey. It is believed that this remedy improves respiratory ailments. These results are similar to those obtained from the Kopaonik mountain where buds and needles of *P. sylvestris* are also mixed with honey in the final part of the preparation of medicine against chronic bronchitis [12] Mustafa and colleagues (2012b, 2020) reported mixing cones of *Pinus* spp. with honey to treat respiratory system ailments, which is partially in agreement with the results of the current study [56, 63]. The arum is also mixed with honey: one kilogram of *Arum maculatum* rhizome is combined with a kilogram of honey. The mixture is consumed 2–3 times a day before a meal for digestion and hemorrhoids treatment. A particular product is made of dandelion flowers (*Taraxacum officinale*). It is called “dandelion honey.” Dandelion flowers are collected and cooked together with water and sugar until the flowers are completely cooked in the mixture. This product is frequently prepared and consumed in the Balkans, according to some previous ethnobotanical studies [56, 64].

### Ointment for skin diseases

Ointment is prepared with a hundred of marigold petals fried with a tablespoon of homemade grease and it is used cold for the treatment of urticaria. Our results are congruent with some previous studies [16], which mentioned the similar use of these flowers against different skin injuries [12–14, 19]. Twigs of elder (*Sambucus nigra*) are used for preparing balm for burns therapy and insect and spider bites treatment. Twigs are grated and mixed with plant wax, honey, the incense and chopped yarrow (*Achillea millefolium*) leaves. The mixture is filtered and stored in a cold place.

### Decoction for urinary diseases

Aerial parts of *Equisetum telmateia* are mixed with young corn cobs and corn silk (elongated stigmas) and cooked in water. The mixture is cooked until the volume of water drops to a third of the initial volume, and the color becomes red. The decoction is used against urinary problems.

### Wild herbs for human nutrition

There were 37 (32.5%) plant species recorded for human nutrition (Table 5). Out of 37 species used for nourishment, 35 species are simultaneously used for medical treatments. The plant drugs are used fresh, dry and processed. Homemade food and beverage products made from or with the addition of wild plants include juices, jams, compotes, wine and Serbian traditional sweet dish “*slatko*” [65]. Fresh and dried herbs are often used for seasoning, i.e., as spices, either single or in mixtures (e.g., *Achillea millefolium, Allium ursinum, Melissa officinalis, Origanum vulgare, Satureja subspicata, Thymus serpyllum,* and *Urtica dioica*). Salad used for nutrition, additionally providing health benefits, especially for stomach ailments regulation is prepared as a mixture of leaves of five species: *Fragaria vesca*, *Plantago major*, *Rubus plicatus*, *Rumex acetosa* and *Urtica dioica*.

For the preparation of the sweet delicacy* “slatko”* the petals of the dog rose (*Rosa canina*) are used. Firstly, the petals are mixed with water and sugar and the mixture is boiled for at least half an hour. In the next step, a few drops of lemon juice are added to restore a petal color (should remain as gently reddish).

Traditional and beloved herbal beverages in Balkans mainly refer to those made of elder flowers, which has been already reported in some former studies [16]. The new information obtained in our research is related to the note about potential negative effects on men's fertility if it is overused. Elder fruits are used either fresh or processed, mostly for preparing a fruit wine. Fruits of elder are cooked for 15 min at 80 °C with water and sugar (half of the quantity of used fruits), and left for fermentation. Similar use of elder is already known in Europe [66].

Replacement for traditional coffee drink is made of dried and grinded chicory (*Cichorium intybus*) roots mixed with Turkey oak (*Quercus cerris*) acorn for nicer flavor.

### Overlap of medicinal and food plants

In our study, nearly 100% plant species overlap as food and medicine. This finding is in agreement with another study conducted in Negotin, the very near region [17]. In that study, all plants mentioned as food plants are also used in herbal medicine. An identical situation is observed in the southeastern Serbia (Suva Planina) [16]. On the contrary, on the Kopaonik Mountain (Central Serbia), out of 24 plant species mentioned for nutrition, slightly more than a half are used for both nutrition and medicine [12]. However, in the neighboring country (Croatia) results are different. In a study conducted in Dalmatia, 41 plant taxa are mentioned to be used exclusively for treating a variety of ailments, 43 exclusively as food and 42 for both purposes [59]. Also, in Istria, out of 121 species, 31 species are used exclusively as food, 50 as medicine, and 40 species overlap [67]. On the other hand, a study conducted in the areas of the towns of Našice and Djakovo showed that 37 species are used exclusively as medicine, and 7 species overlap, but there are no plants that are used only as food [62].

### Veterinary medicine

The 14 of 114 recorded plant taxa (12.3%) are used in veterinary medicine (Table 5). Plants are mostly used fresh, especially as revetment, and as extracts in form of infusion or decoction. A certain similarity in species used in human and veterinary folk medicine was noticed: of 14 species used in veterinary medicine, only two—the *Helleborus odorus* and *Rumex crispus*—are not used in humans.

Traditional practices for the treatment of domestic animals are preserved mostly in rural areas. The hellebore was indicated as an herbal remedy efficient in terminal illness in livestock and pigs. It is known that such practices were often used in the past, especially for horses [68]. Technique that relies on the usage of hellebore roots for this purpose is called in Serbian “natravuvanje stoke” (there is no suitable translation for the term specified). The cleansed fresh or dried part of the root is directly inserted in a certain part of the animal body: into the ears in the case of pigs or in loose skin below the neck in the case of cows. The function of drying is to prevent root bending for easier insertion. Ear of the sick pig is pierced by an awl and a cleansed root is inserted. The root is not removed until the surrounding area becomes purulent and swollen. Part of the ear falls off but a life-threatened animal survives. When cows are treated, a cleansed root is inserted in a dewlap and the tip of the root is left to jut. Root is left in a loose skin on the neck for 24 h. After the defined period has elapsed, root is removed and accumulated pus leaks out. The method is not approved by veterinarians today. However, the informants asserted that sometimes it is necessary—as an extreme way to save the sick animal. The utilization of hellebore for ethnoveterinary purposes is known from earlier ethnobotanical studies conducted in Serbia [12, 13, 17], but details are for the first time provided here.

Mastitis in cattle is caused by various factors, mainly infections, but also by some other physical and chemical traumas [69]. Two plant species are mentioned for mastitis treatment, the agrimony (*Agrimonia eupatoria*) and the vervain. Ethnoveterinarian study from Italy provides information on the usage of vervain against mastitis in cows although the methods of application differ [70].

### Folk and religious rituals and ethnoculture

The usage of a total of 17 (14.9%) plant species is linked to traditional customs and rites (Table 6). Many plant species are utilized for religious purposes and various stories and legends are related to their role in ethnoculture. There were some differences in the use of herbs in traditional customs between two investigated districts. Performing rituals for the Fest Days is more developed and practiced in the Bor District. Most of the respondents stressed that wild plants are gladly used in celebration of some holy and festive days, especially the St. George’s Day (“Đurđevdan” in Serbian; May 6) and the St. John’s Day (“Ivanjdan” in Serbian; July 7). In Bor district, more data were obtained on folk customs and tradition, possibly because of the need to protect the national identity in a multicultural community. In the Bor District, four plant species are used for St. George’s Day celebration. Early in the morning, *H. odorus* aerial parts are tied and left to hang in the part of the yard where cattle reside as protection against spells and impure forces. On the Eve of St. George’s Day, geranium (*Geranium macrorrhizum*) flowers and leaves and willow (*Salix alba*) twigs with leaves are combined to make garlands. Garlands are placed around buckets used for sheep milk collection to increase milk yield. The willow is also placed around the waist to avoid back pain throughout the year. Hazelnut (*Corylus colurna*) is also used for this Fest Day. It is also used in rural areas as a defense against snakes. Hazelnut is put on the fence around the house, especially in front of the front door because it is believed that snakes do not approach this area. Also, informants carry sticks made of hazelnut branches in the wild to protect them against snakes. Hazelnut fruit is an amulet. Usage of these species has been confirmed and found in the literature. It is also known that other species of the genera *Corylus* are used in the same way as mentioned hazelnut [68].

For Easter, local people make a mixture of *Cornus mas* flowers and *Urtica dioica* leaves. Red wine and an Easter egg (exclusively red color) with bread crumbs are added to the mixture. Early in the morning, in front of the front door, family members cross themself, turn in the direction of sunrise three times and after every turn, they drink a teaspoon of this beverage. The order is from the oldest to the youngest member of the family.

Garlands are made for St. John’s Day too. Different plant species are included but the most abundant one is yellow bedstraw (*Galium verum*). Other plant species that can be inserted are *A. millefolium*, *H. perforatum*, *Tanacetum vulgare*. Garlands are hung on the front door of home and kept until the next year on the same day. On that day, last year’s garlands are thrown away and the new ones are made. Custom is repeated every year. It is believed that this custom secures home protection from negative influence, evil thoughts and glances. It is shown that yellow bedstraw is a favorite flower among people and that this custom is used a lot. There is also evidence that garlic can be inserted into the garlands [68]. Tea made of *H. perforatum* is drunk as a part of religious beliefs. This plant is considered as one with an extremely magical effect for evil spirits expelling. Because of that, it is consumed only in the evening. The magical power of *H. perforatum* was also confirmed in the literature [71].

In the Aleksinac, customs for St. George’s Day are also performed. The customs differ from the ones in the Bor District. One of the symbolic acts for celebration is home gate decorating. Plant species used are *Cornus mas*, *Salix purpurea* and *Helleborus odorus*. Earlier, children were bathed with these plants and red eggs that have been kept since Easter to ensure their health. Young couples were collecting aerial parts of *Galium aparine* and binding themself around the waist because it is believed that done deed will secure their love forever. Beliefs related to uses of this plant are known from the literature. If the young woman takes part of the plant and inserts it into her left sock, she will be appealing to others and liked by them [68].

Oak (*Quercus cerris*) is used as a ritual tree. It is brought into the house and also left in front of the door on Christmas Eve.

People use *Asarum europaeum* in their diet as a spice and for religious purposes. It should be kept especially if there are small children. It is believed that they will not cry or be afraid in the presence of this plant. Occasionally, it is used to incense houses. *Sempervivum tectorum* is planted in the front of the house as a protection from evil forces.

*Laserpitium siler* is a species that is connected to various stories and beliefs, primarily “as a key for all locks,” the key to success, and as a protector of home and a person [8]. Thanks to such beliefs it is good to carry a part of this plant in a wallet or at home. Some informants mentioned that they have this plant in their car as a protection from accidents. Usually, root is used, but any other part of the plant can be used. Since the species is rare in nature, the broad-leaved laserwort (*L. latifolium*) is used as a replacement according to the results of the current study. Some informants mentioned that crumbled parts are burnt and used as smoked incense to protect home from spells and witchcraft. It is believed that hedgehogs can find the plant to protect their cubs. This story is characteristic of east Serbia [68].

According to respondents, the chicory is used as a guardian for travelers. It is believed that it always brings people back to the place where they came from. The plant is a good protector from diseases, accidents and other bad things.

### Other uses

There are few plant species listed for face and body care use. Herbal cosmetic products varied in their complexity. There were simple products that include material from the single species, with a simple method of preparation (e.g., St. John’s Wort oil extract). Some informants indicated that asarabacca (*Asarum europaeum*) was applied in fragrant baths in the past. Squashed roots are left in a bowl of water and poured into a bath. Today, it is not practiced anymore due to the awareness of respondents on the toxicity of the plant.

Homecrafts are pretty rare in the study area, but there are still some rural households using plants in some practices. For example, *Clematis vitalba* branches are used for making simple beehives, and *Melissa officinalis* for gathering bees together due to its pleasant scent. The nettle was recorded as a pest repellent which is in accordance with data provided by Mullalija and collaborators (2021) [21].

### Ethnobotanical richness and the similarities with other ethnobotanical investigations in Serbia (Jaccard index)

Results of this research were compared with data obtained from earlier studies conducted on the territory of Central Serbia [12], southwestern Serbia [13, 14], and especially of those performed in the closest areas, i.e., parts of the eastern and southeastern Serbia [15–20] (Table 7). According to the JI (Table 7), the highest degree of similarity was recorded with studies conducted on Suva Planina mountain (southeastern Serbia) with a JI of 28.7, Kopaonik (Central Serbia) with a JI of 27.3, as well as River Timok region and Mountain Svrljig region (eastern and southeastern Serbia) with a JI of 24.1. It was shown that small and isolated areas provide more specific information on the traditional uses of wild plants [72].

**Table 7:** Ethnobotanical comparison between our results and ethnobotanical data conducted in other investigated areas of Serbia

Area	Year(s) when the studies conducted	No. of plant taxa	No. of medicinal taxa	No. of taxa used in human nutrition	No. of taxa used in veterinary medicine	No. of taxa used in animal nutrition	No. of taxa used in beliefs and contemplation	No. of taxa used in other purposes	Plants that overlap from this study with plants from previous studies	Jaccard Index	References
Central Serbia	2002–2005	91	91	25	11	/	/	7	43	27.33	Jarić et al. 12
Southwestern Serbia	2010	62	62	5	3	/	/	8	22	15.79	Pieroni et al. 13
Southwestern Serbia	2011	69	69	3	/	/	/	/	30	22.00	Šavikin et al. 14
Eastern Serbia	2011–2012	45	45	/	/	/	/	/	24	20.45	Zlatković et al. 15
Southeastern Serbia	2012–2014	137	128	43	5	/	/	16	30	28.72	Jarić et al. 16
Eastern Serbia	2016	37	37	19	3	8	1	17	20	14.39	Janaćković et al. 17
Eastern and southeastern Serbia	2015–2017	195	190	/	/	/	21	4	53	24.10	Matejić et al.^18^
Southeastern Serbia	2015	85	/	/	/	/	/	/	28	17.75	Živković et al. 19
Eastern Serbia	2017	192	/	/	46	/	/	/	14	19.55	Marković et al.^20^
Eastern Serbia	2019	114	100	37	14	6	17	24	114	100.00	Present study

### Novel ethnobotanical records

The results of our study highlighted the new usage of some well-known traditional plants in Serbia and Balkans. These plant species are: *Robinia pseudoacacia*, *Sempervivum tectorum*, *Taraxacum officinale*, *Teucrium chamaedrys*, *Tilia platyphyllos*, *Urtica dioica*, *Verbena officinalis* and *Veronica officinalis.* They are used for different purposes: four for medicinal uses (*Sempervivum tectorum* against headache; *Taraxacum officinale* for face washing, positive effect on vocal cord, against jaundice; *Teucrium chamaedrys* for eye ailments, cataract treatment; *Veronica officinalis* for blood vessels cleansing), one for veterinary use (*Verbena officinalis* against udder inflammation in cows), one for nutrition (*Robinia pseudoacacia* breaded as food) and two for other purposes (*Tilia platyphyllos* for egg dyeing; *Urtica dioica* for rubbing ear before piercing) (Tables 4, 5 and 6▲).

Interesting information was obtained for the field horsetail, the *Equisetum arvense*. Although the use of this plant in Serbia and in the Balkans is well known from before, the usage of fertile parts was not mentioned in any of the other ethnobotanical studies. Herbal remedies made from fertile parts are applied in the same way as the sterile parts. The interesting note was on people’s perception of the herb. Usage of the fertile parts was recorded in the village Jakovlje, where women call the plant “*štukavac*,” because it occurs around St. George’s Day, after which it disappears (Serb. local folk dialect “*štukne* “ in Eng. fade away) until the next spring. According to the respondent, the fertile part (the spike) of the field horsetail was in fact considered as a different species.

After comparison with studies conducted in Serbia, in addition to the review of available textbooks on medicinal plants, we assumed that 11 species were noted for the first time (marked with asterisk (*) in Tables 4, 5 and 6). Of these 11 species, the application of 4 species—*Alcea biennis*, *Asplenium viride*, *Kickxia elatine* and *Xeranthemum cylindraceum*, in the folk medicine is novel information for the Balkan region (according comprehensive review of the most relevant ethnobotanical and ethnopharmacological surveys performed in the Balkan region [52,-54,56,57,59,62,63,73–88].

**Fig. 2:**
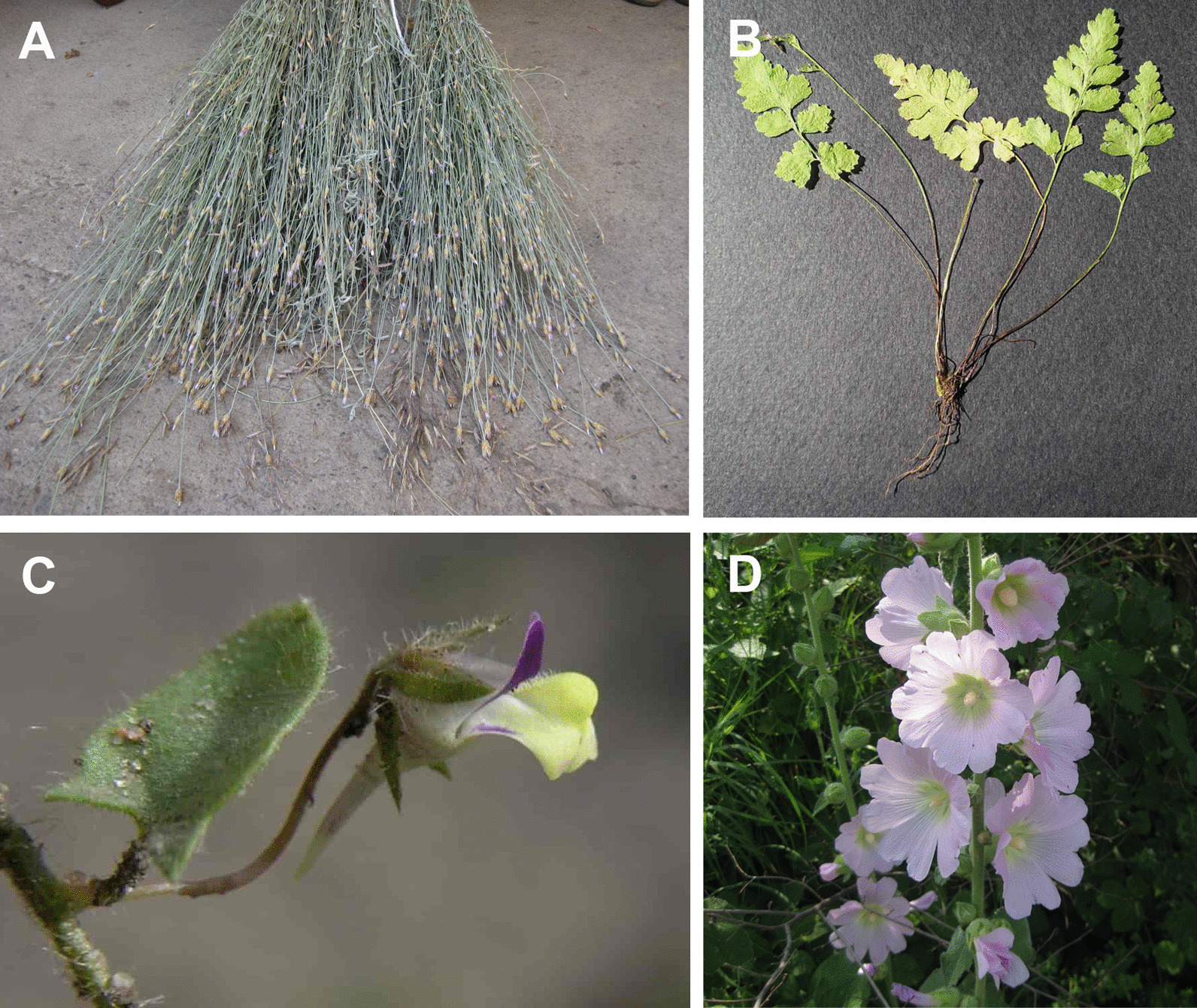
Novel information for Balkan region regarding usage of four species in the folk medicine. **A** *Xeranthemum cylindraceum* Sm. (Photograph by Miletić, M.); **B** *Asplenium viride* Huds. (Photograph by Janaćković, P.); **C** *Kickxia elatine* (L.) Dumort (Public domain); **D** *Alcea biennis* Winterl (Photograph by Miletić, M.)

In addition to use in some traditional handicrafts, the *Xeranthemum cylindraceum* (Fig. 2A) is also used for medicinal purposes. Brooms (aerial parts) are soaked with warm water and applied on the back against fever. There are several studies implying antipyretic activity of members of Asteraceae family [89–91]. The effect on health could also be attributed to the specialized metabolites of this species. The essential oil obtained from the aerial parts of the plant represents a terpenoid-rich mixture, with 1,8-cineole, α-terpineol, hexadecanoic acid and caryophyllene oxide as predominant compounds. The guaianolide-type sesquiterpene lactones xerantholide and 11,13-dihydroxerantholide were the major compounds found in the extracts, along with 3-hydroxybenzaldehyde. The sesquiterpene lactone of an eudesmanolide type 11,13-dihydroisoalantolactone and pseudoguaianolide confertin were present in extracts as well [92]. Sesquiterpene lactones, as well as essential oil compounds, exhibit an antipyretic activity [93, 94]. Therefore, the use in folk medicine as mentioned by respondents in our study sounds reasonable.

*Asplenium viride* (Fig. 2B) leaves are collected and prepared as an infusion. The purpose of the infusion administration is to cure fright. Potential medicinal properties of *A. viride*, as an antihypertensive drug, were noted in the research on cardiovascular diseases in the Iberian Peninsula and the Balearic Islands [95]. The phytochemical constituents of this plant species have not been sufficiently investigated. Plant with the same name in Serbian, the “strašnik” is *Ceterach officinarum* Willd. (syn. *Asplenium ceterach* L.). The etymology of the local name is linked to the “fear” (in Serbian “strah”), which could explain its use as an anxiolytic drug. *C. officinarum* is known from Serbian textbooks of medicinal plants [9, 96] and from ethnobotanical research [18].

According to our investigation, the *Kickxia elatine* (Fig. 2C) is used as a herbal remedy for wound healing, mainly for farmers being hurt during their work on the fields. In case of an injury, the plant is applied at the wounded place. There is a similarity in the use of the plant comparing our results with two studies conducted on spatially distant regions. Uses of *Kickxia elatine* are known from the Italian and Indian ethnobotanical studies. In Italy, it is directly applied to prevent and decrease the feet sweating [97]. The local tribes of the Western Ghats in India use this plant as a hemostatic agent and for wound treatments [98]. There are only a few studies focusing on the phytochemistry of this species. The main identified compounds are the iridoids, namely iridoid glycosides, kickxioside, antirrinoside, antirride, mussaenosidic acid, 5-O-menthiafoloylkickxioside and kickxin [99].

*Alcea biennis* (Fig. 2D) infusion is made of leaves. According to records obtained in our study, it is utilized for respiratory and digestive system disorders, i.e., for cough treatment, against sinusitis and against intestine diseases. The traditional uses of this species are recorded for Turkey and Iran [100–102]. There is much similarity in traditional use of the plant in folk medicine in our and these studies. To the best of our knowledge, *Alcea biennis* has not been studied from the phytochemical aspect.

### Concluding remarks

Both qualitative and quantitative methods are quite valid in ethnobotanical studies. While qualitative data collection allows in-depth exploration of traditional knowledge regarding wild plants, quantitative methods can be useful in the comparison of the efficacy of different data collection methodologies [103]. Quantitative analyses represent a tool for obtaining data comparable to other studies as well as deriving reasonable conclusions based on the data collected. Increasing quantification of ethnobotanical studies has been continuously highlighted by some ethnobotanists [104, 105]. Some authors evaluated the use of Ellenberg values to establish whether there are differences between the environmental preferences of wild medicinal and food plants. Similar quantitative analysis would strengthen the discipline and provide rigorous testing methods [67]. However, some limitations of our work may refer to revealing the group of plants most important to a culture of this area, since we used the quantitative methods to measure individual traditional botanical knowledge, but on the other hand, this is important for comparison, in the future, with ethnobotanical heritage in similar small regions. Also, we performed a multivariate analysis with a clearly defined goal in the first place, to find a connection between plants and their usage, keeping in mind Pieroni's (2002) observation that, in some cases, it is easier to be impressed with this method, but without motivation for its usage in the right way.

High migration rates, depopulation and aging are typical features for rural Balkan areas causing accelerated loss of ethnobotanical knowledge and traditional practices in agriculture. Local inhabitants typically acquire ethnobotanical knowledge from their ancestors (parents, grandparents) and older neighbors in direct contact, which is a medium- and long-term risk for permanent knowledge loss. In addition, informants were concerned about the threatening of ethnobotanical information through its oral transmission and general weak interest of the young.

Some authors implemented a participatory approach in ethnobotanical research [106] where involvement and active participation of the local inhabitants should be included in the decision-making process and sustainable management of plant resources. The necessary actions for the preservation of both ethnobotanical knowledge and resources of medicinal plants in the studied area could be summarized as: a) raising of public awareness on ethnobotanical knowledge and related culture heritage, b) promotion of ethnotourism and traditional herbal remedies, food and beverage products, c) organization of herbal tours and d) creation of a sustainable management plan for economically important plants.

Our study indicated that small and specific areas in the Balkans (rural, abandoned, economically devastated and with high migration rate) may be an important reservoir of ethnobotanical knowledge, providing new information on the traditional use of plants. Results emphasized the great importance of wild plants in the daily life of the natives. Indigenous plants are still significant in traditional medicine. Therefore, there is a necessity to preserve the traditional knowledge of plant use, especially in regard to potential relevance for further pharmacological surveys. New records on use of the wild plants, as well as the way of their processing and combination in traditional remedies and products, confirmed our starting hypothesis on unique features of the study area.

## Supplementary Information

**Additional fil 1**. Supplementary material.

## Data Availability

All the needed data collected for this study were analyzed and incorporated into this manuscript.

## Abbreviations

BEOU: Herbarium of the University of Belgrade—Faculty of Biology, Institute of Botany and Botanical Garden “Jevremovac”
FL: Fidelity level
FC: Frequency of citation
ICF-FIC: Informant consensus factor
ISE: The International Society of Ethnobiology
JI: Jaccard index
PCoA: Principal coordinate analysis
RCI: Relative cultural importance indices
RFC: Relative frequency of citation
RI: Relative importance index
UV: Use value
WHO: World Health Organization
