# Supplementary material for: Small regions as key sources of traditional knowledge: a quantitative ethnobotanical survey in the central Balkans
Source: J Ethnobiol Ethnomed. 2022 Dec 5;18:70. doi: 10.1186/s13002-022-00566-0 (PMC9720931; doi:10.1186/s13002-022-00566-0)
Supplement: Supplementary file 1 — Additional fil 1. Supplementary material. [file 13002_2022_566_MOESM1_ESM.docx]

Supplementary material

Table 1. Mode of preparation of different ethnobotanical products in the studied area

| **Mode of preparation** | **No. of statements** | **% of formulation** |
| --- | --- | --- |
| Balm | 102 | 4.372 |
| Bath | 7 | 0.300 |
| Cold maceration | 2 | 0.086 |
| Decoction | 60 | 2.572 |
| Dry | 9 | 0.386 |
| Fresh (direct utilization) | 236 | 10.116 |
| Fresh, mixed with honey | 23 | 0.986 |
| Fresh, revetment | 108 | 4.629 |
| Infusion | 1167 | 50.021 |
| Maceration | 13 | 0.557 |
| Micellar water | 1 | 0.043 |
| Mixed with honey | 4 | 0.171 |
| Oil extract | 37 | 1.586 |
| Ointment | 10 | 0.429 |
| Powder | 7 | 0.300 |
| Processed (heat treated e.g. cooked) | 303 | 12.988 |
| Syrup | 7 | 0.300 |
| Tincture | 109 | 4.672 |
| Vinegar | 42 | 1.800 |
| Wine | 7 | 0.300 |

| Table2. The percentage of used plant parts in the studied area  (WP = Whole plant; Sht = Shoots; AP = Aerial parts; APF = Aerial parts and flowers; ApP = Apical parts; ApPL = Apical parts and leaves; FAP = Flowering apical parts; Rt = Roots; Rh = Rhizomes; Ts = Tubers; St = Stem; Tr = Trunk; PdL = Penducle and leaves; Pd = Peduncle; Lt = Latex; Bk = Bark; Br = Branches; Tg = Twigs; FR = Flowers and roots; FL = Flowers and leaves; L = Leaves; F = Flowers; Pl = Petals; PO = Pollen; Bd = Buds; Fr = Fruit; Sd = Seed; FC = Fruit with capsule) | | | | | | | | | | | | | | | | | | | | | | | | | | | | |
| --- | --- | --- | --- | --- | --- | --- | --- | --- | --- | --- | --- | --- | --- | --- | --- | --- | --- | --- | --- | --- | --- | --- | --- | --- | --- | --- | --- | --- |
| **Species** | **WP** | **Sht** | **AP** | **APF** | **ApP** | **ApPL** | **FAP** | **Rt** | **Rh** | **Ts** | **St** | **Tr** | **PdL** | **Pd** | **Lt** | **Bk** | **Br** | **Tg** | **FR** | **FL** | **L** | **F** | **Pl** | **P0** | **Bd** | **Fr** | **Sd** | **FC** |
| *Achillea clypeolata* Sm. |  |  |  |  |  |  |  |  |  |  |  |  |  |  |  |  |  |  |  |  |  | + |  |  |  |  |  |  |
| *Achillea millefolium* L. | + |  | + |  |  |  | + |  |  |  | + |  |  |  |  |  |  |  |  | + | + | + |  |  |  |  |  |  |
| *Aesculum hippocastanum* L. |  |  |  |  |  |  |  |  |  |  |  |  |  |  |  |  |  |  |  |  |  | + |  |  |  | + |  | + |
| *Agrimonia eupatoria* L. | + |  | + |  |  |  |  |  |  |  |  |  |  |  |  |  |  |  |  | + | + |  |  |  |  | + |  |  |
| *Alcea biennis* Winterl |  |  |  |  |  |  |  |  |  |  |  |  |  |  |  |  |  |  |  |  | + |  |  |  |  |  |  |  |
| *Alchemilla vulgaris* L. |  |  |  |  |  |  |  |  |  |  |  |  |  |  |  |  |  |  |  | + | + |  |  |  |  |  |  |  |
| *Allium ursinum* L. |  |  |  |  |  |  |  |  |  |  |  |  |  |  |  |  |  |  |  |  | + |  |  |  |  |  |  |  |
| *Althaea officinalis* L. |  |  | + |  |  |  |  | + |  |  |  |  |  |  |  |  |  |  |  |  |  |  |  |  |  |  |  |  |
| *Anthyllis vulneraria* L. |  |  | + |  |  |  |  |  |  |  |  |  |  |  |  |  |  |  |  |  | + |  |  |  |  |  |  |  |
| *Arctium lappa* L. |  |  |  |  |  |  |  | + |  |  |  |  |  |  |  |  |  |  |  |  | + |  |  |  |  |  |  |  |
| *Arctium minus* (Hill) Bernh. |  |  |  |  |  |  |  |  |  |  |  |  |  |  |  |  |  |  |  |  | + |  |  |  |  |  |  |  |
| *Arum maculatum* L. |  |  |  |  |  |  |  |  | + |  |  |  |  |  |  |  |  |  |  |  |  |  |  |  |  |  |  |  |
| *Asarum europaeum* L. |  |  |  |  |  |  |  | + |  |  |  |  |  |  |  |  |  |  |  |  | + |  |  |  |  |  |  |  |
| *Asparagus officinalis* L. |  |  |  |  |  |  |  | + |  |  |  |  |  |  |  |  |  |  |  |  |  |  |  |  |  |  |  |  |
| *Asplenium viride* Huds. |  |  |  |  |  |  |  |  |  |  |  |  |  |  |  |  |  |  |  |  | + |  |  |  |  |  |  |  |
| *Betula pendula* Roth |  |  |  |  |  |  |  |  |  |  |  |  |  |  |  |  |  |  |  |  | + | + |  |  | + |  |  |  |
| *Calendula officinalis* L. |  |  | + |  |  |  |  |  |  |  |  |  |  |  |  |  |  |  |  |  | + | + |  |  |  |  |  |  |
| *Centaurium erythraea* Roth. |  |  | + |  |  |  |  |  |  |  |  |  |  |  |  |  |  |  |  | + |  | + |  |  |  |  |  |  |
| *Chelidonium majus* L. | + |  | + |  |  |  |  |  |  |  |  |  |  |  | + |  |  |  |  | + | + |  |  |  |  |  |  |  |
| *Cichorium intybus* L. | + |  | + |  |  |  |  | + |  |  |  |  |  | + |  |  |  |  | + |  |  | + |  |  |  |  |  |  |
| *Clematis vitalba* L. |  |  |  |  |  |  |  |  |  |  |  |  |  |  |  |  | + |  |  |  |  |  |  |  |  |  |  |  |
| *Cornus mas* L. |  |  |  |  |  |  |  |  |  |  |  |  |  |  |  |  |  | + |  |  |  | + |  |  |  | + |  |  |
| *Corylus colurna* L. |  |  |  |  |  |  |  |  |  |  |  |  |  |  |  |  | + |  |  |  | + |  |  |  |  | + |  |  |
| *Cotinus coggygria* Scop. |  |  |  |  |  |  |  |  |  |  |  |  |  |  |  | + |  | + |  |  | + |  |  |  |  |  |  |  |
| *Crataegus monogyna* Jacq. |  |  |  |  |  |  |  |  |  |  |  |  |  |  |  | + |  |  |  |  | + | + |  |  |  | + |  |  |
| *Cydonia oblonga* Mill. |  |  |  |  |  |  |  |  |  |  |  |  |  |  |  |  |  |  |  |  | + |  |  |  |  | + |  |  |
| *Cynodon dactylon* (L.) Pers. | + |  | + |  |  |  |  | + |  |  |  |  |  |  |  |  |  |  |  |  |  |  |  |  |  |  |  |  |
| *Datura stramonium* L. |  |  |  |  |  |  |  |  |  |  |  |  |  |  |  |  |  |  |  |  | + |  |  |  |  |  |  |  |
| *Dipsacus laciniatus* L. |  |  |  |  |  |  |  |  |  |  |  |  |  |  |  |  |  |  |  |  |  |  |  |  |  |  |  |  |
| *Epilobium parviflorum* Schreb. |  |  | + |  |  |  |  |  |  |  |  |  |  |  |  |  |  |  |  |  |  | + |  |  |  |  |  |  |
| *Equisetum arvense* L. |  | + | + |  |  |  |  |  |  |  |  |  |  |  |  |  |  |  |  |  |  |  |  |  |  |  |  |  |
| *Equisetum telmateia* Ehrh. |  | + |  |  | + |  |  |  |  |  |  |  |  |  |  |  |  |  |  |  |  |  |  |  |  |  |  |  |
| *Eupatorium cannabinum* L. | + |  | + |  |  |  |  |  |  |  |  |  |  |  |  |  |  |  |  |  |  | + |  |  |  |  |  |  |
| *Euphrasia officinalis* L. | + |  | + |  |  |  |  |  |  |  |  |  |  |  |  |  |  |  |  |  |  |  |  |  |  |  |  |  |
| *Filipendula hexapetala* Gilib. |  |  |  |  |  |  |  | + |  |  |  |  |  |  |  |  |  |  |  |  | + | + |  |  |  |  |  |  |
| *Fragaria vesca* L. |  |  |  |  |  |  |  |  |  |  |  |  |  |  |  |  |  |  |  | + | + |  |  |  |  | + |  |  |
| *Galium aparine* L. |  |  | + |  |  |  |  |  |  |  |  |  |  |  |  |  |  |  |  |  |  |  |  |  |  |  |  |  |
| *Galium odoratum* (L.) Scop. |  |  |  |  |  |  |  |  |  |  |  |  |  |  |  |  |  |  |  |  |  | + |  |  |  |  |  |  |
| *Galium verum* L. |  |  | + |  |  |  |  |  |  |  |  |  |  |  |  |  |  |  |  |  |  | + |  |  |  |  |  |  |
| *Geranium macrorrhizum* L. | + |  |  |  |  |  |  |  |  |  |  |  |  |  |  |  |  |  |  | + |  | + |  |  |  |  |  |  |
| *Geranium robertianum* L. |  |  | + |  |  |  |  |  |  |  |  |  |  |  |  |  |  |  |  |  | + |  |  |  |  |  |  |  |
| *Hedera helix* L. |  |  |  |  |  |  |  |  |  | + |  |  |  |  |  |  |  | + |  |  | + |  |  |  |  | + |  |  |
| *Helianthus tuberosus* L. |  |  |  |  |  |  |  |  |  |  |  |  |  |  |  |  |  |  |  |  |  |  |  |  |  |  |  |  |
| *Helleborus odorus* WK | + |  | + |  |  |  |  | + |  |  |  |  |  |  |  |  |  |  |  |  | + | + |  |  |  |  |  |  |
| *Hieracium pilosella* L. | + |  |  |  |  |  |  |  |  |  |  |  |  |  |  |  |  |  |  |  |  |  |  |  |  |  |  |  |
| *Humulus lupulus* L. |  |  |  |  |  |  |  |  |  |  |  |  |  |  |  |  |  |  |  |  |  |  |  |  |  | + |  |  |
| *Hypericum perforatum* L. |  |  | + |  |  |  | + |  |  |  |  |  |  |  |  |  |  |  |  | + |  | + |  |  |  |  |  |  |
| *Juglans regia* L. |  |  |  |  |  |  |  |  |  |  |  |  |  |  |  |  |  |  |  |  | + |  |  |  |  | + |  |  |
| *Kickxia elatine* (L.) Dumort. | + |  | + |  |  |  |  |  |  |  |  |  |  |  |  |  |  |  |  |  |  |  |  |  |  |  |  |  |
| *Laserpitium latifolium* L. |  |  |  |  |  |  |  | + |  |  |  |  |  |  |  |  |  |  |  |  |  |  |  |  |  |  |  |  |
| *Linaria vulgaris* Mill. |  |  | + |  |  |  |  |  |  |  |  |  |  |  |  |  |  |  |  |  |  | + |  |  |  |  |  |  |
| *Loranthus europaeus* Jacq. |  |  |  |  |  |  |  |  |  |  |  |  |  |  |  |  |  |  |  |  | + |  |  |  |  |  |  |  |
| *Lotus corniculatus* L. | + |  | + |  |  |  |  |  |  |  |  |  |  |  |  |  |  |  |  |  |  |  |  |  |  |  |  |  |
| *Lysimachia nummularia* L. |  |  |  |  |  |  |  |  |  |  |  |  |  |  |  |  |  |  |  |  | + |  |  |  |  |  |  |  |
| *Lythrum salicaria* L. |  |  | + |  | + |  |  |  |  |  |  |  |  |  |  |  |  |  |  |  |  |  |  |  |  |  |  |  |
| *Malus sylvestris* (L.) Mill. |  |  |  |  |  |  |  |  |  |  |  |  |  |  |  |  |  |  |  |  |  |  |  |  |  | + |  |  |
| *Malva sylvestris* L. |  |  |  |  |  |  |  |  |  |  |  |  |  |  |  |  |  |  |  | + |  |  |  |  |  |  |  |  |
| *Melilotus albus* Medik. |  |  | + |  |  |  |  |  |  |  |  |  |  |  |  |  |  |  |  |  |  |  |  |  |  |  |  |  |
| *Melilotus officinalis* (L.) Pall. |  |  | + |  |  |  |  |  |  |  |  |  |  |  |  |  |  |  |  |  |  |  |  |  |  |  |  |  |
| *Melissa officinalis* L. |  |  | + |  | + |  |  |  |  |  |  |  |  |  |  |  |  |  |  | + | + | + |  |  |  |  |  |  |
| *Mentha longifolia* (L.) L. |  |  | + |  |  |  | + |  |  |  |  |  |  |  |  |  |  |  |  |  | + |  |  |  |  |  |  |  |
| *Ononis spinosa* L. |  |  |  |  |  |  |  | + |  |  |  |  |  |  |  |  |  |  |  |  |  |  |  |  |  |  |  |  |
| *Origanum vulgare* L. |  |  | + |  |  |  | + |  |  |  |  |  |  |  |  |  |  |  |  | + | + | + |  |  |  |  |  |  |
| *Paliurus spina-christi* Mill. |  |  |  |  |  |  |  |  |  |  |  |  |  |  |  |  |  |  |  |  |  |  |  |  |  | + |  |  |
| *Petasites albus* (L.) Gaertn. | + |  |  |  |  |  |  |  |  |  |  |  |  |  |  |  |  |  |  |  |  |  |  |  |  |  |  |  |
| *Petasites hybridus* (L.) "G.Gaertn., B.Mey. & Scherb." |  |  |  |  |  |  |  | + |  |  |  |  |  |  |  |  |  |  |  |  | + |  |  |  |  |  |  |  |
| *Peucedanum longifolium* WK | + |  |  |  |  |  |  |  |  |  |  |  |  |  |  |  |  |  |  |  |  |  |  |  |  |  |  |  |
| *Physalis alkekengi* L. |  |  |  |  |  |  |  |  |  |  |  |  |  |  |  |  |  |  |  |  |  |  |  |  |  | + |  |  |
| *Pinus nigra* J. F. Arnold. |  | + |  |  |  |  |  |  |  |  |  |  |  |  |  |  |  |  |  |  | + |  |  | + |  |  |  |  |
| *Plantago lanceolata* L. | + |  |  |  |  |  |  |  |  |  |  |  |  |  |  |  |  |  |  |  | + |  |  |  |  |  |  |  |
| *Plantago major* L. |  |  |  |  |  |  |  |  |  |  |  |  |  |  |  |  |  |  |  |  | + |  |  |  |  |  |  |  |
| *Polygonum aviculare* L. | + |  | + |  |  |  |  |  |  |  |  |  |  |  |  |  |  |  |  |  | + |  |  |  |  |  |  |  |
| *Potentilla reptans* L. |  |  |  |  |  |  |  |  |  |  |  |  |  |  |  |  |  |  |  |  | + |  |  |  |  |  |  |  |
| *Prunulla vulgaris* L. |  |  | + |  |  |  | + |  |  |  |  |  |  |  |  |  |  |  |  |  |  |  |  |  |  |  |  |  |
| *Prunus spinosa* L. |  |  |  |  |  |  |  |  |  |  |  |  |  |  |  |  |  |  |  |  |  |  |  |  |  | + |  |  |
| *Pulmonaria officinalis* L. |  |  | + |  |  |  |  |  |  |  |  |  |  |  |  |  |  |  |  | + | + | + |  |  |  |  |  |  |
| *Pyrus pyraster* (L.) Burgst. |  |  |  |  |  |  |  |  |  |  |  |  |  |  |  |  |  |  |  |  | + |  |  |  |  | + |  |  |
| *Quercus cerris* L. |  |  |  |  |  |  |  |  |  |  |  | + |  |  |  | + |  |  |  |  |  |  |  |  |  |  |  |  |
| *Robinia pseudoacacia* L. |  |  |  |  |  |  |  |  |  |  |  |  |  |  |  |  |  |  |  |  |  | + |  |  |  |  |  |  |
| *Rosa canina* L. |  |  |  |  |  |  |  |  |  |  |  |  |  |  |  |  |  |  |  |  |  |  | + |  |  | + |  |  |
| *Rubus caesius* L. |  |  |  |  |  |  |  |  |  |  |  |  |  |  |  |  |  |  |  |  | + |  |  |  |  |  |  |  |
| *Rubus plicatus* Weihe & Nees |  |  |  |  |  |  |  |  |  |  |  |  |  |  |  |  |  |  |  |  | + |  |  |  |  | + |  |  |
| *Rubus ulmifolius* Schott |  |  |  |  |  |  |  |  |  |  |  |  |  |  |  |  |  |  |  | + | + |  |  |  |  | + |  |  |
| *Rumex acetosa* L. |  |  |  |  |  |  |  |  |  |  |  |  |  |  |  |  |  |  |  |  | + |  |  |  |  |  |  |  |
| *Rumex crispus* L. | + |  |  |  |  |  |  |  |  |  |  |  |  |  |  |  |  |  |  |  |  |  |  |  |  |  | + |  |
| *Rumex patientia* L. |  |  |  |  |  |  |  |  |  |  |  |  |  |  |  |  |  |  |  |  | + |  |  |  |  |  | + |  |
| *Salix alba* L. |  |  |  |  |  |  |  |  |  |  |  |  |  |  |  | + |  | + |  |  |  |  |  |  |  |  |  |  |
| *Salix purpurea* L. |  |  |  |  |  |  |  |  |  |  |  |  |  |  |  | + |  | + |  |  |  |  |  |  |  |  |  |  |
| *Sambucus ebulus* L. |  |  |  |  |  |  |  | + |  |  |  |  |  |  |  |  |  |  |  |  |  |  |  |  |  |  |  |  |
| *Sambucus nigra* L. |  |  |  |  |  |  |  |  |  |  |  |  |  |  |  |  |  | + |  |  |  | + |  |  |  | + |  |  |
| *Satureja subspicata* Bartl. ex Vis. |  |  | + |  |  |  |  |  |  |  |  |  |  |  |  |  |  |  |  | + |  |  |  |  |  |  |  |  |
| *Sempervivum tectorum* L. |  |  |  |  |  |  |  |  |  |  |  |  |  |  |  |  |  |  |  |  | + |  |  |  |  |  |  |  |
| *Sorbus aucuparia* L. |  |  |  |  |  |  |  |  |  |  |  |  |  |  |  |  |  |  |  |  |  |  |  |  |  | + |  |  |
| *Stachys officinalis* (L.) Trevis. |  |  | + | + |  |  |  |  |  |  |  |  |  |  |  |  |  |  |  | + | + | + |  |  |  |  |  |  |
| *Symphytum officinale* L. |  |  |  |  |  |  |  | + |  |  |  |  |  |  |  |  |  |  |  |  | + |  |  |  |  |  |  |  |
| *Tanacetum vulgare* L. |  |  | + |  |  |  | + |  |  |  |  |  |  |  |  |  |  |  |  |  | + | + |  |  |  |  |  |  |
| Taraxacum *sect.* Ruderalia |  |  |  |  |  |  |  | + |  |  | + |  | + |  |  |  |  |  |  |  | + | + |  |  |  |  |  |  |
| *Teucrium chamaedrys* L. |  |  | + |  |  |  |  |  |  |  |  |  |  |  |  |  |  |  |  | + |  | + |  |  |  |  |  |  |
| *Teucrium montanum* L. | + |  | + |  |  |  |  |  |  |  |  |  |  |  |  |  |  |  |  | + |  |  |  |  |  |  |  |  |
| *Thymus serphyllum* L. | + |  | + |  |  |  |  |  |  |  |  |  |  |  |  |  |  |  |  | + |  | + |  |  |  |  |  |  |
| *Tilia platyphyllos* Scop. |  |  |  |  |  |  |  |  |  |  |  | + |  |  |  |  |  | + |  |  | + | + |  |  |  |  |  |  |
| *Tussilago farfara* L. |  |  |  |  |  |  |  |  |  |  |  |  |  |  |  |  |  |  |  |  | + |  |  |  |  |  |  |  |
| *Trifolium pratense* L. |  |  |  |  |  |  | + |  |  |  |  |  |  |  |  |  |  |  |  |  |  | + |  |  |  |  |  |  |
| *Trifolium repens* L. | + |  | + |  |  |  |  |  |  |  |  |  |  |  |  |  |  |  |  |  |  | + |  |  |  |  |  |  |
| *Urtica dioica* L. | + |  | + |  | + | + |  | + |  |  |  |  |  |  |  |  |  |  |  |  | + |  |  |  |  | + | + |  |
| *Vaccinium vitis-idaea* L. |  |  |  |  |  |  |  |  |  |  |  |  |  |  |  |  |  |  |  |  | + |  |  |  |  | + |  |  |
| *Valeriana officinalis* L. |  |  |  |  |  |  |  | + |  |  |  |  |  |  |  |  |  |  |  |  |  |  |  |  |  |  |  |  |
| *Verbascum thapsus* L. | + |  |  |  |  |  |  |  |  |  |  |  |  |  |  |  |  |  |  |  |  |  |  |  |  |  |  |  |
| *Verbena officinalis* L. |  |  | + |  |  |  |  |  |  |  |  |  |  |  |  |  |  |  |  |  |  |  |  |  |  |  |  |  |
| *Veronica officinalis* L. |  |  | + |  |  |  |  |  |  |  |  |  |  |  |  |  |  |  |  |  |  |  |  |  |  |  |  |  |
| *Vicia cracca* L. | + |  | + |  |  |  |  |  |  |  |  |  |  |  |  |  |  |  |  |  |  |  |  |  |  |  |  |  |
| *Viola odorata* L. |  |  |  |  |  |  |  |  |  |  |  |  |  |  |  |  |  |  |  | + |  |  |  |  |  |  |  |  |
| *Xantium spinosum* L. |  |  | + |  |  |  |  |  |  |  |  |  |  |  |  |  |  |  |  |  |  |  |  |  |  |  |  |  |
| *Xeranthemum cylindraceum* Sm. |  |  | + |  |  |  |  |  |  |  |  |  |  |  |  |  |  |  |  |  |  |  |  |  |  |  |  |  |
| Σ114/No. | 23 | 3 | 43 | 1 | 4 | 1 | 7 | 16 | 1 | 1 | 2 | 2 | 1 | 1 | 1 | 5 | 2 | 7 | 1 | 19 | 51 | 31 | 1 | 1 | 1 | 22 | 3 | 1 |
| % | 20.1 | 2.6 | 37.7 | 0.8 | 3.5 | 0.8 | 6.1 | 14.0 | 0.8 | 0.8 | 1.7 | 1.7 | 0.8 | 0.8 | 0.8 | 4.3 | 1.7 | 6.1 | 0.8 | 16.6 | 44.7 | 27.1 | 0.8 | 0.8 | 0.8 | 19.3 | 2.6 | 0.8 |

Table 3. Frequency of citation (FC), relative frequency of citation (RFC) and relative importance index (RI) of plant species in the Aleksinac district

| **Botanical name** | **FC** | **RFC** | **RI** |
| --- | --- | --- | --- |
| *Achillea millefolium* L. | 4.90 | 0.089 | 0.311 |
| *Agrimonia eupatoria* L. | 1.55 | 0.028 | 0.309 |
| *Alcea biennis* Winterl | 0.52 | 0.009 | 0.145 |
| *Allium ursinum L.* | 1.80 | 0.033 | 0.319 |
| *Althaea officinalis* L. | 0.77 | 0.014 | 0.154 |
| *Arctium minus* (Hill) Bernh. | 0.26 | 0.005 | 0.135 |
| *Asarum europaeum* L. | 0.26 | 0.005 | 0.135 |
| *Asplenium viride* Huds. | 0.26 | 0.005 | 0.135 |
| *Betula pendula* Roth | 1.03 | 0.019 | 0.164 |
| *Calendula officinalis* L. | 2.84 | 0.052 | 0.358 |
| *Centaurium erythraea* Roth. | 0.52 | 0.009 | 0.145 |
| *Chelidonium majus* L. | 1.55 | 0.028 | 0.184 |
| *Cichorium intybus* L. | 2.58 | 0.047 | 0.473 |
| *Clematis vitalba* L. | 1.03 | 0.019 | 0.164 |
| *Cornus mas* L. | 1.03 | 0.019 | 0.414 |
| *Crataegus monogyna* Jacq. | 1.80 | 0.033 | 0.319 |
| *Cydonia oblonga* Mill. | 2.58 | 0.047 | 0.473 |
| *Cynodon dactylon* (L.) Pers. | 1.55 | 0.028 | 0.309 |
| *Datura stramonium* L. | 0.26 | 0.005 | 0.135 |
| *Equisetum arvense* L. | 1.80 | 0.033 | 0.194 |
| *Equisetum telmateia* Ehrh. | 0.52 | 0.009 | 0.145 |
| *Fragaria vesca* L. | 0.77 | 0.014 | 0.154 |
| *Galium aparine* L. | 0.52 | 0.009 | 0.145 |
| *Hedera helix* L. | 0.77 | 0.014 | 0.154 |
| *Helianthus tuberosus* L. | 0.26 | 0.005 | 0.135 |
| *Helleborus odorus* WK | 1.29 | 0.023 | 0.299 |
| *Hypericum perforatum* L. | 13.14 | 0.239 | 0.750 |
| *Juglans regia* L. | 2.32 | 0.042 | 0.213 |
| *Kickxia elatine* (L.) Dumort. | 0.52 | 0.009 | 0.145 |
| *Loranthus europaeus* Jacq. | 0.52 | 0.009 | 0.145 |
| *Lythrum salicaria* L. | 0.52 | 0.009 | 0.270 |
| *Malus sylvestris* (L.) Mill. | 0.26 | 0.005 | 0.135 |
| *Malva sylvestris* L. | 0.26 | 0.005 | 0.135 |
| *Melissa officinalis* L. | 3.09 | 0.056 | 0.493 |
| *Mentha longifolia* (L.) L. | 0.26 | 0.005 | 0.135 |
| *Ononis spinosa* L. | 0.77 | 0.014 | 0.154 |
| *Origanum vulgare* L. | 1.03 | 0.019 | 0.164 |
| *Paliurus spina-christi* Mill. | 0.77 | 0.014 | 0.154 |
| *Petasites albus* (L.) Gaertn. | 0.26 | 0.005 | 0.135 |
| *Physalis alkekengi* L. | 1.03 | 0.019 | 0.289 |
| *Pinus nigra* J.F.Arnold. | 0.77 | 0.014 | 0.154 |
| *Plantago lanceolata* L. | 1.80 | 0.033 | 0.319 |
| *Plantago major* L. | 5.15 | 0.094 | 0.446 |
| *Polygonum aviculare* L. | 1.03 | 0.019 | 0.289 |
| *Potentilla reptans* L. | 0.26 | 0.005 | 0.135 |
| *Prunella vulgaris* L. | 0.77 | 0.014 | 0.279 |
| *Prunus spinosa* L. | 0.77 | 0.014 | 0.279 |
| *Pulmonaria officinalis* L. | 0.26 | 0.005 | 0.135 |
| *Rosa canina* L. | 4.38 | 0.080 | 0.417 |
| *Rubus caesius* L. | 0.26 | 0.005 | 0.135 |
| *Rubus ulmifolius* Schott | 2.32 | 0.042 | 0.338 |
| *Rumex crispus* L. | 0.26 | 0.005 | 0.135 |
| *Salix purpurea* L. | 0.77 | 0.014 | 0.279 |
| *Sambucus ebulus* L. | 0.52 | 0.009 | 0.270 |
| *Sambucus nigra* L. | 4.64 | 0.084 | 0.427 |
| *Symphytum officinale* L. | 1.55 | 0.028 | 0.184 |
| *Taraxacum* sect. *Ruderalia* | 1.29 | 0.023 | 0.174 |
| *Teucrium chamaedrys* L. | 0.26 | 0.005 | 0.135 |
| *Tilia platyphyllos* Scop. | 2.84 | 0.052 | 0.358 |
| *Tussilago farfara* L. | 0.26 | 0.005 | 0.135 |
| *Urtica dioica* L. | 9.02 | 0.164 | 0.843 |
| *Verbascum thapsus* L. | 0.52 | 0.009 | 0.270 |
| *Verbena officinalis* L. | 0.77 | 0.014 | 0.154 |
| *Viola odorata* L. | 0.26 | 0.005 | 0.135 |
| *Xantium spinosum* L. | 0.52 | 0.009 | 0.270 |
| *Xeranthemum cylindraceum* Sm. | 1.03 | 0.019 | 0.289 |

Table 4. Frequency of citation (FC), relative frequency of citation (RFC) and relative importance index (RI) of plant species in the Bor district

| **Botanical name** | **FC** | **RFC** | **RI** |
| --- | --- | --- | --- |
| *Achillea clypeolata* Sm. | 0.57 | 0.006 | 0.160 |
| *Achillea millefolium* L. | 4.42 | 0.044 | 0.651 |
| *Aesculum hippocastanum* L. | 0.72 | 0.007 | 0.170 |
| *Agrimonia eupatoria* L. | 0.82 | 0.008 | 0.176 |
| *Alcea biennis* Winterl | 0.05 | 0.001 | 0.128 |
| *Alchemilla vulgaris* L. | 0.26 | 0.003 | 0.141 |
| *Anthyllis vulneraria* L. | 0.10 | 0.001 | 0.131 |
| *Arctium lappa* L. | 0.72 | 0.007 | 0.170 |
| *Arum maculatum* L. | 0.10 | 0.001 | 0.131 |
| *Asarum europaeum* L. | 0.31 | 0.003 | 0.394 |
| *Asparagus officinalis* L. | 0.10 | 0.001 | 0.256 |
| *Betula pendula* Roth | 0.67 | 0.007 | 0.167 |
| *Calendula officinalis* L. | 1.44 | 0.014 | 0.465 |
| *Centaurium erythraea* Roth. | 1.03 | 0.010 | 0.189 |
| *Chelidonium majus* L. | 1.65 | 0.016 | 0.353 |
| *Cichorium intybus* L. | 1.23 | 0.012 | 0.327 |
| *Cornus mas* L. | 2.37 | 0.024 | 0.522 |
| *Corylus colurna* L. | 0.36 | 0.004 | 0.272 |
| *Cotinus coggygria* Scop. | 1.13 | 0.011 | 0.196 |
| *Dipsacus laciniatus* L. | 0.05 | 0.001 | 0.128 |
| *Epilobium parviflorum* Schreb. | 0.36 | 0.004 | 0.147 |
| *Equisetum arvense* L. | 1.18 | 0.012 | 0.324 |
| *Eupatorium cannabium* L. | 0.36 | 0.004 | 0.147 |
| *Euphrasia officinalis* L. | 0.15 | 0.002 | 0.135 |
| *Filipendula hexapetala* Gilib. | 0.41 | 0.004 | 0.151 |
| *Fragaria vesca* L. | 2.78 | 0.028 | 0.423 |
| *Galium odoratum* (L.) Scop. | 0.31 | 0.003 | 0.144 |
| *Galium verum* L. | 1.59 | 0.016 | 0.349 |
| *Geranium macrorrhizum* L. | 0.31 | 0.003 | 0.269 |
| *Geranium robertianum* L. | 0.41 | 0.004 | 0.151 |
| *Hedera helix* L. | 0.46 | 0.005 | 0.154 |
| *Helleborus odorus* WK | 0.15 | 0.002 | 0.260 |
| *Hieracium pilosella* L. | 0.05 | 0.001 | 0.128 |
| *Humulus lupulus* L. | 0.21 | 0.002 | 0.263 |
| *Hypericum perforatum* L. | 6.89 | 0.069 | 0.680 |
| *Lasterpitium latifolium* L. | 0.21 | 0.002 | 0.138 |
| *Linaria vulgaris* Mill. | 0.36 | 0.004 | 0.147 |
| *Lotus corniculatus* L. | 0.15 | 0.002 | 0.135 |
| *Lysimachia nummularia* L. | 0.05 | 0.001 | 0.128 |
| *Lythrum salicaria* L. | 0.05 | 0.001 | 0.128 |
| *Malus sylvestris* (L.) Mill. | 0.98 | 0.010 | 0.311 |
| *Melilotus albus* Medik. | 0.26 | 0.003 | 0.141 |
| *Melilotus officinalis* (L.) Pall. | 0.46 | 0.005 | 0.279 |
| *Melissa officinalis* L. | 2.98 | 0.030 | 0.561 |
| *Mentha longifolia* (L.) L. | 0.72 | 0.007 | 0.170 |
| *Origanum vulgare* L. | 1.39 | 0.014 | 0.337 |
| *Petasites hybridus* (L.) "G.Gaertn., B.Mey. & Scherb." | 0.57 | 0.006 | 0.160 |
| *Peucedanum longifolium* WK | 0.21 | 0.002 | 0.138 |
| *Pinus nigra* J.F.Arnold. | 0.26 | 0.003 | 0.266 |
| *Plantago lanceolata* L. | 0.36 | 0.004 | 0.147 |
| *Plantago major* L. | 3.34 | 0.033 | 0.583 |
| *Polygonum aviculare* L. | 0.51 | 0.005 | 0.282 |
| *Prunus spinosa* L. | 1.29 | 0.013 | 0.330 |
| *Pulmonaria officinalis* L. | 0.62 | 0.006 | 0.163 |
| *Pyrus pyraster* (L.) Burgst. | 0.36 | 0.004 | 0.272 |
| *Quercus cerris* L. | 0.36 | 0.004 | 0.522 |
| *Robinia pseudoacacia* L. | 0.57 | 0.006 | 0.285 |
| *Rosa canina* L. | 4.99 | 0.050 | 0.686 |
| *Rubus plicatus* Weihe & Nees | 3.91 | 0.039 | 0.494 |
| *Rumex acetosa* L. | 0.26 | 0.003 | 0.266 |
| *Rumex patientia* L. | 2.37 | 0.024 | 0.647 |
| *Salix alba* L. | 0.46 | 0.005 | 0.404 |
| *Sambucus nigra* L. | 6.07 | 0.061 | 0.628 |
| *Satureja subspicata* Bartl. ex Vis. | 0.87 | 0.009 | 0.304 |
| *Sempervivum tectorum* L. | 2.31 | 0.023 | 0.519 |
| *Sorbus aucuparia* L. | 0.57 | 0.006 | 0.160 |
| *Stachys officinalis* (L.) Trevis. | 1.08 | 0.011 | 0.192 |
| *Symphytum officinale* L. | 1.80 | 0.018 | 0.237 |
| *Tanacetum vulgare* L. | 0.31 | 0.003 | 0.394 |
| *Taraxacum* sect. *Ruderalia* | 4.16 | 0.042 | 0.635 |
| *Teucrium chamaedrys* L. | 1.23 | 0.012 | 0.327 |
| *Teucrium montanum* L. | 0.77 | 0.008 | 0.173 |
| *Thymus serphyllum* L. | 3.55 | 0.035 | 0.471 |
| *Tilia platyphyllos* Scop. | 4.01 | 0.040 | 0.625 |
| *Tussilago farfara* L. | 0.77 | 0.008 | 0.173 |
| *Trifolium pratense* L. | 0.36 | 0.004 | 0.272 |
| *Trifolium repens* L. | 0.41 | 0.004 | 0.276 |
| *Urtica dioica* L. | 8.02 | 0.080 | 1.000 |
| *Vaccinium vitis-idaea* L. | 0.82 | 0.008 | 0.301 |
| *Valeriana officinalis* L. | 0.36 | 0.004 | 0.147 |
| *Verbena officinalis* L. | 0.46 | 0.005 | 0.154 |
| *Veronica officinalis* L. | 0.21 | 0.002 | 0.138 |
| *Vicia cracca* L. | 0.10 | 0.001 | 0.131 |

Table 5. Informant consensus factor by categories of health related disorders recorded in Aleksinac and Bor districts (n=55 and n=100)

| Aleksinac (n=55) | | | | Bor  (n=100) | | | |
| --- | --- | --- | --- | --- | --- | --- | --- |
| Health and Organs Systems | Nur | Nt | ICF (%) | Health and Organs Systems | Nur | Nt | ICF (%) |
| Endocrine system disorders – ESD | 5 | 1 | 100 | Skin related disorders – SA | 166 | 20 | 88.48 |
| Skin ailments – SA | 60 | 13 | 79.66 | Digestive system disorders – DSD | 268 | 45 | 83.52 |
| Circulatory system disorders – CSD | 37 | 12 | 69.44 | Respiratory system disorders – ResSD | 189 | 36 | 81.38 |
| Immune system disorders – ISD | 30 | 11 | 65.52 | Nervous system disorders – NSD | 127 | 25 | 80.95 |
| Nervous system disorders – NSD | 18 | 9 | 52.94 | Immune system disorders – ISD | 132 | 26 | 80.92 |
| Respiratory system disorders – ResSD | 30 | 15 | 51.72 | Tumors  – TA | 53 | 12 | 78.85 |
| Tumor ailments – TA | 3 | 2 | 50.00 | Circulatory system disorders – CSD | 150 | 38 | 75.17 |
| Urinary system disorders - USD | 27 | 15 | 46.15 | Musculoskeletal system disorders – MSSD | 53 | 15 | 73.08 |
| Digestive system disorders – DSD | 40 | 23 | 43.59 | Sensory system disorders – SS | 24 | 8 | 69.56 |
| Reproductive system disorders - RepSD | 10 | 7 | 33.33 | Metabolic disorders – MD | 33 | 12 | 65.62 |
| Sensory system disorders - SS | 5 | 4 | 25.00 | Urinary system disorders – USD | 84 | 31 | 63.85 |
| General health - GH | 11 | 9 | 20.00 | General health – GH | 44 | 21 | 53.49 |
| Musculoskeletal system disorders - MSSD | 6 | 5 | 20.00 | Reproductive system disorders – RepSD | 26 | 14 | 48.00 |
| Antimicrobial activity - AMA | 1 | 1 | 0.00 | Endocrine system disorders – ESD | 8 | 5 | 42.86 |
| Metabolic disorders - MD | 1 | 1 | 0.00 | Antimicrobial activity – AMA | 12 | 8 | 36.36 |

Table 6. Informant consensus factor by categories of health disorders for the entire studied area   (n=155)

| Organ system | Nur | Nt | ICF (%) |
| --- | --- | --- | --- |
| Skin ailments – SA | 226 | 26 | 88.89 |
| Respiratory system disorders – ResSD | 219 | 40 | 82.11 |
| Digestive system disorders – DSD | 308 | 56 | 82.08 |
| Immune system disorders – ISD | 162 | 31 | 81.37 |
| Nervous system disorders – NSD | 145 | 28 | 81.25 |
| Tumor ailments – TA | 56 | 12 | 80.00 |
| Circulatory system disorders – CSD | 187 | 47 | 75.27 |
| Musculoskeletal system disorders – MSSD | 59 | 18 | 70.69 |
| Urinary system disorders – USD | 111 | 38 | 66.36 |
| Metabolic disorders – MD | 34 | 13 | 63.64 |
| Sensory system disorders – SS | 29 | 12 | 60.71 |
| Endocrine system disorders – ESD | 13 | 6 | 58.33 |
| General health – GH | 55 | 26 | 53.70 |
| Reproductive system disorders – RepSD | 36 | 18 | 51.43 |
| Antiseptic activity - ASA | 13 | 9 | 33.33 |

Table 7. Informant consensus factor by categories of health related disorders recorded in Aleksinac district for men and women (n=55)

| Men (n=21) | | | | Women  (n=34) | | | |
| --- | --- | --- | --- | --- | --- | --- | --- |
| Health and Organs Systems | Nur | Nt | ICF (%) | Health and Organs Systems | Nur | Nt | ICF (%) |
| ESD | 3 | 1 | 100.00 | ESD | 2 | 1 | 100.00 |
| SA | 22 | 7 | 71.43 | TA | 2 | 1 | 100.00 |
| CSD | 9 | 5 | 50.00 | SA | 38 | 9 | 78.38 |
| ResSD | 5 | 4 | 25.00 | ISD | 20 | 8 | 63.16 |
| ISD | 10 | 8 | 22.22 | CSD | 27 | 12 | 57.69 |
| NSD | 7 | 6 | 16.67 | NSD | 11 | 6 | 50.00 |
| DSD | 11 | 10 | 10.00 | USD | 21 | 11 | 50.00 |
| MD | 1 | 1 | 0.00 | ResSD | 25 | 14 | 45.83 |
| MSSD | 2 | 2 | 0.00 | DSD | 29 | 17 | 42.86 |
| SS | 2 | 2 | 0.00 | RepSD | 10 | 7 | 33.33 |
| TA | 1 | 1 | 0.00 | GH | 11 | 9 | 20.00 |
| USD | 6 | 6 | 0.00 | MSSD | 4 | 4 | 0.00 |
|  |  |  |  | SS | 3 | 3 | 0.00 |
|  |  |  |  | AMA | 1 | 1 | - |

Table 8. Informant consensus factor by categories of health related disorders recorded in Bor district for men and women (n=100)

| Men (n=21) | | | | Women (n=79) | | | |
| --- | --- | --- | --- | --- | --- | --- | --- |
| Health and Organs Systems | Nur | Nt | ICF (%) | Health and Organs Systems | Nur | Nt | ICF (%) |
| SS | 8 | 2 | 85.71 | SA | 138 | 19 | 86.86 |
| SA | 28 | 7 | 77.78 | DSD | 216 | 38 | 82.79 |
| ISD | 28 | 11 | 62.96 | ISD | 104 | 20 | 81.55 |
| MSSD | 14 | 6 | 61.54 | ResSD | 162 | 33 | 80.12 |
| NSD | 28 | 13 | 55.56 | NSD | 99 | 22 | 78.57 |
| DSD | 52 | 24 | 54.90 | TA | 36 | 10 | 74.29 |
| ResSD | 27 | 14 | 50.00 | CSD | 127 | 35 | 73.02 |
| CSD | 23 | 13 | 45.45 | MSSD | 39 | 14 | 65.79 |
| TA | 12 | 8 | 36.36 | USD | 70 | 28 | 60.87 |
| RepSD | 4 | 3 | 33.33 | MD | 29 | 12 | 60.71 |
| GH | 14 | 11 | 23.08 | SS | 16 | 8 | 53.33 |
| USD | 14 | 11 | 23.08 | RepSD | 22 | 12 | 47.62 |
| MD | 4 | 4 | 0.00 | ESD | 8 | 5 | 42.86 |
| ASA | 1 | 1 | - | GH | 30 | 18 | 41.38 |
|  |  |  |  | ASA | 11 | 8 | 30.00 |

Table 9. Informant consensus factor by categories of health related disorders recorded in Aleksinac and Bor districts for men and women (n=155)

| Men (n=42) | | | | Women (n=113) | | | |
| --- | --- | --- | --- | --- | --- | --- | --- |
| Health and Organs Systems | Nur | Nt | ICF (%) | Health and Organs Systems | Nur | Nt | ICF (%) |
| ESD | 3 | 1 | 100.00 | SA | 176 | 22 | 88.00 |
| SA | 50 | 11 | 79.59 | ISD | 124 | 24 | 81.30 |
| ISD | 37 | 12 | 69.44 | ResSD | 187 | 38 | 80.11 |
| RepSD | 4 | 2 | 66.67 | NSD | 110 | 23 | 79.82 |
| SS | 10 | 4 | 66.67 | DSD | 245 | 51 | 79.51 |
| NSD | 35 | 13 | 64.71 | TA | 43 | 11 | 76.19 |
| MSSD | 16 | 7 | 60.00 | CSD | 155 | 44 | 72.08 |
| DSD | 63 | 27 | 58.06 | MSSD | 43 | 16 | 64.29 |
| CSD | 32 | 17 | 48.39 | USD | 91 | 34 | 63.33 |
| ResSD | 32 | 17 | 48.39 | MD | 29 | 13 | 57.14 |
| TA | 13 | 8 | 41.67 | RepSD | 32 | 16 | 51.61 |
| GH | 14 | 11 | 23.08 | GH | 41 | 23 | 45.00 |
| USD | 20 | 17 | 15.79 | ESD | 10 | 6 | 44.44 |
| MD | 5 | 5 | 0.00 | SS | 19 | 11 | 44.44 |
| ASA | 1 | 1 | - | ASA | 12 | 9 | 27.27 |

Table 10. Informant consensus factor by categories of health related disorders recorded in Aleksinac district for city and surrounding villages (n=55)

| City (n=10) | | | | Surrounding villages (n=45) | | | |
| --- | --- | --- | --- | --- | --- | --- | --- |
| Health and Organs Systems | Nur | Nt | ICF (%) | Health and Organs Systems | Nur | Nt | ICF (%) |
| ESD | 2 | 1 | 100.00 | ESD | 3 | 1 | 100.00 |
| SA | 13 | 5 | 66.67 | SA | 47 | 10 | 80.43 |
| DSD | 8 | 5 | 42.86 | ISD | 29 | 10 | 67.86 |
| CSD | 6 | 5 | 20.00 | CSD | 31 | 11 | 66.67 |
| MSSD | 2 | 2 | 0.00 | NSD | 17 | 8 | 56.25 |
| RepSD | 2 | 2 | 0.00 | SS | 3 | 2 | 50.00 |
| ResSD | 2 | 2 | 0.00 | TA | 3 | 2 | 50.00 |
| SS | 2 | 2 | 0.00 | USD | 23 | 12 | 50.00 |
| USD | 4 | 4 | 0.00 | ResSD | 28 | 15 | 48.15 |
| GH | 1 | 1 | - | DSD | 32 | 19 | 41.94 |
| ISD | 1 | 1 | - | RepSD | 8 | 6 | 28.57 |
| NSD | 1 | 1 | - | GH | 10 | 8 | 22.22 |
|  |  |  |  | MSSD | 4 | 4 | 0.00 |
|  |  |  |  | AMA | 1 | 1 | - |

Table 11. Informant consensus factor by categories of health related disorders recorded in Bor district for city and surrounding villages (n=100)

| City (n=50) | | | | Surrounding villages (n=50) | | | |
| --- | --- | --- | --- | --- | --- | --- | --- |
| Health and Organs Systems | Nur | Nt | ICF (%) | Health and Organs Systems | Nur | Nt | ICF (%) |
| SA | 91 | 19 | 80.00 | SS | 9 | 2 | 87.50 |
| DSD | 168 | 40 | 76.65 | SA | 75 | 14 | 82.43 |
| ISD | 83 | 22 | 74.39 | ResSD | 67 | 16 | 77.27 |
| ResSD | 122 | 32 | 74.38 | ISD | 49 | 12 | 77.08 |
| NSD | 75 | 22 | 71.62 | NSD | 51 | 13 | 76.00 |
| TA | 33 | 11 | 68.75 | DSD | 99 | 29 | 71.43 |
| CSD | 98 | 32 | 68.04 | MSSD | 28 | 9 | 70.37 |
| USD | 61 | 26 | 58.33 | TA | 20 | 7 | 68.42 |
| SS | 15 | 7 | 57.14 | CSD | 52 | 23 | 56.86 |
| MSSD | 25 | 12 | 54.17 | USD | 23 | 12 | 50.00 |
| MD | 27 | 13 | 53.85 | GH | 19 | 11 | 44.44 |
| RepSD | 13 | 7 | 50.00 | RepSD | 13 | 10 | 25.00 |
| ESD | 8 | 5 | 42.86 | MD | 6 | 6 | 0.00 |
| AMA | 11 | 7 | 40.00 | AM | 1 | 1 | - |
| GH | 25 | 18 | 29.17 |  | | | |

Table 12. Informant consensus factor by categories of health related disorders recorded in Aleksinac and Bor districts for city and surrounding villages (n=155)

| City (n=60) | | | | Surrounding villages (n=95) | | | |
| --- | --- | --- | --- | --- | --- | --- | --- |
| Health and Organs Systems | Nur | Nt | ICF (%) | Health and Organs Systems | Nur | Nt | ICF (%) |
| SA | 104 | 19 | 82.52 | ESD | 3 | 1 | 100.00 |
| DSD | 174 | 44 | 75.14 | SA | 122 | 17 | 86.78 |
| ResSD | 124 | 32 | 74.80 | ISD | 78 | 17 | 79.22 |
| ISD | 84 | 23 | 73.49 | NSD | 68 | 17 | 76.12 |
| NSD | 78 | 22 | 72.73 | ResSD | 95 | 24 | 75.53 |
| CSD | 104 | 32 | 69.90 | SS | 12 | 4 | 72.73 |
| TA | 33 | 11 | 68.75 | DSD | 131 | 37 | 72.31 |
| USD | 65 | 26 | 60.94 | TA | 23 | 8 | 68.18 |
| MD | 27 | 12 | 57.69 | MSSD | 32 | 12 | 64.52 |
| MSSD | 27 | 13 | 53.85 | CSD | 83 | 31 | 63.41 |
| SS | 17 | 9 | 50.00 | USD | 46 | 22 | 53.33 |
| ESD | 10 | 6 | 44.44 | GH | 29 | 16 | 46.43 |
| RepSD | 15 | 9 | 42.86 | RepSD | 21 | 13 | 40.00 |
| ASA | 11 | 7 | 40.00 | MD | 7 | 6 | 16.67 |
| GH | 26 | 19 | 28.00 | ASA | 2 | 2 | 0.00 |

Table 13. Use value of plant species mentioned in Aleksinac district in the present study

| **Scientific botanical name** | **UV** |
| --- | --- |
| *Achillea millefolium* L. | 0.291 |
| *Agrimonia eupatoria* L. | 0.109 |
| *Alcea biennis* Winterl | 0.036 |
| *Allium ursinum* L. | 0.127 |
| *Althaea officinalis* L. | 0.055 |
| *Arctium minus* (Hill) Bernh. | 0.018 |
| *Asarum europaeum* L. | 0.018 |
| *Asplenium viride* Huds. | 0.018 |
| *Betula pendula* Roth | 0.073 |
| *Calendula officinalis* L. | 0.182 |
| *Centaurium erythraea* Roth. | 0.036 |
| *Chelidonium majus* L. | 0.109 |
| *Cichorium intybus* L. | 0.182 |
| *Clematis vitalba* L. | 0.073 |
| *Cornus mas* L. | 0.073 |
| *Crataegus monogyna* Jacq. | 0.091 |
| *Cydonia oblonga* Mill. | 0.182 |
| *Cynodon dactylon* (L.) Pers. | 0.109 |
| *Datura stramonium* L. | 0.018 |
| *Equisetum arvense* L. | 0.109 |
| *Equisetum telmateia* Ehrh. | 0.036 |
| *Fragaria vesca* L. | 0.055 |
| *Galium aparine* L. | 0.036 |
| *Hedera helix* L. | 0.055 |
| *Helianthus tuberosus* L. | 0.018 |
| *Helleborus odorus* WK | 0.091 |
| *Hypericum perforatum* L. | 0.782 |
| *Juglans regia* L. | 0.127 |
| *Kickxia elatine* (L.) Dumort. | 0.036 |
| *Loranthus europaeus* Jacq. | 0.036 |
| *Lythrum salicaria* L. | 0.036 |
| *Malus sylvestris* (L.) Mill. | 0.018 |
| *Malva sylvestris* L. | 0.018 |
| *Melissa officinalis* L. | 0.218 |
| *Mentha longifolia* (L.) L. | 0.018 |
| *Ononis spinosa* L. | 0.055 |
| *Origanum vulgare* L. | 0.073 |
| *Paliurus spina-christi* Mill. | 0.055 |
| *Petasites albus* (L.) Gaertn. | 0.018 |
| *Physalis alkekengi* L. | 0.073 |
| *Pinus nigra* J.F.Arnold. | 0.055 |
| *Plantago lanceolata* L. | 0.109 |
| *Plantago major* L. | 0.345 |
| *Polygonum aviculare* L. | 0.073 |
| *Potentilla reptans* L. | 0.018 |
| *Prunella vulgaris* L. | 0.055 |
| *Prunus spinosa* L. | 0.055 |
| *Pulmonaria officinalis* L. | 0.018 |
| *Rosa canina* L. | 0.273 |
| *Rubus caesius* L. | 0.018 |
| *Rubus ulmifolius* Schott | 0.145 |
| *Rumex crispus* L. | 0.018 |
| *Salix purpurea* L. | 0.055 |
| *Sambucus ebulus* L. | 0.036 |
| *Sambucus nigra* L. | 0.327 |
| *Symphytum officinale* L. | 0.073 |
| *Taraxacum* sect. *Ruderalia* | 0.091 |
| *Teucrium chamaedrys* L. | 0.018 |
| *Tilia platyphyllos* Scop. | 0.200 |
| *Tussilago farfara* L. | 0.018 |
| *Urtica dioica* L. | 0.618 |
| *Verbascum thapsus* L. | 0.036 |
| *Verbena officinalis* L. | 0.055 |
| *Viola odorata* L. | 0.018 |
| *Xantium spinosum* L. | 0.036 |
| *Xeranthemum cylindraceum* Sm. | 0.073 |

Table 14. Use value of plant species mentioned in Bor district in the present study

| **Scientific botanical name** | **UV** |
| --- | --- |
| *Achillea clypeolata* Sm. | 0.090 |
| *Achillea millefolium* L. | 0.690 |
| *Aesculum hippocastanum* L. | 0.130 |
| *Agrimonia eupatoria* L. | 0.090 |
| *Alcea biennis* Winterl | 0.010 |
| *Alchemilla vulgaris* L. | 0.050 |
| *Anthyllis vulneraria* L. | 0.020 |
| *Arctium lappa* L. | 0.130 |
| *Arum maculatum* L. | 0.010 |
| *Asarum europaeum* L. | 0.060 |
| *Asparagus officinalis* L. | 0.020 |
| *Betula pendula* Roth | 0.120 |
| *Calendula officinalis* L. | 0.260 |
| *Centaurium erythraea* Roth. | 0.160 |
| *Chelidonium majus* L. | 0.300 |
| *Cichorium intybus* L. | 0.230 |
| *Cornus mas* L. | 0.450 |
| *Corylus colurna* L. | 0.070 |
| *Cotinus coggygria* Scop. | 0.150 |
| *Dipsacus laciniatus* L. | 0.010 |
| *Epilobium parviflorum* Schreb. | 0.060 |
| *Equisetum arvense* L. | 0.160 |
| *Eupatorium cannabium* L. | 0.060 |
| *Euphrasia officinalis* L. | 0.030 |
| *Filipendula hexapetala* Gilib. | 0.060 |
| *Fragaria vesca* L. | 0.540 |
| *Galium odoratum* (L.) Scop. | 0.040 |
| *Galium verum* L. | 0.260 |
| *Geranium macrorrhizum* L. | 0.060 |
| *Geranium robertianum* L. | 0.070 |
| *Hedera helix* L. | 0.090 |
| *Helleborus odorus* WK | 0.030 |
| *Hieracium pilosella* L. | 0.010 |
| *Humulus lupulus* L. | 0.040 |
| *Hypericum perforatum* L. | 1.300 |
| *Lasterpitium latifolium* L. | 0.040 |
| *Linaria vulgaris* Mill. | 0.050 |
| *Lotus corniculatus* L. | 0.030 |
| *Lysimachia nummularia* L. | 0.010 |
| *Lythrum salicaria* L. | 0.010 |
| *Malus sylvestris* (L.) Mill. | 0.170 |
| *Melilotus albus* Medik. | 0.050 |
| *Melilotus officinalis* (L.) Pall. | 0.070 |
| *Melissa officinalis* L. | 0.520 |
| *Mentha longifolia* (L.) L. | 0.140 |
| *Origanum vulgare* L. | 0.230 |
| *Petasites hybridus* (L.) "G.Gaertn., B.Mey. & Scherb." | 0.110 |
| *Peucedanum longifolium* WK | 0.040 |
| *Pinus nigra* J.F.Arnold. | 0.040 |
| *Plantago lanceolata* L. | 0.060 |
| *Plantago major* L. | 0.580 |
| *Polygonum aviculare* L. | 0.080 |
| *Prunus spinosa* L. | 0.250 |
| *Pulmonaria officinalis* L. | 0.100 |
| *Pyrus pyraster* (L.) Burgst. | 0.070 |
| *Quercus cerris* L. | 0.050 |
| *Robinia pseudoacacia* L. | 0.110 |
| *Rosa canina* L. | 0.920 |
| *Rubus plicatus* Weihe & Nees | 0.730 |
| *Rumex acetosa* L. | 0.050 |
| *Rumex patientia* L. | 0.460 |
| *Salix alba* L. | 0.090 |
| *Sambucus nigra* L. | 1.090 |
| *Satureja subspicata* Bartl. ex Vis. | 0.160 |
| *Sempervivum tectorum* L. | 0.400 |
| *Sorbus aucuparia* L. | 0.110 |
| *Stachys officinalis* (L.) Trevis. | 0.190 |
| *Symphytum officinale* L. | 0.310 |
| *Tanacetum vulgare* L. | 0.050 |
| *Taraxacum* sect. *Ruderalia* | 0.720 |
| *Teucrium chamaedrys* L. | 0.180 |
| *Teucrium montanum* L. | 0.100 |
| *Thymus serphyllum* L. | 0.580 |
| *Tilia platyphyllos* Scop. | 0.610 |
| *Trifolium pratense* L. | 0.070 |
| *Trifolium repens* L. | 0.080 |
| *Tussilago farfara* L. | 0.130 |
| *Urtica dioica* L. | 1.430 |
| *Vaccinium vitis-idaea* L. | 0.150 |
| *Valeriana officinalis* L. | 0.060 |
| *Verbena officinalis* L. | 0.060 |
| *Veronica officinalis* L. | 0.040 |
| *Vicia cracca* L. | 0.020 |

Table 15. Fidelity level for individual plants used in Aleksinac district  (n=55)

| Name of plant | Organ system | Lu | Lp | FL | FLx100 |
| --- | --- | --- | --- | --- | --- |
| *Allium ursinum* L. | CSD | 3 | 3 | 1.00 | 100.00 |
| *Althaea officinalis* L. | ResSD | 3 | 3 | 1.00 | 100.00 |
| *Betula pendula* Roth | USD | 4 | 4 | 1.00 | 100.00 |
| *Crataegus monogyna* Jacq. | CSD | 6 | 6 | 1.00 | 100.00 |
| *Hedera helix* L. | ResSD | 3 | 3 | 1.00 | 100.00 |
| *Equisetum arvense* L. | USD | 7 | 6 | 0.86 | 85.71 |
| *Melissa officinalis* L. | NSD | 9 | 7 | 0.78 | 77.78 |
| *Rosa canina* L. | ISD | 12 | 9 | 0.75 | 75.00 |
| *Urtica dioica* L. | CSD | 19 | 14 | 0.74 | 73.68 |
| *Plantago major* L. | SA | 19 | 13 | 0.68 | 68.42 |
| *Juglans regia* L. | ESD | 9 | 5 | 0.56 | 55.56 |
| *Hypericum perforatum* L. | SA | 50 | 26 | 0.52 | 52.00 |
| *Chelidonium majus* L. | SA | 6 | 3 | 0.50 | 50.00 |
| *Symphytum officinale* L. | SA | 6 | 3 | 0.50 | 50.00 |
| *Tilia platyphyllos* Scop. | ISD | 10 | 5 | 0.50 | 50.00 |
| *Rubus ulmifolius* Schott | ResSD | 7 | 3 | 0.43 | 42.86 |
| *Sambucus nigra* L. | ISD and ResSD | 10 | 4 | 0.40 | 40.00 |
| *Calendula officinalis* L. | SA and CSD | 10 | 3 | 0.30 | 30.00 |
| *Achillea millefolium* L. | USD | 19 | 4 | 0.21 | 21.05 |

Table 16. Fidelity level of individual plants used in for Bor district  (n=100)

| Name of plant | Organ system | Lu | Lp | FL | FLx100 |
| --- | --- | --- | --- | --- | --- |
| *Alchemilla vulgaris* L. | RepSD | 5 | 5 | 1 | 100 |
| *Melilotus albus* Medik. | CSD | 5 | 5 | 1 | 100 |
| *Rumex patientia* L. | DSD | 6 | 6 | 1 | 100 |
| *Mentha longifolia* (L.) L. | DSD | 14 | 13 | 0.93 | 92.86 |
| *Valeriana officinalis* L. | NSD | 7 | 6 | 0.86 | 85.71 |
| *Betula pendula* Roth | USD | 13 | 11 | 0.85 | 84.62 |
| *Pulmonaria officinalis* L. | ResSD | 12 | 10 | 0.83 | 83.33 |
| *Cichorium intybus* L. | DSD | 21 | 17 | 0.81 | 80.95 |
| *Fragaria vesca* L. | DSD | 9 | 7 | 0.78 | 77.78 |
| *Melissa officinalis* L. | NSD | 50 | 37 | 0.74 | 74.00 |
| *Rosa canina* L. | ISD | 56 | 39 | 0.70 | 69.64 |
| *Vaccinium vitis-idaea* L. | USD | 9 | 6 | 0.67 | 66.67 |
| *Symphytum officinale* L. | MSSD | 35 | 23 | 0.66 | 65.71 |
| *Sambucus nigra* L. | ResSD | 73 | 47 | 0.64 | 64.38 |
| *Aesculus hippocastanum* L. | CSD | 14 | 9 | 0.64 | 64.29 |
| *Cornus mas* L. | CSD | 11 | 7 | 0.64 | 63.64 |
| *Urtica dioica* L. | CSD | 73 | 46 | 0.63 | 63.01 |
| *Teucrium chamaedrys* L. | DSD | 23 | 14 | 0.61 | 60.87 |
| *Satureja subspicata* Bartl. ex Vis. | DSD | 16 | 9 | 0.56 | 56.25 |
| *Tussilago farfara* L. | ResSD | 15 | 8 | 0.53 | 53.33 |
| *Plantago major* L. | SA | 62 | 33 | 0.53 | 53.23 |
| *Equisetum arvense* L. | USD | 22 | 11 | 0.50 | 50.00 |
| *Hypericum perforatum* L. | SA | 132 | 64 | 0.48 | 48.48 |
| *Teucrium montanum* L. | DSD | 15 | 7 | 0.47 | 46.67 |
| *Calendula officinalis* L. | SA | 26 | 12 | 0.46 | 46.15 |
| *Achillea clypeolata* Sm. | DSD | 11 | 5 | 0.45 | 45.45 |
| *Petasites hybridus* (L.) ''G.Gaertn., B. Mey. & Scherb''. | CSD and MSSD | 11 | 5 | 0.45 | 45.45 |
| *Centaurium erythraea* Rafn | DSD | 20 | 9 | 0.45 | 45.00 |
| *Tilia platyphyllos* Scop. | ISD | 74 | 33 | 0.45 | 44.59 |
| *Thymus serpyllum* L. | NSD | 61 | 26 | 0.43 | 42.62 |
| *Chelidonium majus* L. | SA | 31 | 13 | 0.42 | 41.94 |
| *Achillea millefolium* L. | DSD | 82 | 32 | 0.39 | 39.02 |
| *Stachys officinalis* (L.)Trevis. | TA | 21 | 8 | 0.38 | 38.10 |
| *Sempervivum tectorum* L. | SS | 38 | 14 | 0.37 | 36.84 |
| *Arctium lappa* L. | MSSD | 14 | 5 | 0.36 | 35.71 |
| *Cotinus coggygria* Scop. | TA | 22 | 6 | 0.27 | 27.27 |
| *Taraxacum* sect. *Ruderalia* | DSD | 41 | 11 | 0.27 | 26.83 |
| *Agrimonia eupatoria* L. | USD | 16 | 4 | 0.25 | 25.00 |
| *Rubus plicatus* Weihe & Nees | CSD and DSD | 20 | 5 | 0.25 | 25.00 |
| *Origanum vulgare* L. | NSD | 23 | 5 | 0.22 | 21.74 |

Table 17. Fidelity level for both, recorded plants of the whole study region  (n=155)

| Name of plant | Organ system | Lu | Lp | FL | FLx100 |
| --- | --- | --- | --- | --- | --- |
| *Alchemilla vulgaris* L. | RepSD | 5 | 5 | 1 | 100 |
| *Crataegus monogyna* Jacq. | CSD | 6 | 6 | 1 | 100 |
| *Melilotus albus* Medik. | CSD | 5 | 5 | 1 | 100 |
| *Rumex patientia* L. | DSD | 6 | 6 | 1 | 100 |
| *Mentha longifolia* (L.) L. | DSD | 15 | 14 | 0.93 | 93.33 |
| *Betula pendula* Roth | USD | 17 | 15 | 0.88 | 88.23 |
| *Valeriana officinalis* L. | NSD | 7 | 6 | 0.86 | 85.71 |
| *Pulmonaria officinalis* L. | ResSD | 13 | 11 | 0.85 | 84.61 |
| *Cichorium intybus* L. | DSD | 24 | 19 | 0.79 | 79.16 |
| *Melissa officinalis* L. | NSD | 59 | 44 | 0.75 | 74.57 |
| *Pinus nigra* J. F. Arnold | ResSD | 7 | 5 | 0.71 | 71.43 |
| *Rosa canina* L. | ISD | 68 | 48 | 0.71 | 70.58 |
| *Vaccinium vitis-idaea* L. | USD | 9 | 6 | 0.67 | 66.67 |
| *Urtica dioica* L. | CSD | 92 | 60 | 0.65 | 65.22 |
| *Aesculus hippocastanum* L. | CSD | 14 | 9 | 0.64 | 64.29 |
| *Sambucus nigra* L. | ResSD | 83 | 51 | 0.61 | 61.45 |
| *Symphytum officinale* L. | MSSD | 41 | 25 | 0.61 | 60.97 |
| *Teucrium chamaedrys* L. | DSD | 24 | 15 | 0.62 | 62.50 |
| *Equisetum arvense* L. | USD | 29 | 17 | 0.59 | 58.62 |
| *Cornus mas* L. | CSD | 12 | 7 | 0.58 | 58.33 |
| *Fragaria vesca* L. | DSD | 12 | 7 | 0.58 | 58.33 |
| *Plantago major* L. | SA | 81 | 46 | 0.57 | 56.79 |
| *Satureja subspicata* Bartl. ex Vis. | DSD | 16 | 9 | 0.56 | 56.25 |
| *Tussilago farfara* L. | ResSD | 16 | 9 | 0.56 | 56.25 |
| *Juglans regia* L. | ESD | 9 | 5 | 0.55 | 55.55 |
| *Hedera helix* L. | ResSD | 12 | 6 | 0.5 | 50 |
| *Hypericum perforatum* L. | SA | 182 | 90 | 0.49 | 49.45 |
| *Teucrium montanum* L. | DSD | 15 | 7 | 0.47 | 46.67 |
| *Achillea clypeolata* Sm. | DSD | 11 | 5 | 0.45 | 45.45 |
| *Centaurium erythraea* Rafn | DSD | 22 | 10 | 0.45 | 45.45 |
| *Petasites hybridus* (L.) ''G.Gaertn., B. Mey. & Scherb''. | CSD and MSSD | 11 | 5 | 0.45 | 45.45 |
| *Tilia platyphyllos* Scop. | ISD | 84 | 38 | 0.45 | 45.24 |
| *Chelidonium majus* L. | SA | 37 | 16 | 0.43 | 43.24 |
| *Thymus serpyllum* L. | NSD | 61 | 26 | 0.47 | 42.62 |
| *Calendula officinalis* L. | SA | 36 | 15 | 0.42 | 41.67 |
| *Plantago lanceolata* L. | ResSD | 13 | 5 | 0.38 | 38.46 |
| *Stachys officinalis* (L.) Trevis. | TA | 21 | 8 | 0.38 | 38.09 |
| *Sempervivum tectorum* L. | SS | 38 | 14 | 0.37 | 36.84 |
| *Arctium lappa* L. | MSSD | 14 | 5 | 0.35 | 35.71 |
| *Achillea millefolium* L. | DSD | 101 | 34 | 0.34 | 33.66 |
| *Agrimonia eupatoria* L. | USD | 17 | 5 | 0.29 | 29.41 |
| *Cotinus coggygria* Scop. | TA | 22 | 6 | 0.27 | 27.27 |
| *Taraxacum* sect. *Ruderalia* | DSD | 41 | 11 | 0.27 | 26.83 |
| *Origanum vulgare* L. | NSD | 27 | 7 | 0.26 | 25.92 |
| *Rubus plicatus* Weihe & Nees | CSD and DSD | 20 | 5 | 0.25 | 25 |

Table 18. Fidelity level for individual plants used by men in Aleksinac district  (n=21)

| Name of plant | Organ system | lu | lp | FL | FLx100 |
| --- | --- | --- | --- | --- | --- |
| *Juglans regi*a L. | ESD | 3 | 3 | 1 | 100 |
| *Urtica dioica* L. | DSD | 5 | 4 | 0.8 | 80 |
| *Hypericum perforatum* L. | SA | 14 | 11 | 0.78 | 78 |
| *Plantago major* L. | SA | 8 | 6 | 0.75 | 75 |

Table 19. Fidelity level for individual plants used by women in Aleksinac district  (n=34)

| Name of plant | Organ system | lu | lp | FL | FLx100 |
| --- | --- | --- | --- | --- | --- |
| *Betula pendula* Roth | USD | 4 | 4 | 1.00 | 100.00 |
| *Crataegus monogyna* Jacq. | CSD | 4 | 4 | 1.00 | 100.00 |
| *Hedera helix* L. | ResSD | 3 | 3 | 1.00 | 100.00 |
| *Equisetum arvense* L. | USD | 7 | 6 | 0.86 | 85.71 |
| *Urtica dioica* L. | CSD | 14 | 10 | 0.71 | 71.43 |
| *Rosa canina* L. | ISD | 10 | 7 | 0.70 | 70.00 |
| *Melissa officinalis* L. | NSD | 9 | 6 | 0.67 | 66.67 |
| *Plantago major* L. | SA | 11 | 7 | 0.64 | 63.64 |
| *Symphytum officinale* L. | SA | 5 | 3 | 0.60 | 60.00 |
| *Chelidonium majus* L. | SA | 6 | 3 | 0.50 | 50.00 |
| *Rubus ulmifolius* Schott | ResSD | 6 | 3 | 0.50 | 50.00 |
| *Tilia platyphyllos* Scop. | ISD | 7 | 3 | 0.43 | 42.86 |
| *Hypericum perforatum* L. | SA | 36 | 15 | 0.42 | 41.67 |
| *Sambucus nigra* L. | ISD | 8 | 3 | 0.38 | 37.50 |
| *Sambucus nigra* L. | ResSD | 8 | 3 | 0.38 | 37.50 |
| *Calendula officinalis* L. | CSD | 9 | 3 | 0.33 | 33.33 |
| *Calendula officinalis* L. | SA | 9 | 3 | 0.33 | 33.33 |

Table 20. Fidelity level for individual plants used by men in Bor district  (n=21)

| Name of plant | Organ system | lu | lp | FL | FLx100 |
| --- | --- | --- | --- | --- | --- |
| *Melissa officinalis* L. | NSD | 9 | 8 | 0.89 | 88.89 |
| *Cichorium intybus* L. | DSD | 6 | 5 | 0.83 | 83.33 |
| *Rosa canina* L. | ISD | 11 | 9 | 0.82 | 81.82 |
| *Mentha longifolia* (L.) L. | DSD | 5 | 4 | 0.80 | 80.00 |
| *Sempervivum tectorum* L. | SS | 10 | 7 | 0.70 | 70.00 |
| *Symphytum officinale* L. | MSSD | 11 | 7 | 0.64 | 63.64 |
| *Satureja subspicata* Bartl. ex Vis. | ISD | 5 | 3 | 0.60 | 60.00 |
| *Plantago major* L. | SA | 12 | 7 | 0.58 | 58.33 |
| *Sambucus nigra* L. | ResSD | 12 | 7 | 0.58 | 58.33 |
| *Urtica dioica* L. | CSD | 14 | 8 | 0.57 | 57.14 |
| *Hypericum perforatum* L. | SA | 22 | 11 | 0.50 | 50.00 |
| *Thymus serpyllum* L. | NSD | 8 | 4 | 0.50 | 50.00 |
| *Taraxacum* sect. *Ruderalia* | DSD | 11 | 5 | 0.45 | 45.45 |
| *Arctium lappa* L. | MSSD | 7 | 3 | 0.43 | 42.86 |
| *Tilia platyphyllos* Scop. | ISD | 15 | 6 | 0.40 | 40.00 |
| *Rubus plicatus* Weihe & Nees | DSD | 8 | 3 | 0.38 | 37.50 |
| *Achillea millefolium* L. | DSD | 13 | 4 | 0.307692 | 30.769231 |

Table 21. Fidelity level for individual plants used by women in Bor district  (n=79)

| Name of plant | Organ system | lu | lp | FL | FLx100 |
| --- | --- | --- | --- | --- | --- |
| *Alchemilla vulgaris* L. | RepSD | 5 | 5 | 1.00 | 100.00 |
| *Epilobium parviflorum* Schreb. | USD | 3 | 3 | 1.00 | 100.00 |
| *Euphrasia officinalis* L. | SS | 3 | 3 | 1.00 | 100.00 |
| *Melilotus albus* Medik. | CSD | 5 | 5 | 1.00 | 100.00 |
| *Mentha longifolia* (L.) L. | DSD | 9 | 9 | 1.00 | 100.00 |
| *Rumex patientia* L. | DSD | 6 | 6 | 1.00 | 100.00 |
| *Betula pendula* Roth | USD | 12 | 10 | 0.83 | 83.33 |
| *Cichorium intybus* L. | DSD | 15 | 12 | 0.80 | 80.00 |
| *Valeriana officinalis* L. | NSD | 5 | 4 | 0.80 | 80.00 |
| *Pulmonaria officinalis* L. | ResSD | 9 | 7 | 0.78 | 77.78 |
| *Satureja subspicata* Bartl. ex Vis. | DSD | 11 | 8 | 0.73 | 72.73 |
| *Fragaria vesca* L. | DSD | 7 | 5 | 0.71 | 71.43 |
| *Melissa officinalis* L. | NSD | 41 | 29 | 0.71 | 70.73 |
| *Rosa canina* L. | ISD | 44 | 30 | 0.68 | 68.18 |
| *Teucrium chamaedrys* L. | DSD | 18 | 12 | 0.67 | 66.67 |
| *Symphytum officinale* L. | MSSD | 24 | 16 | 0.67 | 66.67 |
| *Sambucus nigra* L. | ResSD | 61 | 40 | 0.66 | 65.57 |
| *Urtica dioica* L. | CSD | 59 | 38 | 0.64 | 64.41 |
| *Vaccinium vitis-idaea* L. | USD | 8 | 5 | 0.63 | 62.50 |
| *Linaria vulgaris* Mill. | CSD | 7 | 4 | 0.57 | 57.14 |
| *Equisetum arvense* L. | USD | 16 | 9 | 0.56 | 56.25 |
| *Achillea clypeolata* Sm. | DSD | 9 | 5 | 0.56 | 55.56 |
| *Cornus mas* L. | CSD | 9 | 5 | 0.56 | 55.56 |
| *Plantago major* L. | SA | 50 | 26 | 0.52 | 52.00 |
| *Chelidonium majus* L. | SA | 26 | 13 | 0.50 | 50.00 |
| *Geranium robertianum* L. | CSD | 8 | 4 | 0.50 | 50.00 |
| *Melilotus officinalis* (L.) Pall. | CSD | 8 | 4 | 0.50 | 50.00 |
| *Prunus spinosa* L. | CSD | 8 | 4 | 0.50 | 50.00 |
| *Hypericum perforatum* L. | SA | 109 | 53 | 0.49 | 48.62 |
| *Petasites hybridus* (L.) "G.Gaertn., B.Mey. & Scherb." | CSD | 19 | 9 | 0.47 | 47.37 |
| *Centaurium erythraea* Rafn | DSD | 18 | 8 | 0.44 | 44.44 |
| *Filipendula hexapetala* Gilib. | USD | 7 | 3 | 0.43 | 42.86 |
| *Polygonum aviculare* L. | USD | 7 | 3 | 0.43 | 42.86 |
| *Calendula officinalis* L. | SA | 24 | 10 | 0.42 | 41.67 |
| *Teucrium montanum* L. | DSD | 12 | 5 | 0.42 | 41.67 |
| *Thymus serpyllum* L. | NSD | 53 | 22 | 0.42 | 41.51 |
| *Achillea millefolium* L. | DSD | 69 | 28 | 0.41 | 40.58 |
| *Hedera helix* L. | ResSD | 9 | 3 | 0.33 | 33.33 |
| *Stachys officinalis* (L.)Trevis. | TA | 17 | 5 | 0.29 | 29.41 |
| *Sempervivum tectorum* L. | TA | 28 | 8 | 0.29 | 28.57 |
| *Agrimonia eupatoria* L. | DSD i USD | 16 | 4 | 0.25 | 25.00 |
| *Rubus plicatus* Weihe & Nees | ResSD | 12 | 3 | 0.25 | 25.00 |
| *Cotinus coggygria* Scop. | TA | 21 | 5 | 0.24 | 23.81 |
| *Galium verum* L. | ESD | 13 | 3 | 0.23 | 23.08 |
| *Origanum vulgare* L. | NSD | 19 | 4 | 0.21 | 21.05 |
| *Taraxacum* sect. *Ruderalia* | DSD | 30 | 6 | 0.20 | 20.00 |

Table 22. Fidelity level for individual plants used by men in Aleksinac and Bor districts (n=42)

| Name of plant | Organ system | lu | lp | FL | FLx100 |
| --- | --- | --- | --- | --- | --- |
| *Juglans regia* L. | ESD | 3 | 3 | 1.00 | 100.00 |
| *Pulmonaria officinalis* L. | ResSD | 3 | 3 | 1.00 | 100.00 |
| *Sambucus nigra* L. | ISD | 14 | 14 | 1.00 | 100.00 |
| *Rosa canina* L. | DSD | 13 | 11 | 0.85 | 84.62 |
| *Cichorium intybus* L. | DSD | 6 | 5 | 0.83 | 83.33 |
| *Melissa officinalis* L. | NSD | 12 | 10 | 0.83 | 83.33 |
| *Mentha longifolia* (L.) L. | DSD | 5 | 4 | 0.80 | 80.00 |
| *Plantago major* L. | SA | 8 | 6 | 0.75 | 75.00 |
| *Stachys officinalis* (L.) Trevis. | TA | 4 | 3 | 0.75 | 75.00 |
| *Sempervivum tectorum* L. | SS | 10 | 7 | 0.70 | 70.00 |
| *Symphytum officinale* L. | MSSD | 12 | 8 | 0.67 | 66.67 |
| *Urtica dioica* L. | CSD | 19 | 12 | 0.63 | 63.16 |
| *Satureja subspicata* Bartl. ex Vis. | ISD | 5 | 3 | 0.60 | 60.00 |
| *Hypericum perforatum* L. | SA | 37 | 22 | 0.59 | 59.46 |
| *Plantago major* L. | SA | 12 | 7 | 0.58 | 58.33 |
| *Thymus serpyllum* L. | NSD | 8 | 4 | 0.50 | 50.00 |
| *Taraxacum* sect. *Ruderalia* | DSD | 11 | 5 | 0.45 | 45.45 |
| *Tilia platyphyllos* Scop. | ISD | 18 | 8 | 0.44 | 44.44 |
| *Arctium lappa* L. | MSSD | 7 | 3 | 0.43 | 42.86 |
| *Achillea millefolium* L. | SA | 18 | 5 | 0.28 | 27.78 |

Table 23. Fidelity level for individual plants used by women in Aleksinac and Bor districts (n=113)

| Name of plant | Organ system | lu | lp | FL | FLx100 |
| --- | --- | --- | --- | --- | --- |
| *Alchemilla vulgaris* L. | RepSD | 5 | 5 | 1.00 | 100.00 |
| *Betula pendula* Roth | USD | 4 | 4 | 1.00 | 100.00 |
| *Crataegus monogyna* Jacq. | CSD | 4 | 4 | 1.00 | 100.00 |
| *Epilobium parviflorum* Schreb. | USD | 3 | 3 | 1.00 | 100.00 |
| *Euphrasia officinalis* L. | SS | 3 | 3 | 1.00 | 100.00 |
| *Melilotus albus* Medik. | CSD | 5 | 5 | 1.00 | 100.00 |
| *Mentha longifolia* (L.) L. | DSD | 9 | 9 | 1.00 | 100.00 |
| *Rumex patientia* L. | DSD | 6 | 6 | 1.00 | 100.00 |
| *Betula pendula* Roth | USD | 12 | 10 | 0.83 | 83.33 |
| *Pulmonaria officinalis* L. | ResSD | 10 | 8 | 0.80 | 80.00 |
| *Valeriana officinalis* L. | NSD | 5 | 4 | 0.80 | 80.00 |
| *Cichorium intybus* L. | DSD | 17 | 13 | 0.76 | 76.47 |
| *Satureja subspicata* Bartl. ex Vis. | DSD | 11 | 8 | 0.73 | 72.73 |
| *Melissa officinalis* L. | NSD | 47 | 34 | 0.72 | 72.34 |
| *Rosa canina* L. | ISD | 54 | 37 | 0.69 | 68.52 |
| *Teucrium chamaedrys* L. | DSD | 19 | 13 | 0.68 | 68.42 |
| *Urtica dioica* L. | CSD | 73 | 48 | 0.66 | 65.75 |
| *Equisetum arvense* L. | USD | 23 | 15 | 0.65 | 65.22 |
| *Aesculus hippocastanum* L. | CSD | 11 | 7 | 0.64 | 63.64 |
| *Vaccinium vitis-idaea* L. | USD | 8 | 5 | 0.63 | 62.50 |
| *Sambucus nigra* L. | ResSD | 69 | 43 | 0.62 | 62.32 |
| *Pinus nigra* J.F.Arnold | ResSD | 5 | 3 | 0.60 | 60.00 |
| *Trifolium pratense* L. | TA | 5 | 3 | 0.60 | 60.00 |
| *Symphytum officinale* L. | MSSD | 29 | 17 | 0.59 | 58.62 |
| *Eupatorium cannabinum* L. | MD | 7 | 4 | 0.57 | 57.14 |
| *Linaria vulgaris* Mill. | CSD | 7 | 4 | 0.57 | 57.14 |
| *Achillea clypeolata* Sm. | DSD | 9 | 5 | 0.56 | 55.56 |
| *Centaurium erythraea* Rafn | DSD | 11 | 6 | 0.55 | 54.55 |
| *Plantago major* L. | SA | 61 | 33 | 0.54 | 54.10 |
| *Tussilago farfara* L. | REsSD | 13 | 7 | 0.54 | 53.85 |
| *Chelidonium majus* L. | SA | 32 | 16 | 0.50 | 50.00 |
| *Cornus mas* L. | CSD | 10 | 5 | 0.50 | 50.00 |
| *Fragaria vesca* L. | DSD | 10 | 5 | 0.50 | 50.00 |
| *Geranium robertianum* L. | CSD | 8 | 4 | 0.50 | 50.00 |
| *Hedera helix* L. | ResSD | 12 | 6 | 0.50 | 50.00 |
| *Melilotus officinalis* (L.) Pall. | CSD | 8 | 4 | 0.50 | 50.00 |
| *Petasites hybridus* (L.) "G.Gaertn., B.Mey. & Scherb." | CSD | 10 | 5 | 0.50 | 50.00 |
| *Prunus spinosa* L. | CSD | 8 | 4 | 0.50 | 50.00 |
| *Rubus ulmifolius* Schott | ResSD | 6 | 3 | 0.50 | 50.00 |
| *Hypericum perforatum* L. | SA | 145 | 68 | 0.47 | 46.90 |
| *Tilia platyphyllos* Scop. | ISD | 66 | 30 | 0.45 | 45.45 |
| *Plantago lanceolata* L. | ResSD | 9 | 4 | 0.44 | 44.44 |
| *Filipendula hexapetala* Gilib. | USD | 7 | 3 | 0.43 | 42.86 |
| *Teucrium montanum* L. | DSD | 12 | 5 | 0.42 | 41.67 |
| *Thymus serpyllum* L. | NSD | 53 | 22 | 0.42 | 41.51 |
| *Polygonum aviculare* L. | USD | 10 | 4 | 0.40 | 40.00 |
| *Calendula officinalis* L. | SA | 29 | 11 | 0.38 | 37.93 |
| *Achillea millefolium* L. | DSD | 83 | 29 | 0.35 | 34.94 |
| *Stachys officinalis* (L.) Trevis. | TA | 17 | 5 | 0.29 | 29.41 |
| *Sempervivum tectorum* L. | TA | 28 | 8 | 0.29 | 28.57 |
| *Agrimonia eupatoria* L. | DSD, USD | 16 | 4 | 0.25 | 25.00 |
| *Rubus plicatus* Weihe & Nees | ResSD, CSD | 12 | 3 | 0.25 | 25.00 |
| *Cotinus coggygria* Scop. | TA | 21 | 5 | 0.24 | 23.81 |
| *Origanum vulgare* L. | NSD | 21 | 5 | 0.24 | 23.81 |
| *Galium verum* L. | ESD | 13 | 3 | 0.23 | 23.08 |
| *Taraxacum* sect. *Ruderalia* | DSD, TA | 30 | 6 | 0.20 | 20.00 |

Table 24. Fidelity level for individual plants used by citizens Aleksinac town (n=10)

| Name of plant | Organ system | lu | lp | FL | FLx100 |
| --- | --- | --- | --- | --- | --- |
| *Paliurus spina-christi* Mill. | DSD | 3 | 3 | 1.00 | 100.00 |
| *Symphytum officinale* L. | SA | 5 | 3 | 0.60 | 60.00 |
| *Hypericum perforatum* L. | SA | 9 | 5 | 0.56 | 55.56 |

Table 25. Fidelity level for individual plants used by citizens from surrounding villages in Aleksinac district  (n=45)

| Name of plant | Organ system | lu | lp | FL | FLx100 |
| --- | --- | --- | --- | --- | --- |
| *Althaea officinilis* L. | ResSD | 3 | 3 | 1.00 | 100.00 |
| *Betula pendula* Roth | USD | 3 | 3 | 1.00 | 100.00 |
| *Crataegus monogyna* Jacq. | CSD | 6 | 6 | 1.00 | 100.00 |
| *Hedera helix* L. | ResSD | 3 | 3 | 1.00 | 100.00 |
| *Pinus nigra* J. F. Arnold | ResSD | 3 | 3 | 1.00 | 100.00 |
| *Equisetum arvense* L. | USD | 7 | 6 | 0.86 | 85.71 |
| *Urtica dioica* L. | CSD | 15 | 12 | 0.80 | 80.00 |
| *Melissa officinalis* L. | NSD | 9 | 7 | 0.78 | 77.78 |
| *Juglans regia* L. | ESD | 4 | 3 | 0.75 | 75.00 |
| *Rosa canina* L. | ISD | 12 | 9 | 0.75 | 75.00 |
| *Plantago major* L. | SA | 17 | 11 | 0.65 | 64.71 |
| *Hypericum perforatum* L. | SA | 41 | 21 | 0.51 | 51.22 |
| *Calendula officinalis* L. | SA | 6 | 3 | 0.50 | 50.00 |
| *Chelidonium majus* L. | SA | 6 | 3 | 0.50 | 50.00 |
| *Sambucus nigra* L. | ISD | 8 | 4 | 0.50 | 50.00 |
| *Tilia platyphyllos* Scop. | ISD | 10 | 5 | 0.50 | 50.00 |
| *Rubus ulmifolius* Schott | ResSD | 7 | 3 | 0.43 | 42.86 |

Table 26. Fidelity level for individual plants used by citizens from Bor town (n=50)

| Name of plant | Organ system | lu | lp | FL | FLx100 |
| --- | --- | --- | --- | --- | --- |
| *Cichorium intybus* L. | DSD | 3 | 3 | 1.00 | 100.00 |
| *Sambucus nigra* L. | ResSD | 3 | 3 | 1.00 | 100.00 |
| *Mentha longifolia* (L.) L. | DSD | 5 | 4 | 0.80 | 80.00 |
| *Rosa canina* L. | ISD | 5 | 4 | 0.80 | 80.00 |
| *Sempervivum tectorum* L. | SS | 4 | 3 | 0.75 | 75.00 |
| *Tilia platyphyllos* Scop. | ISD | 4 | 3 | 0.75 | 75.00 |
| *Satureja subspicata* Bartl. ex Vis. | ISD | 5 | 3 | 0.60 | 60.00 |
| *Plantago major* L. | SA | 7 | 4 | 0.57 | 57.14 |
| *Hypericum perforatum* L. | SA | 12 | 6 | 0.50 | 50.00 |

Table 27. Fidelity level for individual plants used by citizens from surrounding villages in Bor district  (n=50)

| Name of plant | Organ system | lu | lp | FL | FLx100 |
| --- | --- | --- | --- | --- | --- |
| *Alchemilla vulgaris* L. | RepSD | 5 | 5 | 1.00 | 100.00 |
| *Epilobium parviflorum* Schreb. | USD | 3 | 3 | 1.00 | 100.00 |
| *Euphrasia officinalis* L. | SS | 3 | 3 | 1.00 | 100.00 |
| *Humulus lupulus* L. | NSD | 3 | 3 | 1.00 | 100.00 |
| *Melilotus albus* Medik. | CSD | 5 | 5 | 1.00 | 100.00 |
| *Mentha longifolia* (L.) L. | DSD | 9 | 9 | 1.00 | 100.00 |
| *Rumex patientia* L. | DSD | 6 | 6 | 1.00 | 100.00 |
| *Valeriana officinalis* L. | NSD | 6 | 5 | 0.83 | 83.33 |
| *Pulmonaria officinalis* L. | ResSD | 11 | 9 | 0.82 | 81.82 |
| *Cichorium intybus* L. | DSD | 17 | 13 | 0.76 | 76.47 |
| *Betula pendula* Roth | DSD | 12 | 9 | 0.75 | 75.00 |
| *Melissa officinalis* L. | NSD | 47 | 35 | 0.74 | 74.47 |
| *Satureja subspicata* Bartl. ex Vis. | DSD | 11 | 8 | 0.73 | 72.73 |
| *Fragaria vesca* L. | DSD | 7 | 5 | 0.71 | 71.43 |
| *Rosa canina* L. | ISD | 50 | 35 | 0.70 | 70.00 |
| *Symphytum officinale* L. | MSSD | 31 | 21 | 0.68 | 67.74 |
| *Teucrium chamaedrys* L. | DSD | 18 | 12 | 0.67 | 66.67 |
| *Vaccinium vitis-idaea* L. | USD | 9 | 6 | 0.67 | 66.67 |
| *Aesculus hippocastanum* L. | CSD | 14 | 9 | 0.64 | 64.29 |
| *Urtica dioica* L. | CSD | 71 | 45 | 0.63 | 63.38 |
| *Sambucus nigra* L. | ResSD | 70 | 44 | 0.63 | 62.86 |
| *Cornus mas* L. | CSD | 10 | 6 | 0.60 | 60.00 |
| *Trifolium pratense* L. | TA | 5 | 3 | 0.60 | 60.00 |
| *Linaria vulgaris* Mill. | CSD | 7 | 4 | 0.57 | 57.14 |
| *Achillea clypeolata* Sm. | DSD | 9 | 5 | 0.56 | 55.56 |
| *Plantago major* L. | SA | 55 | 29 | 0.53 | 52.73 |
| *Equisetum arvense* L. | USD | 19 | 10 | 0.53 | 52.63 |
| *Eupatorium cannabinum* L. | MD | 6 | 3 | 0.50 | 50.00 |
| *Galium odoratum* (L.) Scop. | NSD | 6 | 3 | 0.50 | 50.00 |
| *Geranium robertianum* L. | CSD | 8 | 4 | 0.50 | 50.00 |
| *Melilotus officinalis* (L.) Pall. | CSD | 8 | 4 | 0.50 | 50.00 |
| *Tussilago farfara* L. | ResSD | 14 | 7 | 0.50 | 50.00 |
| *Hypericum perforatum* L. | SA | 120 | 58 | 0.48 | 48.33 |
| *Calendula officinalis* L. | SA | 26 | 12 | 0.46 | 46.15 |
| *Arctium lappa* L. | MSSD | 11 | 5 | 0.45 | 45.45 |
| *Petasites hybridus* (L.) "G.Gaertn., B.Mey. & Scherb." | CSD, MSSD | 11 | 5 | 0.45 | 45.45 |
| *Chelidonium majus* L. | SA | 29 | 13 | 0.45 | 44.83 |
| *Prunus spinosa* L. | CSD | 9 | 4 | 0.44 | 44.44 |
| *Filipendula hexapetala* Gilib. | USD | 7 | 3 | 0.43 | 42.86 |
| *Polygonum aviculare* L. | TA | 7 | 3 | 0.43 | 42.86 |
| *Tilia platyphyllos* Scop. | ISD | 70 | 30 | 0.43 | 42.86 |
| *Thymus serpyllum* L. | NSD | 59 | 25 | 0.42 | 42.37 |
| *Teucrium montanum* L. | DSD | 12 | 5 | 0.42 | 41.67 |
| *Achillea millefolium* L. | DSD | 79 | 32 | 0.41 | 40.51 |
| *Centaurium erythraea* Rafn | DSD | 20 | 8 | 0.40 | 40.00 |
| *Stachys officinalis* (L.)Trevis. | TA | 20 | 7 | 0.35 | 35.00 |
| *Hedera helix* L. | ResSD | 9 | 3 | 0.33 | 33.33 |
| *Sempervivum tectorum* L. | SS | 34 | 11 | 0.32 | 32.35 |
| *Taraxacum* sect. *Ruderalia* | DSD | 38 | 10 | 0.26 | 26.32 |
| *Agrimonia eupatoria* L. | DSD, USD | 16 | 4 | 0.25 | 25.00 |
| *Cotinus coggygria* Scop. | TA | 21 | 5 | 0.24 | 23.81 |
| *Rubus plicatus* Weihe & Nees | DSD,  ResSD | 17 | 4 | 0.24 | 23.53 |
| *Galium verum* L. | ESD | 13 | 3 | 0.23 | 23.08 |
| *Origanum vulgare* L. | NSD | 22 | 4 | 0.18 | 18.18 |

Table 28. Fidelity level for individual plants used by citizens from Aleksinac and Bor towns  (n=60)

| Name of plant | Organ system | lu | lp | FL | FLx100 |
| --- | --- | --- | --- | --- | --- |
| *Alchemilla vulgaris* L. | RepSD | 4 | 4 | 1.00 | 100.00 |
| *Euphrasia officinalis* L. | SS | 3 | 3 | 1.00 | 100.00 |
| *Melilotus albus* Medik. | CSD | 4 | 4 | 1.00 | 100.00 |
| *Paliurus spina-christi* Mill. | DSD | 3 | 3 | 1.00 | 100.00 |
| *Rumex patientia* L. | DSD | 3 | 3 | 1.00 | 100.00 |
| *Valeriana officinalis* L. | NSD | 4 | 4 | 1.00 | 100.00 |
| *Mentha longifolia* (L.) L. | DSD | 14 | 13 | 0.93 | 92.86 |
| *Fragaria vesca* L. | DSD | 7 | 6 | 0.86 | 85.71 |
| *Betula pendula* Roth | USD | 12 | 10 | 0.83 | 83.33 |
| *Trifolium pratense* L. | TA | 5 | 4 | 0.80 | 80.00 |
| *Cichorium intybus* L. | DSD | 14 | 11 | 0.79 | 78.57 |
| *Pulmonaria officinalis* L. | ResSD | 9 | 7 | 0.78 | 77.78 |
| *Rosa canina* L. | ISD | 32 | 23 | 0.72 | 71.88 |
| *Sambucus nigra* L. | ResSD | 39 | 28 | 0.72 | 71.79 |
| *Melissa officinalis* L. | NSD | 23 | 16 | 0.70 | 69.57 |
| *Vaccinium vitis-idaea* L. | USD | 6 | 4 | 0.67 | 66.67 |
| *Urtica dioica* L. | CSD | 40 | 26 | 0.65 | 65.00 |
| *Cornus mas* L. | CSD | 8 | 5 | 0.63 | 62.50 |
| *Teucrium chamaedrys* L | DSD | 29 | 18 | 0.62 | 62.07 |
| *Aesculus hippocastanum* L. | CSD | 10 | 6 | 0.60 | 60.00 |
| *Hedera helix* L. | ResSD | 5 | 3 | 0.60 | 60.00 |
| *Epilobium parviflorum* Schreb. | USD | 7 | 4 | 0.57 | 57.14 |
| *Eupatorium cannabinum* L. | MD | 7 | 4 | 0.57 | 57.14 |
| *Petasites hybridus* (L.) "G.Gaertn., B.Mey. & Scherb." | CSD | 7 | 4 | 0.57 | 57.14 |
| *Tussilago farfara* L. | ResSD | 11 | 6 | 0.55 | 54.55 |
| *Linaria vulgaris* Mill. | CSD | 6 | 3 | 0.50 | 50.00 |
| *Satureja subspicata* Bartl. ex Vis. | DSD | 14 | 7 | 0.50 | 50.00 |
| *Stachys officinalis* (L.) Trevis. | TA | 10 | 5 | 0.50 | 50.00 |
| *Tilia platyphyllos* Scop. | ISD | 37 | 18 | 0.49 | 48.65 |
| *Hypericum perforatum* L. | SA | 84 | 40 | 0.48 | 47.62 |
| *Centaurium erythraea* Rafn | DSD | 17 | 8 | 0.47 | 47.06 |
| *Chelidonium majus* L. | SA | 15 | 7 | 0.47 | 46.67 |
| *Equisetum arvense* L. | USD | 13 | 6 | 0.46 | 46.15 |
| *Symphytum officinale* L. | MSSD | 24 | 11 | 0.46 | 45.83 |
| *Thymus serpyllum* L. | NSD | 31 | 14 | 0.45 | 45.16 |
| *Plantago major* L. | SA | 43 | 19 | 0.44 | 44.19 |
| *Geranium robertianum* L. | CSD | 7 | 3 | 0.43 | 42.86 |
| *Melilotus officinalis* (L.) Pall. | CSD | 7 | 3 | 0.43 | 42.86 |
| *Prunus spinosa* L. | CSD | 7 | 3 | 0.43 | 42.86 |
| *Filipendula hexapetala* Gilib. | CSD, USD | 8 | 3 | 0.38 | 37.50 |
| *Polygonum aviculare* L. | USD | 8 | 3 | 0.38 | 37.50 |
| *Rubus plicatus* Weihe & Nees | CSD | 14 | 5 | 0.36 | 35.71 |
| *Plantago lanceolata* L. | ResSD | 9 | 3 | 0.33 | 33.33 |
| *Agrimonia eupatoria* L. | USD | 16 | 5 | 0.31 | 31.25 |
| *Calendula officinalis* L. | SA | 23 | 7 | 0.30 | 30.43 |
| *Sempervivum tectorum* L. | SS | 24 | 7 | 0.29 | 29.17 |
| *Taraxacum* sect. *Ruderalia* | DSD | 21 | 6 | 0.29 | 28.57 |
| *Achillea millefolium* L. | DSD | 48 | 13 | 0.27 | 27.08 |
| *Origanum vulgare* L. | NSD | 15 | 4 | 0.27 | 26.67 |
| *Galium verum* L. | ESD, USD | 13 | 3 | 0.23 | 23.08 |

Table 29. Fidelity level for individual plants used by citizens from surrounding villages in Aleksinac and Bor districts  (n=95)

| Name of plant | Organ system | lu | lp | FL | FLx100 |
| --- | --- | --- | --- | --- | --- |
| *Althaea officinilis* L. | ResSD | 3 | 3 | 1.00 | 100.00 |
| *Betula pendula* Roth | USD | 5 | 5 | 1.00 | 100.00 |
| *Crataegus monogyna* Jacq. | CSD | 6 | 6 | 1.00 | 100.00 |
| *Pinus nigra* J. F. Arnold | ResSD | 3 | 3 | 1.00 | 100.00 |
| *Pulmonaria officinalis* L. | ResSD | 4 | 4 | 1.00 | 100.00 |
| *Rumex patientia* L. | DSD | 3 | 3 | 1.00 | 100.00 |
| *Symphytum officinale* L. | MSSD | 17 | 14 | 0.82 | 82.35 |
| *Cichorium intybus* L. | DSD | 10 | 8 | 0.80 | 80.00 |
| *Melissa officinalis* L. | NSD | 36 | 28 | 0.78 | 77.78 |
| *Achillea clypeolata* Sm. | DSD | 4 | 3 | 0.75 | 75.00 |
| *Aesculus hippocastanum* L. | CSD | 4 | 3 | 0.75 | 75.00 |
| *Juglans regia* L. | ESD | 4 | 3 | 0.75 | 75.00 |
| *Petasites hybridus* (L.) "G.Gaertn., B.Mey. & Scherb." | MSSD | 4 | 3 | 0.75 | 75.00 |
| *Plantago major* L. | SA | 38 | 27 | 0.71 | 71.05 |
| *Rosa canina* L. | ISD | 36 | 25 | 0.69 | 69.44 |
| *Equisetum arvense* L. | USD | 16 | 11 | 0.69 | 68.75 |
| *Arctium lappa* L. | MSSD | 6 | 4 | 0.67 | 66.67 |
| *Urtica dioica* L. | CSD | 52 | 34 | 0.65 | 65.38 |
| *Calendula officinalis* L. | SA | 13 | 8 | 0.62 | 61.54 |
| *Tussilago farfara* L. | ResSD | 5 | 3 | 0.60 | 60.00 |
| *Teucrium chamaedrys* L. | DSD | 7 | 4 | 0.57 | 57.14 |
| *Sambucus nigra* L. | ResSD | 44 | 23 | 0.52 | 52.27 |
| *Hypericum perforatum* L. | SA | 97 | 50 | 0.52 | 51.55 |
| *Sempervivum tectorum* L. | SS | 14 | 7 | 0.50 | 50.00 |
| *Cotinus coggygria* Scop. | TA | 9 | 4 | 0.44 | 44.44 |
| *Hedera helix* L. | ResSD | 7 | 3 | 0.43 | 42.86 |
| *Rubus ulmifolius* Schott | ResSD | 7 | 3 | 0.43 | 42.86 |
| *Tilia platyphyllos* Scop. | ISD | 47 | 20 | 0.43 | 42.55 |
| *Chelidonium majus* L. | SA | 22 | 9 | 0.41 | 40.91 |
| *Thymus serpyllum* L. | NSD | 30 | 12 | 0.40 | 40.00 |
| *Achillea millefolium* L. | DSD | 53 | 21 | 0.40 | 39.62 |
| *Stachys officinalis* (L.)Trevis. | SA | 11 | 4 | 0.36 | 36.36 |
| *Origanum vulgare* L. | DSD | 12 | 4 | 0.33 | 33.33 |
| *Taraxacum* sect. *Ruderalia* | DSD | 20 | 5 | 0.25 | 25.00 |
